# Resolution-dependent self-supervised transfer in chest radiograph classification

**DOI:** 10.1038/s43856-026-01897-9

**Published:** 2026-09-09

**Authors:** Soroosh Tayebi Arasteh, Mina Shaigan, Christiane Kuhl, Jakob Nikolas Kather, Sven Nebelung, Daniel Truhn

**Affiliations:** 1 Lab for AI in Medicine, RWTH Aachen University, Aachen, Germany; 2 Department of Diagnostic and Interventional Radiology, University Hospital RWTH Aachen, Aachen, Germany; 3 Department of Urology, Stanford University, Stanford, CA USA; 4 Department of Radiology, Stanford University, Stanford, CA USA; 5 Institute for Computational Genomics, Joint Research Center for Computational Biomedicine, University Hospital RWTH Aachen, Aachen, Germany; 6 Else Kroener Fresenius Center for Digital Health, Technical University Dresden, Dresden, Germany; 7 Department of Medicine I, University Hospital Dresden, Dresden, Germany; 8 National Center for Tumor Diseases (NCT), University Hospital Heidelberg, Heidelberg, Germany

**Keywords:** Radiography, Diagnosis

## Abstract

**Background:**

Self-supervised learning (SSL) has improved visual representation learning, but its value in chest radiography remains uncertain. DINOv3 extends earlier SSL models through Gram-anchored self-distillation and explicit high-resolution adaptation. Whether these changes improve transfer learning for chest radiograph classification has not been established.

**Methods:**

We benchmarked DINOv3 against DINOv2 and supervised ImageNet initialization across seven chest radiograph datasets comprising 816,183 radiographs from pediatric and adult cohorts. ViT-B/16 and ConvNeXt-B were evaluated under full fine-tuning at 224 × 224 and 512 × 512 pixels, with targeted 1024 × 1024 experiments on three cohorts. Additional analyses examined parameter-efficient adaptation, synthetic label corruption, external validation, frozen 7B features, and computational efficiency. The primary outcome was the mean area under the receiver operating characteristic curve across labels.

**Results:**

In adult cohorts, DINOv3 did not consistently outperform DINOv2 at 224 × 224 pixels, but became the strongest initialization at 512 × 512 pixels, especially with ConvNeXt-B. Gains were greatest for small focal and boundary-dependent abnormalities, whereas large-structure findings changed little. The pediatric cohort showed no significant benefit from DINOv3, higher resolution, or backbone choice. Scaling to 1024 × 1024 rarely improved performance and markedly increased computational cost. ConvNeXt-B remained superior to ViT-B/16 under both full and parameter-efficient adaptation. External validation preserved the 512 × 512 DINOv3 advantage, whereas synthetic label corruption showed that this benefit should not be interpreted simply as superior noise robustness. Frozen DINOv3-7B features underperformed relative to fully adapted 86 to 89M-parameter backbones.

**Conclusions:**

For adult chest radiograph classification, DINOv3 provides its most reliable benefit at 512 × 512 pixels, particularly with ConvNeXt-B. Fully adapted mid-sized models at 512 × 512 pixels provided the best performance-cost trade-off in our benchmark.

## Plain language summary

Chest X-rays are one of the most common medical imaging tests, but small or subtle abnormalities can still be difficult for artificial intelligence (AI) systems to detect. We studied DINOv3, an AI approach that learns from large collections of unlabeled images before being adapted to a medical task. Using 816,183 chest X-rays from seven datasets, we found that DINOv3 worked best for adults when images were analyzed at 512 × 512 pixels and the model was fully adapted to the task. The biggest gains were seen for subtle findings such as small focal lesions and abnormalities near organ boundaries. In children, DINOv3 and higher resolution provided little added benefit. Increasing image size further to 1024 × 1024 rarely improved accuracy enough to justify the much higher computing cost, and very large frozen AI models performed worse than smaller models that were fully fine-tuned. Overall, these results suggest that for adult chest X-ray analysis, fully adapted mid-sized models at 512 × 512 pixels offer the best balance of accuracy and efficiency.

## Abstract (web summary)

Tayebi Arasteh et al., benchmark DINOv3, DINOv2, and supervised ImageNet initialization across 816,183 pediatric and adult chest radiographs, evaluating different architectures, resolutions, adaptation strategies, and validation settings for chest radiograph classification. DINOv3 provides the most consistent performance gains in adult cohorts at 512×512 pixels, particularly with ConvNeXt-B and for small focal abnormalities, while 1024×1024 scaling offers little added benefit and fully adapted mid-sized models at 512×512 deliver the best performance-cost trade-off.

## Introduction

Chest radiography is the most widely performed imaging examination worldwide and a first-line tool for detecting pulmonary and cardiac abnormalities. Subtle or low-contrast findings, such as interstitial lung disease, reticular changes, or diffuse pulmonary opacification, can be difficult to recognize, motivating the use of automated analysis to assist interpretation and triage. Artificial intelligence (AI) has become an integral component of medical imaging^1–3^, with chest radiographs serving as one of the most extensively studied modalities for evaluating new algorithms^4–6^. Early advances relied on supervised deep learning, where models were pretrained on large annotated datasets, such as ImageNet^7,8^, and then fine-tuned for radiographic tasks. Although this strategy improved performance compared with training from scratch, it remains constrained by the domain mismatch between natural and medical images and by its dependence on costly manual annotations. Constructing large, expertly labeled radiograph collections continues to be a major bottleneck, motivating the exploration of label-efficient alternatives.

Self-supervised learning (SSL) has emerged as a promising response to this challenge. By constructing pretraining objectives that do not depend on manual labels, SSL enables the use of massive unlabeled datasets to learn transferable visual representations^9,10^. Methods such as MoCo^11^, SimCLR^12^, BYOL^13^, and SwAV^14^ have shown strong performance on natural images, and their application to medical imaging has yielded encouraging gains in classification and segmentation tasks. However, many medical studies remain limited in scale, often involving tens of thousands of radiographs instead of hundreds of thousands of radiographs, and important questions remain regarding the robustness, generalizability, and practical operating conditions of SSL for clinical imaging^15^. These questions are especially relevant in chest radiography, where diagnostically important structures may occupy only a small fraction of the image and where label quality varies widely across datasets.

The introduction of transformer-based^16^ architectures has further accelerated progress. Vision transformers (ViTs)^17^ and modern convolutional backbones, such as ConvNeXt^18^, have reshaped representation learning in computer vision, and their transfer to radiology has highlighted the value of flexible, high-capacity architectures for medical data. Within this line of work, the DINO^19^ family of SSL methods has been particularly influential. DINOv2^20^, pretrained on hundreds of millions of natural images, established itself as a strong general-purpose representation learner. In our prior work^21^, we showed that DINOv2 could match or surpass supervised ImageNet pretraining for chest radiograph classification. Meta’s DINOv3^22^ builds on this foundation by introducing Gram-anchored self-distillation and explicit high-resolution adaptation, with the goal of preserving fine-grained visual information and improving scaling to larger input sizes. Recent studies^23^ have begun to benchmark DINOv3 across multiple medical imaging tasks and modalities^24,25^, including chest radiograph classification, but important questions remain regarding its task-specific operating conditions and interpretation in chest radiography. These design choices are especially relevant to chest radiographs, which are acquired at far higher native resolution than is typically used during downstream model training. Yet several clinically important questions remain unresolved: whether DINOv3’s benefits emerge only at higher resolution, whether they generalize beyond within-dataset splits, whether they extend equally across adult and pediatric cohorts, whether they depend on backbone architecture or adaptation regime, and whether performance gains on weakly labeled datasets reflect better transfer or simply greater tolerance to label noise.

**Fig. 1:**
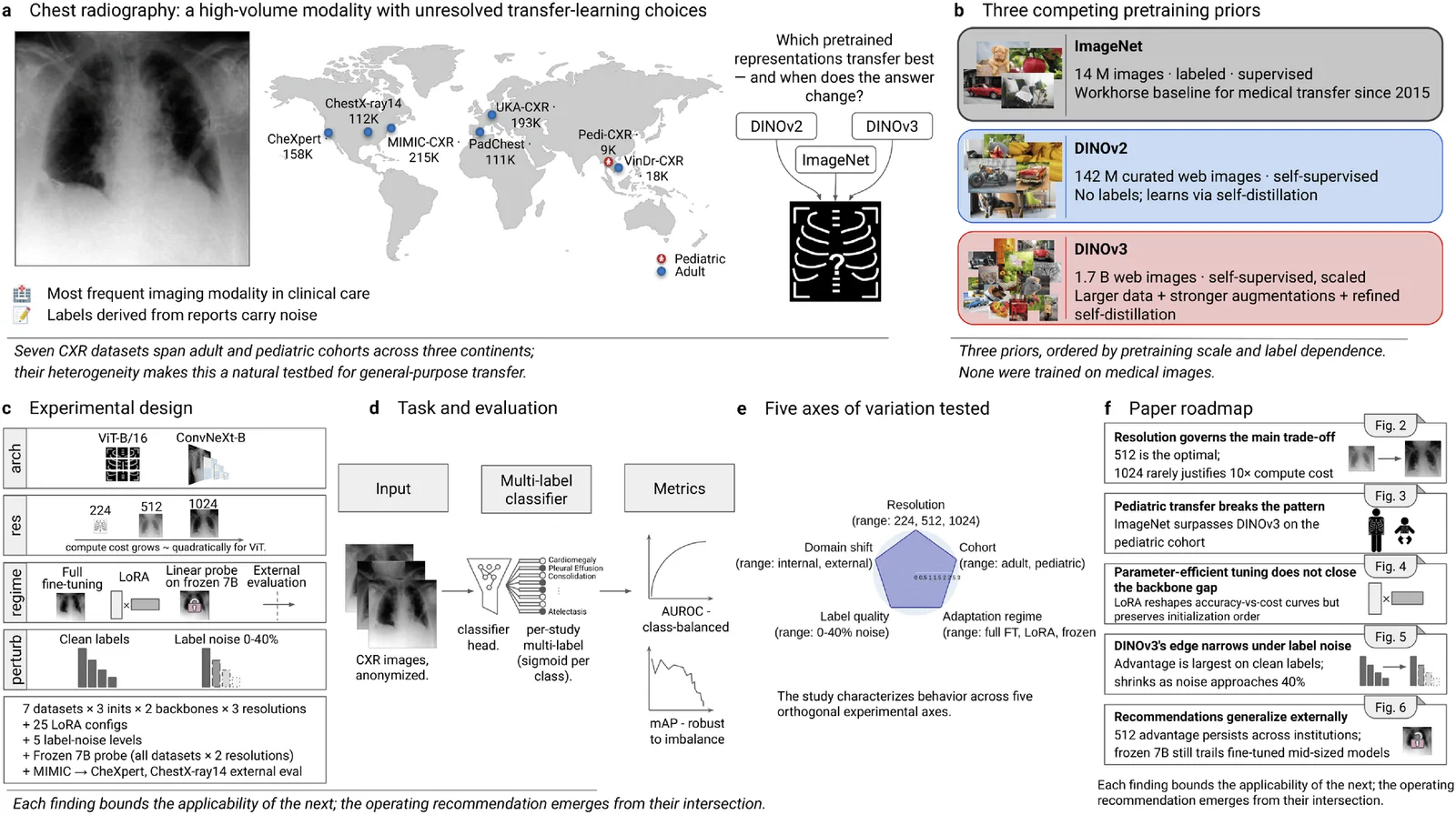
Study overview and analytical framework. **a** Chest radiography as a high-volume imaging modality, and the geographic distribution of the seven study cohorts (*n* = 9125 for Pedi-CXR, 18,000 for VinDr-CXR, 112,120 for ChestX-ray14, 110,525 for PadChest, 157,865 for CheXpert, 215,187 for MIMIC-CXR and 193,361 for UKA-CXR). Red markers denote the pediatric cohort and blue markers the adult cohorts. **b** The three pretraining priors compared here: ImageNet, a labeled supervised natural-image prior; DINOv2, a self-supervised web-scale prior; and DINOv3, a larger self-supervised web-scale prior. None was pretrained on medical images. **c** The experimental design, which varies architecture (ViT-B/16 and ConvNeXt-B), input resolution (224 × 224, 512 × 512, and a targeted 1024 × 1024 pixels), adaptation regime (full fine-tuning, LoRA, frozen DINOv3-7B features and external evaluation), and label condition (clean labels and 0–40% synthetic label noise). **d** The downstream task, per-study multilabel classification with one sigmoid output per class, assessed by AUROC and mAP. **e** The five axes of variation examined: resolution, cohort type, adaptation regime, label quality, and domain shift. **f** Roadmap of the paper. AUROC, area under the receiver operating characteristic curve; LoRA, low-rank adaptation; mAP, mean average precision.

Here, we present a large-scale, chest-radiograph-focused evaluation of DINOv3 for chest radiograph classification across seven datasets comprising 816,183 anteroposterior (AP) or posteroanterior (PA) radiographs from public and internal cohorts (see Fig. 1). Our benchmark spans pediatric and adult populations, two backbone families (ViT-B/16 and ConvNeXt-B), and input resolutions from 224 ×  224 to 1024 × 1024 pixels, with the 1024-pixel setting used as a targeted probe of further scaling. In addition to the core comparison against DINOv2 and ImageNet initialization, we examine frozen representations from the 7B-parameter DINOv3 teacher, test whether the ConvNeXt-B vs. ViT-B/16 difference persists under parameter-efficient adaptation, assess the effect of controlled synthetic label corruption, and perform external validation across institutions using a harmonized label space. This design allows us to move beyond a simple benchmark and ask when DINOv3 helps, where it fails, and how those gains should be interpreted in practice. As we show, DINOv3 yields its most reproducible benefit in adult cohorts at 512 × 512 pixels, especially with ConvNeXt-B, whereas the pediatric cohort follows a distinct transfer pattern: scaling beyond 512 × 512 provides little additional value for markedly higher cost, and frozen billion-parameter features remain inferior to task-adapted mid-sized models. We expect that these results provide practical guidance for applying modern SSL to chest radiograph analysis and clarify the conditions under which high-resolution self-supervised representations are most useful in radiology.

## Methods

### Ethics statement

The experiments were performed in accordance with relevant national and international guidelines and regulations. Approval for the UKA-CXR dataset by the Ethical Committee of the Medical Faculty of RWTH Aachen University has been granted for this retrospective study (Reference No. EK 22-319). The institutional review board did not require informed consent from subjects and/or their legal guardian(s) as this was a retrospective study.

The other six cohorts are previously collected datasets, released in de-identified form. Pedi-CXR was approved by the institutional review board of Phu Tho Obstetric & Pediatric Hospital in Vietnam, and informed patient consent was waived. VinDr-CXR was approved by the institutional review boards of Hospital 108 and Hanoi Medical University Hospital in Vietnam, and informed patient consent was waived. ChestX-ray14 was released publicly by the National Institutes of Health Clinical Center after screening to remove all personally identifiable information. Its source publication names no approving ethics committee and states nothing about informed consent. PadChest was approved by an institutional research committee that its source publication does not name, and that publication states nothing about informed consent. Its images and reports were anonymized and de-identified by the Medical Image Bank of the Valencian Community and the health informatics department of Hospital Universitario de San Juan in Alicante, Spain. CheXpert was reviewed and approved by the Stanford Hospital institutional review board, and individual patient consent was waived for the de-identified data. MIMIC-CXR was approved by the institutional review board of Beth Israel Deaconess Medical Center, and individual patient consent was waived. All six datasets were used in this study under their respective licenses and data use agreements. No additional ethics approval or informed consent was required for this secondary analysis.

### Patient datasets

This study included a total of *n* = 816,183 AP or PA chest radiographs from seven international cohorts encompassing both adult and pediatric populations. Patients ranged in age from infancy to over 111 years. The datasets span diverse geographic regions (Asia, Europe, and North America), label generation strategies (manual annotation, rule-based natural language processing (NLP), and hybrid approaches), and clinical contexts (inpatient, outpatient, intensive care, and pediatrics). A detailed overview of dataset characteristics is provided in Table 1. Below, we describe each dataset. For datasets with predefined train and test partitions but without patient identifiers (Pedi-CXR and VinDr-CXR), no additional validation split was created in order to avoid potential patient-level information leakage.

**Table 1:** Characteristics of the datasets and study protocol utilized in this study

Field	Pedi-CXR	VinDr-CXR	ChestX-ray14	PadChest	CheXpert	MIMIC-CXR	UKA-CXR
Cohort type	Pediatric	Adult	Adult	Adult	Adult	Adult	Adult
Age (years) Mean ± SD Range	4 ± 3 (0^a^, 10)	54 ± 18 (2, 90)	46 ± 17 (1, 95)	56 ± 21 (0^a^, 105)	60 ± 18 (18, 90)	N/A	66 ± 16 (0^a^, 111)
Sex (female/male)	42%/58%	47%/53%	44%/56%	50%/50%	41%/59%	N/A	35%/65%
Number of patients (*n*)	N/A	N/A	30,805	67,205	57,872	62,094	54,176
Number of radiographs (*n*) Total Training Validation Test	9125 6955 0 1397	18,000 15,000 0 3000	112,120 77,870 8654 25,596	110,525 79,697 8783 22,045	157,865 115,449 13,098 29,318	215,187 153,255 18,139 43,793	193,361 137,902 15,353 40,106
Number of labels used (*n*)	3	11	14	17	10	10	6
Labels used in the main study	No finding, pneumonia, bronchitis/bronchiolitis	Cardiomegaly, pleural effusion, pneumonia, atelectasis, no finding, consolidation, pneumothorax, pleural thickening, lung opacity, pulmonary fibrosis, nodule/mass	Cardiomegaly, effusion, pneumonia, atelectasis, no finding, consolidation, pneumothorax, fibrosis, emphysema, hernia, pleural thickening, edema, nodule, mass	Cardiomegaly, pleural effusion, pneumonia, atelectasis, no finding, consolidation, pneumothorax, emphysema, hernia, scoliosis, congestion, aortic elongation, kyphosis, COPD signs, pleural thickening, nodule, mass, infiltrates	Cardiomegaly, pleural effusion, pneumonia, atelectasis, no finding, consolidation, pneumothorax, lung opacity, lung lesion, fracture	Cardiomegaly, pleural effusion, pneumonia, atelectasis, no finding, consolidation, pneumothorax, lung opacity, lung lesion, fracture	Cardiomegaly, congestion, pleural effusion, pneumonic infiltrates, atelectasis, no finding
Label provenance	Radiologist annotation	Radiologist annotation	Rule-based NLP from reports	Partially radiologist annotation + partially rule-based NLP	Rule-based NLP from reports	Rule-based NLP from reports	Radiologist annotation
Location	Vietnam	Vietnam	USA	Spain	USA	USA	Germany
Projections (AP/PA)	0%/100%	0%/100%	40%/60%	17%/83%	84%/16%	58%/42%	47%/53%
Public or internal	Public	Public	Public	Public	Public	Public	Internal

Summary of patient cohorts, image counts, demographics, and label sets for all seven datasets: Pedi-CXR, VinDr-CXR, ChestX-ray14, PadChest, CheXpert, MIMIC-CXR, and UKA-CXR. Reported values include the number of patients and radiographs, split into total, training, validation, and test sets, as well as patient age distributions (mean ± standard deviation (SD) and range) and sex ratios (female/male). AP or PA images are considered in this study. For Pedi-CXR and VinDr-CXR, no validation split was used because patient identifiers were unavailable, precluding leakage-safe subdivision of the provided training sets. For Pedi-CXR, 773 images of the released training set were not used in any experiment, so its training, validation, and test counts do not sum to the cohort total.

*N/A* not available.

^a^The youngest patients in the Pedi-CXR, PadChest, and UKA-CXR datasets were infants younger than 6 months. Missing demographic information was handled by exclusion from the corresponding analyses: age information was unavailable for 29 patients in UKA-CXR, 10 patients in ChestX-ray14, 13,772 images in VinDr-CXR, and one image in CheXpert, while sex information was unavailable for 9392 images in VinDr-CXR.

### Pedi-CXR dataset

The Pedi-CXR^26^ dataset is the largest publicly available pediatric chest radiograph dataset with diagnostic labels. It contains 9125 PA images from children under the age of 10 years, collected in Vietnam. All radiographs were manually annotated by three radiologists with at least 10 years of experience. For this study, we used the dataset’s original test set (*n* = 1397) for evaluation and trained on 6955 images; the remaining 773 images of the released training set were not used in any experiment. No separate validation split was used (see Table 1).

### VinDr-CXR dataset

The VinDr-CXR^27^ dataset comprises 18,000 adult radiographs, curated from more than 100,000 studies performed at two Vietnamese hospitals. Images were acquired on equipment from multiple manufacturers. Labeling was performed by 17 radiologists, with each image independently annotated by three experts. The dataset authors provided a split into *n* = 15,000 training and *n* = 3000 test images, which we used directly. No separate validation split was created, because the dataset does not provide patient identifiers and therefore additional random sampling from the training set could have introduced patient-level information leakage across splits. Labels cover common thoracic diseases, such as cardiomegaly, effusion, and pneumonia (see Table 1).

### ChestX-ray14 dataset

The ChestX-ray14^28^ dataset, released by the National Institutes of Health, contains *n* = 112,120 radiographs from 30,805 patients. Fourteen thoracic pathologies were labeled using a two-stage NLP pipeline applied to corresponding radiology reports. Following prior work, we first generated a patient-wise 77%/23% train-test split and then reserved 10% of the training portion for validation, resulting in *n* = 77,870 training, *n* = 8654 validation, and *n* = 25,596 test images. Labels span major cardiopulmonary conditions (see Table 1).

### PadChest dataset

The PadChest^29^ dataset includes *n* = 110,525 AP or PA radiographs from 67,205 patients at Hospital Universitario de San Juan in Alicante, Spain. Labels were derived from radiology reports in Spanish: 27,593 studies were manually annotated by radiologists, and the remainder were automatically labeled using a text classifier trained on this subset. We performed a patient-wise 80%/20% split, stratified by manual vs. automatic labeling, and then reserved 10% of the training portion for validation, yielding *n* = 79,697 training, *n* = 8783 validation, and *n* = 22,045 test images. Labels are diverse and include both common and less frequent findings (see Table 1).

### CheXpert dataset

The CheXpert^30^ dataset consists of *n* = 157,865 AP or PA chest radiographs from 57,872 patients at Stanford Hospital in California, USA. Labels for common radiographic findings were extracted using a rule-based NLP system that categorized mentions as positive, negative, or uncertain. Following established practice, uncertain and negative mentions were grouped as negative. We used a patient-wise 79%/19% split and then reserved 10% of the training portion for validation, resulting in *n* = 115,449 training, *n* = 13,098 validation, and *n* = 29,318 test images. Labels include cardiomegaly, effusion, pneumonia, and others (see Table 1).

### MIMIC-CXR dataset

The MIMIC-CXR^6^ dataset contains *n* = 215,187 AP or PA radiographs from 62,094 patients at Beth Israel Deaconess Medical Center in Boston, Massachusetts, USA, collected between 2011 and 2016. Images were de-identified and linked to associated reports. Labels were generated automatically using the same NLP system as CheXpert^30^, ensuring consistency. We created a patient-wise 80%/20% split and then reserved ~10% of the training portion for validation, yielding *n* = 153,255 training, *n* = 18,139 validation, and *n* = 43,793 test images. Labels overlap with CheXpert, covering major cardiopulmonary findings (see Table 1).

### UKA-CXR dataset

The UKA-CXR^21,31–35^ dataset is an internal cohort from University Hospital RWTH Aachen in Aachen, Germany. It includes *n* = 193,361 adult radiographs from 54,176 patients, collected between 2009 and 2020 across 10 intensive care units using 18 radiography systems. Images were labeled by radiologists within the clinical reporting workflow using a structured template with categories, such as pleural effusion, pneumonia, atelectasis, congestion, and cardiomegaly. For this study, we defined a patient-wise 79%/21% split and then reserved 10% of the training portion for validation, yielding *n* = 137,902 training, *n* = 15,353 validation, and *n* = 40,106 test images. Labels reflect routine diagnostic categories from clinical reporting (see Table 1).

### Label system and preprocessing

As in our prior works^21,31–37^, all datasets were mapped into a unified binary multilabel classification framework, in which each image was assigned a positive or negative label for every included condition. Only AP or PA views were used in all experiments. Pedi-CXR, VinDr-CXR, ChestX-ray14, and PadChest were provided in binary format by design and were used directly. In CheXpert, and consequently in MIMIC-CXR, the original four categories (“positive,” “negative,” “uncertain,” and “not mentioned”) were reduced to binary by treating “negative,” “uncertain,” and “not mentioned” as negative, and only “positive” as positive. For the UKA-CXR dataset, which contained multiple severity levels, “normal” and “uncertain” were classified as negative, whereas all severity categories above normal were classified as positive, for example, “mild,” “moderate,” or “severe” for pleural effusion and “borderline,” “enlarged,” or “massively enlarged” for cardiomegaly. In addition, UKA-CXR contained separate left- and right-sided labels for several findings; in these cases, the presence of a finding on either side was counted as positive. In PadChest, where annotations were generated through a combination of manual labeling and NLP, only the subset of labels overlapping with the target label system was retained and binarized accordingly. Finally, whenever available, the “no finding” label was preserved as a separate category to indicate a completely normal radiograph without any imaging abnormality, and not merely the absence of the labels considered in this study.

Images were supplied in mixed formats depending on the dataset. ChestX-ray14, PadChest, CheXpert, and MIMIC-CXR were already available as PNG or JPG files, whereas Pedi-CXR, VinDr-CXR, and UKA-CXR were provided in DICOM format and converted to PNG or JPG before analysis. For DICOM images, metadata were checked to ensure correct polarity; if pixel intensities were stored in inverted form, they were re-inverted to maintain consistent orientation. All radiographs were resized to 224 × 224, 512 × 512, or 1024 × 1024 pixels, depending on the experiment. To normalize intensity values, each image was shifted so that the minimum pixel value corresponded to zero, scaled by its maximum pixel value, and clipped to the valid range before conversion to 8-bit grayscale^6^. Contrast was then enhanced by histogram equalization implemented with the OpenCV library^6,32^. These steps yielded a uniform preprocessing pipeline across datasets, with strict separation of training, validation, and test cohorts wherever applicable.

### Experimental design

To ensure consistency across experiments, we applied a unified preprocessing workflow to all datasets and kept the train, validation, and test partitions fixed throughout the study. The primary benchmark was deliberately designed around patient-wise within-dataset splits and not around dataset-level holdout splits, because its purpose was to compare initialization strategies, backbone families, and input resolutions under otherwise identical conditions. Under a dataset-level holdout design, the difference between initializations and the shift between cohorts would enter the same estimate, and the seven cohorts differ in label provenance, projection mix, clinical context, and disease spectrum. Such a design would also require a label space common to all cohorts, whereas the label sets range from 3 to 17 labels per dataset and only two labels are common to all seven, so most of the per-label detail the benchmark relies on would be lost. Patient-wise splitting removes patient-level leakage, but scanner characteristics, acquisition protocols, patient demographics, and label-generation procedures remain shared between the training and test partitions of a given cohort. The main benchmark should therefore be read as a measure of patient-wise within-dataset generalization, complemented by the targeted external validation experiment described below, and not as a strict assessment of dataset-level generalization.

### Pretrained initialization strategies and backbone architectures

This study was designed as a controlled transfer-learning benchmark and not as a medical-domain pretraining study. Accordingly, DINOv2^20^ and DINOv3^22^ were not retrained on chest radiographs; instead, we used the publicly released pretrained checkpoints directly as initialization for downstream chest radiograph classification. Relative to DINOv2, DINOv3 extends the same general self-supervised framework by introducing Gram-anchored refinement to stabilize dense patch representations during extended training and explicit high-resolution adaptation to support transfer to larger input sizes^22^. These properties motivated our evaluation across resolutions, architectures, and adaptation regimes.

The main benchmark compared three initialization strategies: supervised ImageNet-21K^7^, self-supervised DINOv2, and self-supervised DINOv3. Two backbone families were evaluated: the Vision Transformer base model (ViT-B/16, approximately 86 million parameters) and ConvNeXt-B (approximately 89 million parameters). Because a matching public ConvNeXt-B DINOv2 checkpoint was not available, DINOv2 experiments were performed only with ViT-B/16. The full main benchmark therefore comprised five full fine-tuning configurations: ViT-B/16 initialized from ImageNet, DINOv2, or DINOv3, and ConvNeXt-B initialized from ImageNet or DINOv3.

### Main full fine-tuning benchmark

Universal experiments were performed at 224 × 224 and 512 × 512 pixels across all seven datasets. To probe whether the 512 × 512 advantage extended further, we additionally evaluated 1024 × 1024 pixels on three representative cohorts spanning pediatric and adult settings, namely Pedi-CXR, ChestX-ray14, and MIMIC-CXR.

All full fine-tuning experiments were optimized with Adam using a learning rate of 10^−5^ and no weight decay. This learning rate was chosen based on empirical testing of multiple candidate values in the present study, where it provided the most stable convergence across datasets and model configurations, and it is also consistent with the range used in our prior work on chest radiograph classification^21,32–34,36,37^. Across all experiments, data augmentation consisted of random horizontal flips and random rotations up to 7°. The loss function was binary weighted cross-entropy, with class weights set inversely proportional to the frequency of each label in the training set^38^. All training was performed in full 32-bit floating-point precision on a single NVIDIA L40S GPU with 48 GB VRAM and Intel Xeon Silver 4310 CPUs. For full fine-tuning, batch size was 128 at 224 × 224, 64 at 512 × 512, and 16 at 1024 × 1024. To preserve comparability across initialization strategies, backbones, and resolutions, all main benchmark models were trained under this shared optimization protocol instead of under separate per-configuration hyperparameter searches.

### Frozen-feature probing with DINOv3-7B

To test whether a much larger frozen foundation encoder could substitute for downstream adaptation, we additionally evaluated frozen representations from the DINOv3-7B model. In this setting, the backbone remained fully frozen and only a compact multilayer classification head, referred to as DINO-Net, was optimized. DINO-Net consisted of layer normalization^39^ applied to the 4096-dimensional backbone features, a linear projection to a 512-dimensional embedding, a Gaussian error linear unit (GELU) activation^40^, a second layer normalization, dropout with p = 0.3, and a final linear mapping to the target label space. This head added approximately 2.1 million trainable parameters. Frozen-feature experiments were performed on all seven datasets at 224 × 224 and 512 × 512 pixels. Only the DINO-Net classifier was optimized, using AdamW with a learning rate of 10^−4^ and no weight decay.

### Parameter-efficient adaptation with LoRA

To test whether the observed backbone ranking depended on the use of full end-to-end fine-tuning, we performed a dedicated parameter-efficient adaptation analysis using low-rank adaptation (LoRA)^41^. These experiments were conducted at 512 × 512 pixels on three adult datasets, namely ChestX-ray14, CheXpert, and MIMIC-CXR. We evaluated the same five backbone-initialization combinations as in the full fine-tuning benchmark: ViT-B/16 with ImageNet, DINOv2, or DINOv3 initialization, and ConvNeXt-B with ImageNet or DINOv3 initialization.

For ViT-B/16, LoRA adapters were applied to the attention projection layers, specifically query and value projections or their architecture-equivalent implementation. For ConvNeXt-B, adapters were applied to the pointwise projection layers within the ConvNeXt blocks, again using the corresponding implementation-specific module names. In all LoRA experiments, the classification head remained trainable. LoRA hyperparameters were fixed to rank 16, alpha 32, and dropout 0.05. Training used AdamW^42^ with learning rate 10^−4^ and batch size 16.

### Synthetic label-noise experiment

To test whether gains from DINOv3 on weakly labeled adult datasets could be explained simply by improved tolerance to noisy supervision, we performed a controlled synthetic label-noise experiment on VinDr-CXR using ViT-B/16 at 512 × 512 pixels. This analysis compared the three ViT-B/16 initialization strategies, namely ImageNet, DINOv2, and DINOv3.

Noise was injected only into the training split, while the test set remained unchanged. Corruption was applied at predefined noise levels of 10%, 20%, 30%, and 40%, with the clean training labels serving as the 0% reference. Noise was introduced only for the 10 abnormal labels in the VinDr-CXR setup, excluding the “No finding” label. We used an asymmetric corruption scheme intended to better approximate weak-label errors: positive labels were flipped to negative at the specified noise rate, whereas negative labels were flipped to positive at one quarter of that rate. After corruption of the abnormal labels, the “No finding” label was recomputed on the training set so that it remained mutually consistent with the pathology labels. Test labels were never modified.

### External validation across institutions

To determine whether the main benchmark findings extended beyond within-dataset train-test splits, we performed an external validation experiment using a harmonized seven-label setting comprising cardiomegaly, pleural effusion, pneumonia, atelectasis, no finding, consolidation, and pneumothorax. Models were trained on MIMIC-CXR and evaluated both on the internal MIMIC-CXR test set and on the fully held-out external datasets ChestX-ray14 and CheXpert. External validation was performed at 224 × 224 and 512 × 512 pixels for the same five backbone-initialization combinations used in the main benchmark.

### Computational efficiency analysis

To place performance differences in a practical context, computational efficiency was assessed for all full fine-tuning experiments by measuring both per-epoch training time and total wall-clock time to convergence on the same hardware platform, namely a single NVIDIA L40S GPU with 48 GB VRAM and Intel Xeon Silver 4310 CPUs, using full 32-bit floating point precision throughout. This analysis was intended as a practical efficiency comparison within the present benchmark and not as a formal systems-level profiling study; accordingly, it did not include inference latency, GPU memory traces, or FLOPs.

Per-epoch training time was recorded for each evaluated full fine-tuning configuration, defined by dataset, initialization, backbone, and image resolution. Across the universal 224 ×  224 and 512 × 512 experiments, this yielded measurements for all seven datasets, three initialization strategies for ViT-B/16, and two initialization strategies for ConvNeXt-B. For the targeted 1024 × 1024 setting, measurements were obtained only for the three representative cohorts evaluated at that resolution, namely Pedi-CXR, ChestX-ray14, and MIMIC-CXR. For each configuration, we also recorded the number of epochs required to convergence, allowing computation of total wall-clock training time.

Relative computational cost was then summarized as ratios across resolution transitions, particularly for 224 × 224 to 512 × 512 and 512 × 512 to 1024 × 1024. These ratios were aggregated across the relevant sets of configurations and reported descriptively using means, standard deviations, medians, and ranges. This analysis allowed us to relate performance changes directly to measured training cost under a fixed implementation and hardware setting.

### Evaluation and statistical analysis

The primary evaluation metric was the area under the receiver operating characteristic curve (AUROC), which provides a threshold-independent measure of discrimination in multilabel classification. For the main benchmark, we summarized performance within each dataset using the mean AUROC across all labels. Unless otherwise specified, all performance values reported in this study are mean AUROC across labels and are given as mean ± standard deviation [95% confidence interval] in percent. Per-label AUROC values are provided in the supplementary information. Accuracy, sensitivity, and specificity were computed as complementary metrics, with operating thresholds chosen according to Youden’s criterion^43^, i.e., the cut-off maximizing the difference between true-positive and false-positive rates. For follow-up analyses involving class imbalance, including parameter-efficient adaptation, synthetic label corruption, and external validation, we additionally report precision-recall area under the curve (PR-AUC) and mean average precision (mAP).

Statistical analysis was performed using Python 3.9 with NumPy 1.22, SciPy 1.10, and scikit-learn 1.2. Bootstrapping with 1000 redraws was used to estimate means, standard deviations, and 95% confidence intervals (CI)^21,44^. For within-dataset comparisons between initialization strategies, backbones, resolutions, or adaptation regimes, paired^45^ bootstrap testing was performed on AUROC differences using identical resampling across compared models^21,31,36^. Formal cross-resolution testing between 512 × 512 and 1024 × 1024 was performed for all configurations evaluated at both resolutions. To account for multiple comparisons across related groups of tests, *p*-values were adjusted using the Benjamini–Hochberg false discovery rate (FDR) procedure, with statistical significance defined as FDR-adjusted *p* < 0.05^46,47^.

External validation experiments were performed by training on MIMIC-CXR and testing on ChestX-ray14 and CheXpert using a harmonized seven-label setting comprising cardiomegaly, pleural effusion, pneumonia, atelectasis, no finding, consolidation, and pneumothorax. The synthetic label-noise experiment was conducted on VinDr-CXR by progressively corrupting training labels at predefined noise levels while keeping the test set unchanged. Computational efficiency was assessed using measured per-epoch training time and total wall-clock time to convergence for each evaluated configuration on the same hardware platform, and scaling behavior was summarized as relative cost ratios across image resolutions.

## Results

### DINOv3 gains emerge at 512 × 512 pixels in adult chest radiograph cohorts

**Fig. 2:**
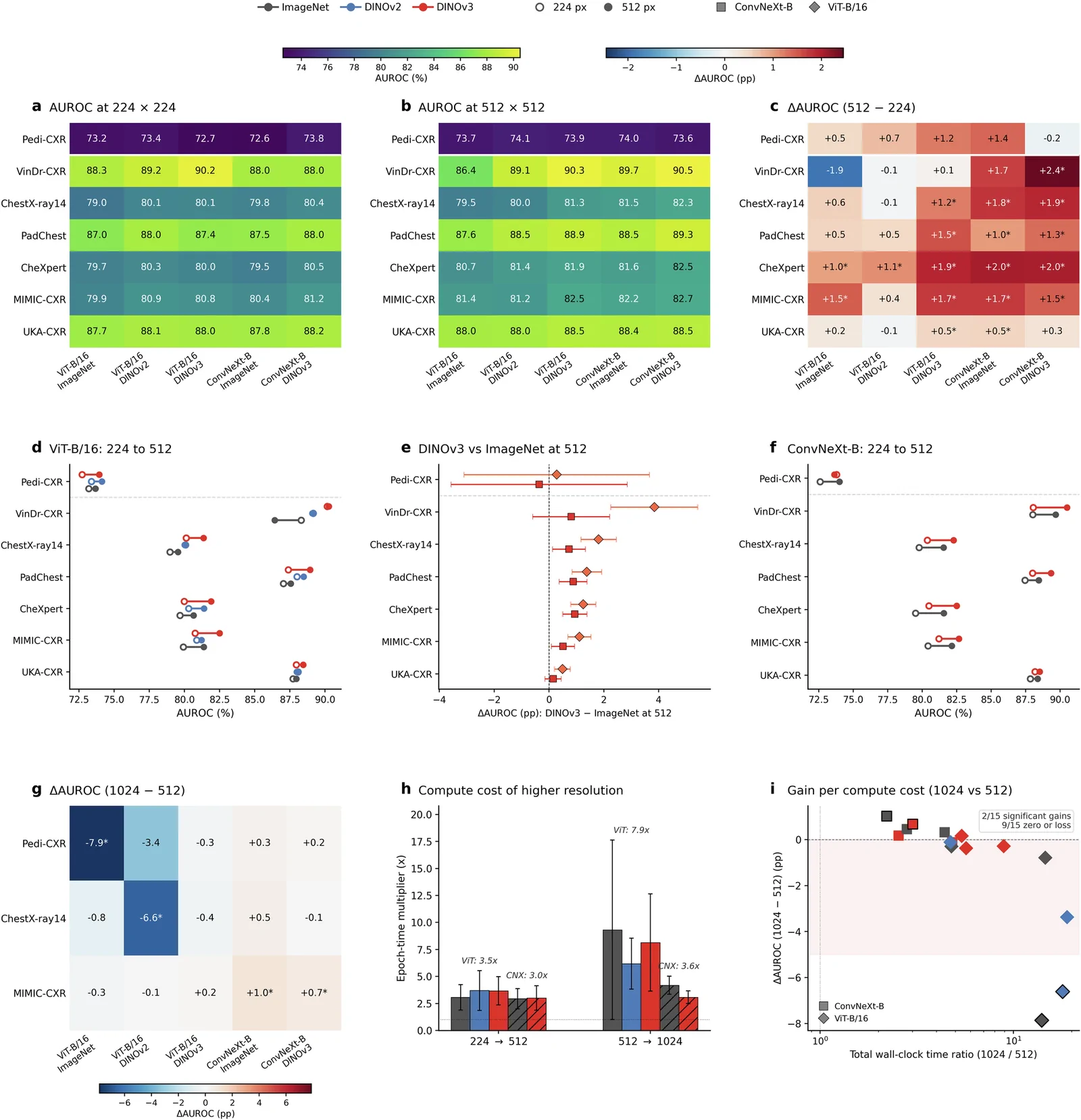
Resolution scaling and computational cost-benefit analysis. All panels cover the seven datasets and the five configurations (ViT-B/16 with ImageNet, DINOv2 or DINOv3, and ConvNeXt-B with ImageNet or DINOv3); **g**, **i** cover only the three datasets evaluated at 1024 × 1024 pixels. In **d**–**f**, **h**, **i**, color denotes the initialization; squares denote ConvNeXt-B and diamonds ViT-B/16; hatching marks ConvNeXt-B in (**h**), and open and filled markers denote 224 × 224 and 512 × 512 pixels. **a**–**c**, **g** Are heatmaps whose cells are colored by the value shown. Every AUROC is the mean over 1000 bootstrap resamples of the relevant test set (*n* = 1397 for Pedi-CXR, 3000 for VinDr-CXR, 25,596 for ChestX-ray14, 22,045 for PadChest, 29,318 for CheXpert, 43,793 for MIMIC-CXR and 40,106 for UKA-CXR). Mean AUROC at 224 × 224 (**a**) and at 512 × 512 (**b**); the horizontal line separates Pedi-CXR from the adult datasets. **c** The change between those two resolutions. The same transition is shown as dumbbells for ViT-B/16 (**d**) and for ConvNeXt-B (**f**). **e** The DINOv3 minus ImageNet difference at 512 × 512; the error bars are an approximate 95% confidence interval of that difference, obtained as the square root of the sum of the squared half-widths of the two models’ bootstrap 95% confidence intervals. **g** The change from 512 × 512 to 1024 × 1024. **h** Epoch-time multiplier of each resolution transition, one bar per configuration; each bar is the arithmetic mean of the per-epoch time ratio across the contributing datasets, *n* = 7 for 224 × 224 to 512 × 512 and *n* = 3 for 512 × 512 to 1024 × 1024, and the error bars are one standard deviation about that mean. **i** AUROC change against the total wall-clock time ratio on a logarithmic x-axis; the shaded region marks zero or negative change at higher cost. Stars in (**c**, **g**), and thick marker edges in (**i**), mark non-overlapping 95% confidence intervals. **a**–**c** Show 35 cells each, and **g** shows 15; **d** shows 21 dumbbells, **f** 14, **e** 14 estimates, and **i** 15 points. AUROC, area under the receiver operating characteristic curve.

Across the six adult cohorts, the relative behavior of ImageNet, DINOv2, and DINOv3 depended strongly on input resolution (Fig. 2, Table 2, Supplementary Table 1, and Supplementary Figs. 1–7). At 224 × 224 pixels, DINOv3 did not consistently outperform DINOv2. In the ViT-B/16 setting, DINOv3 was numerically higher in AUROC only in VinDr-CXR (90.2 ± 0.6 vs. 89.2 ± 0.7), whereas DINOv2 remained equal or higher in the other adult datasets, including PadChest (88.0 ± 0.2 vs. 87.4 ± 0.2; *p* = 0.003), CheXpert (80.3 ± 0.2 vs. 80.0 ± 0.2; *p* = 0.003), and UKA-CXR (88.1 ± 0.1 vs. 88.0 ± 0.1; *p* = 0.008). Thus, at standard resolution, DINOv3 improved over ImageNet in all adult ViT-B/16 cohorts (*p* ≤ 0.012), but did not yet establish a clear advantage over DINOv2.

**Table 2:** Core benchmark performance across datasets, initialization strategies, backbone families, and input resolutions

Configuration	Pedi-CXR	VinDr-CXR	ChestX-ray14	PadChest	CheXpert	MIMIC-CXR	UKA-CXR
ViT-B ImageNet 224	73.2 ± 1.2 [70.9, 75.5]	88.3 ± 0.6 [87.0, 89.5]	79.0 ± 0.2 [78.6, 79.4]	87.0 ± 0.2 [86.7, 87.4]	79.7 ± 0.2 [79.4, 80.0]	79.9 ± 0.2 [79.6, 80.2]	87.7 ± 0.1 [87.5, 87.9]
ViT-B ImageNet 224 *P*-value	0.31	0.006	0.006	0.012	0.006	0.006	0.006
ViT-B DINOv2 224	73.4 ± 1.2 [71.1, 75.6]	89.2 ± 0.7 [87.5, 90.5]	80.1 ± 0.2 [79.6, 80.5]	88.0 ± 0.2 [87.6, 88.4]	80.3 ± 0.2 [80.0, 80.6]	80.9 ± 0.2 [80.6, 81.2]	88.1 ± 0.1 [87.9, 88.3]
ViT-B DINOv2 224 *P*-value	0.61	0.11	0.006	0.006	0.006	0.006	0.006
ViT-B DINOv3 224	72.7 ± 1.2 [70.2, 75.1]	90.2 ± 0.6 [89.0, 91.2]	80.1 ± 0.2 [79.7, 80.6]	87.4 ± 0.2 [87.0, 87.8]	80.0 ± 0.2 [79.7, 80.3]	80.8 ± 0.1 [80.5, 81.0]	88.0 ± 0.1 [87.8, 88.2]
ConvNeXt-B ImageNet 224	72.6 ± 1.2 [70.2, 75.0]	88.0 ± 0.6 [86.8, 89.1]	79.8 ± 0.2 [79.4, 80.2]	87.5 ± 0.2 [87.0, 87.8]	79.5 ± 0.2 [79.2, 79.8]	80.4 ± 0.1 [80.1, 80.7]	87.8 ± 0.1 [87.6, 88.0]
ConvNeXt-B ImageNet 224 *P*-value	0.08	0.45	0.001	0.001	0.001	0.001	0.001
ConvNeXt-B DINOv3 224	73.8 ± 1.1 [71.6, 76.0]	88.0 ± 0.6 [87.0, 89.1]	80.4 ± 0.2 [80.0, 80.8]	88.0 ± 0.2 [87.6, 88.3]	80.5 ± 0.2 [80.2, 80.8]	81.2 ± 0.1 [80.9, 81.5]	88.2 ± 0.1 [88.0, 88.4]
ViT-B ImageNet 512	73.7 ± 1.2 [71.1, 76.0]	86.4 ± 0.7 [85.1, 87.6]	79.5 ± 0.2 [79.1, 80.0]	87.6 ± 0.2 [87.2, 88.0]	80.7 ± 0.2 [80.3, 81.0]	81.4 ± 0.1 [81.1, 81.7]	88.0 ± 0.1 [87.8, 88.2]
ViT-B ImageNet 512 *P*-value	0.37	0.006	0.006	0.006	0.006	0.006	0.006
ViT-B DINOv2 512	74.1 ± 1.2 [72.0, 76.4]	89.1 ± 0.5 [88.0, 90.1]	80.0 ± 0.2 [79.6, 80.4]	88.5 ± 0.2 [88.1, 88.8]	81.4 ± 0.2 [81.1, 81.7]	81.2 ± 0.1 [80.9, 81.5]	88.0 ± 0.1 [87.8, 88.2]
ViT-B DINOv2 512 *P*-value	0.32	0.006	0.006	0.006	0.006	0.044	0.26
ViT-B DINOv3 512	73.9 ± 1.2 [71.6, 76.3]	90.3 ± 0.5 [89.3, 91.2]	81.3 ± 0.2 [80.9, 81.8]	88.9 ± 0.2 [88.6, 89.3]	81.9 ± 0.2 [81.6, 82.2]	82.5 ± 0.1 [82.2, 82.8]	88.5 ± 0.1 [88.2, 88.7]
ConvNeXt-B ImageNet 512	74.0 ± 1.1 [71.8, 76.2]	89.7 ± 0.5 [88.6, 90.7]	81.5 ± 0.2 [81.1, 81.9]	88.5 ± 0.2 [88.0, 88.8]	81.6 ± 0.2 [81.2, 81.9]	82.2 ± 0.1 [81.9, 82.4]	88.4 ± 0.1 [88.2, 88.6]
ConvNeXt-B ImageNet 512 *P*-value	0.66	0.013	0.001	0.001	0.001	0.001	0.001
ConvNeXt-B DINOv3 512	73.6 ± 1.2 [71.2, 75.9]	90.5 ± 0.5 [89.5, 91.5]	82.3 ± 0.2 [81.8, 82.7]	89.3 ± 0.2 [89.0, 89.7]	82.5 ± 0.1 [82.2, 82.8]	82.7 ± 0.2 [82.3, 83.0]	88.5 ± 0.1 [88.3, 88.7]
Best-performing configuration	ViT-B DINOv2 512	ConvNeXt-B DINOv3 512	ConvNeXt-B DINOv3 512	ConvNeXt-B DINOv3 512	ConvNeXt-B DINOv3 512	ConvNeXt-B DINOv3 512	ConvNeXt-B DINOv3 512

Average area under the receiver operating characteristic curve (AUROC) derived from 1000 bootstrap resamples for full fine-tuning of ViT-B/16 and ConvNeXt-B backbones across the universal input resolutions of 224 × 224 and 512 × 512 pixels for all seven datasets: Pedi-CXR (training *n* = 6955; test *n* = 1397), VinDr-CXR (training *n* = 15,000; test *n* = 3000), ChestX-ray14 (training *n* = 77,870; validation *n* = 8654; test *n* = 25,596), PadChest (training *n* = 79,697; validation *n* = 8783; test *n* = 22,045), CheXpert (training *n* = 115,449; validation *n* = 13,098; test *n* = 29,318), MIMIC-CXR (training *n* = 153,255; validation *n* = 18,139; test *n* = 43,793), and UKA-CXR (training *n* = 137,902; validation *n* = 15,353; test *n* = 40,106). Results are reported for models initialized from ImageNet, DINOv2, and DINOv3. For each evaluated configuration, results are reported as mean ± standard deviation with 95% confidence intervals (CIs).

At 512 × 512 pixels, this pattern changed. For ViT-B/16, DINOv3 outperformed DINOv2 in all six adult cohorts, including VinDr-CXR (90.3 ± 0.5 vs. 89.1 ± 0.5; *p* = 0.019), ChestX-ray14 (81.3 ± 0.2 vs. 80.0 ± 0.2; *p* = 0.006), PadChest (88.9 ± 0.2 vs. 88.5 ± 0.2; p = 0.006), CheXpert (81.9 ± 0.2 vs. 81.4 ± 0.2; *p* = 0.006), MIMIC-CXR (82.5 ± 0.1 vs. 81.2 ± 0.1; *p* = 0.006), and UKA-CXR (88.5 ± 0.1 vs. 88.0 ± 0.1; *p* = 0.006). The strongest overall results were obtained with ConvNeXt-B, where DINOv3 at 512 × 512 gave the highest mean AUROC in all six adult datasets, reaching 90.5 ± 0.5 in VinDr-CXR, 82.3 ± 0.2 in ChestX-ray14, 89.3 ± 0.2 in PadChest, 82.5 ± 0.1 in CheXpert, 82.7 ± 0.2 in MIMIC-CXR, and 88.5 ± 0.1 in UKA-CXR. Relative to ConvNeXt-B with ImageNet initialization, these gains were significant in all six adult cohorts (*p* ≤ 0.013).

The key result was therefore not simply that higher resolution improved accuracy, but that it changed which initialization was preferable. Across adult datasets, the most consistent 224-to-512 gains were concentrated in DINOv3-initialized models, particularly with ConvNeXt-B (Fig. 2c–f). This resolution dependence was also reflected at the finding-group level: the largest average gains were observed for small focal lesions and boundary-dependent findings, whereas large-structure findings changed little between 224 × 224 and 512 × 512 pixels (Supplementary Table 2 and Supplementary Fig. 8). Representative example radiographs for findings that showed the largest apparent resolution sensitivity are provided in Supplementary Fig. 8. In contrast, the pediatric cohort did not follow this pattern. Pedi-CXR showed minimal separation between configurations at either resolution, with no significant advantage for DINOv3 over ImageNet or DINOv2 at 224 × 224 or 512 × 512 (all *p* ≥ 0.31). Because this behavior differed qualitatively from the adult cohorts, we analyzed the pediatric dataset separately in the next section.

### The pediatric cohort shows a distinct transfer pattern that does not mirror adult resolution scaling

**Fig. 3:**
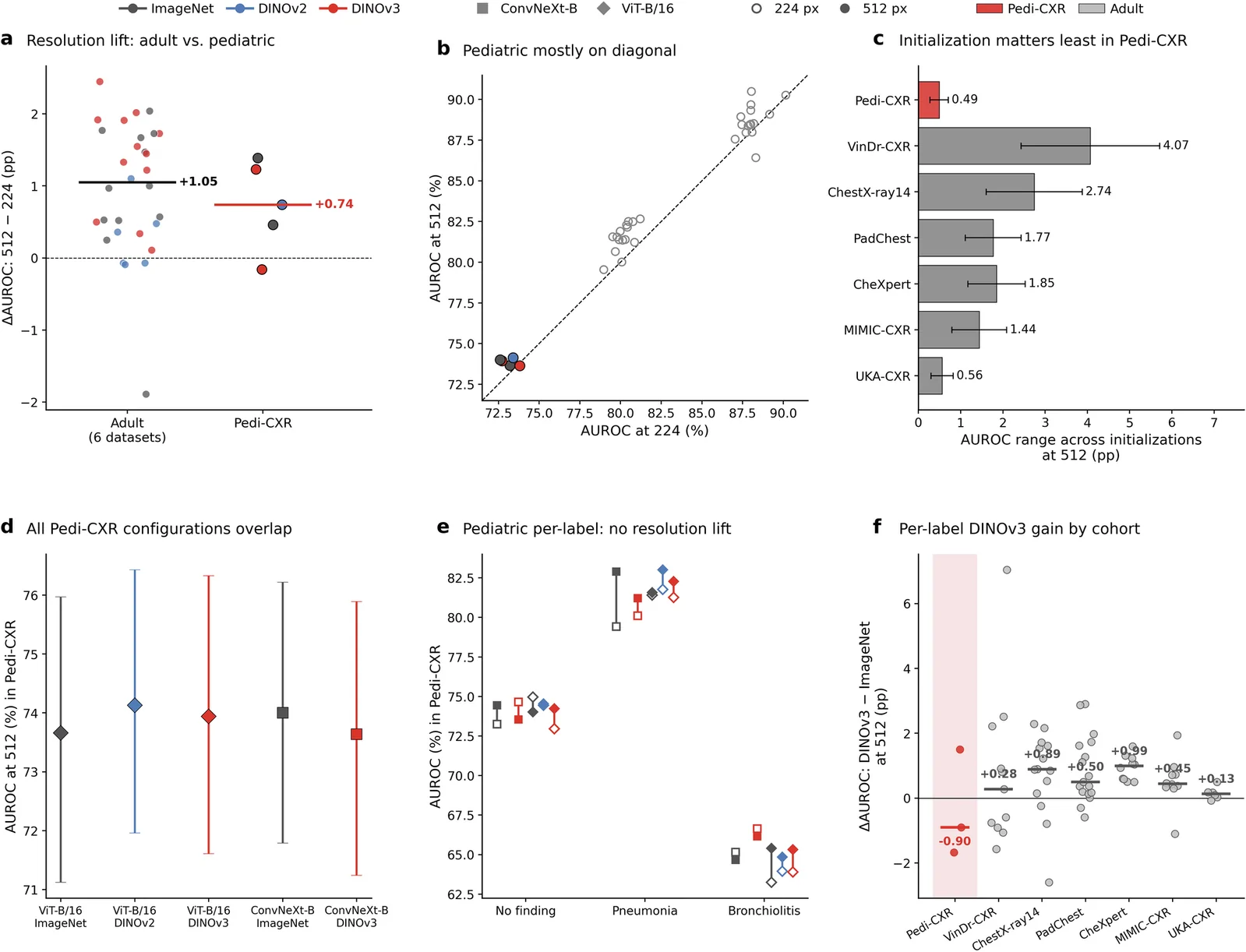
Transfer behavior of the pediatric cohort compared with the adult cohorts. All panels cover the seven datasets and the five configurations (ViT-B/16 with ImageNet, DINOv2 or DINOv3, and ConvNeXt-B with ImageNet or DINOv3), which give 30 adult and 5 pediatric configurations. Squares denote ConvNeXt-B and diamonds ViT-B/16; open and filled markers denote 224 × 224 and 512 × 512 pixels, color denotes the initialization, the adult configurations of **b** are shown in gray, and in **c**, **f**, red marks Pedi-CXR and gray the adult cohorts. Every AUROC is the mean over 1000 bootstrap resamples of the relevant test set (*n* = 1397 for Pedi-CXR, 3000 for VinDr-CXR, 25,596 for ChestX-ray14, 22,045 for PadChest, 29,318 for CheXpert, 43,793 for MIMIC-CXR and 40,106 for UKA-CXR). **a** AUROC change from 224 × 224 to 512 × 512 for every configuration, split into the six adult cohorts and Pedi-CXR, with horizontal bars marking the group median. **b** AUROC at 224 × 224 against AUROC at 512 × 512 for the same 35 configurations, with the dashed line denoting equality. **c** Range of the mean AUROC across the five configurations at 512 × 512, one bar per dataset; the bar length is the maximum minus the minimum, and the error bars are one standard deviation of those five values. **d** Mean AUROC at 512 × 512 for the five Pedi-CXR configurations, with the 95% confidence interval taken as the 2.5th and 97.5th percentiles of the bootstrap distribution. **e** The 224 × 224 to 512 × 512 shift for each of the three pediatric labels, 15 points in total. **f** Per-label difference between DINOv3 and ImageNet at 512 × 512 with ConvNeXt-B, one point per label and 71 labels in total, with horizontal bars marking the median per dataset. AUROC, area under the receiver operating characteristic curve.

Pedi-CXR was not simply the weakest-performing dataset, but the only cohort that failed to follow the adult resolution-scaling pattern (Table 2). Whereas adult configurations showed a consistent positive lift when moving from 224 × 224 to 512 × 512 pixels, pediatric configurations clustered around zero, with a median gain of only +0.74 percentage points compared with +1.05 percentage points across adult cohorts (Fig. 3a). This difference was also evident when plotting performance at 224 × 224 vs. 512 × 512 pixels directly: adult configurations lay predominantly above the identity line, whereas the pediatric points remained close to it (Fig. 3b).

At the dataset level, performance differences between initialization and backbone choices were minimal in Pedi-CXR. At 512 × 512 pixels, all five evaluated configurations fell within a narrow AUROC range of 0.49 percentage points, far smaller than in any adult cohort (Fig. 3c). Their uncertainty intervals also overlapped broadly (Fig. 3d), and none of the pairwise comparisons between DINOv3 and ImageNet or DINOv2 reached significance at either 224 ×  224 or 512 × 512 pixels (all *p* ≥ 0.31; Table 2 and Supplementary Table 1). Even the nominally highest pediatric configuration, ViT-B/16 with DINOv2 at 512 × 512, reached only 74.1 ± 1.2, while ConvNeXt-B with DINOv3 reached 73.6 ± 1.2.

This absence of a pediatric resolution effect also held at the label level. None of the three Pedi-CXR labels showed a consistent upward shift from 224 to 512 pixels across configurations (Fig. 3e). Moreover, when comparing DINOv3 with ImageNet at 512 × 512 using ConvNeXt-B, Pedi-CXR was the only cohort with a negative median label-level effect, whereas all adult datasets showed positive medians (Fig. 3f). Thus, the adult result that DINOv3 benefits from higher resolution did not transfer to the pediatric setting. These findings indicate that the adult DINOv3-at-512 pattern should not be generalized automatically to pediatric chest radiography. In this cohort, increasing resolution and changing initialization or backbone had little measurable impact, suggesting that the determinants of transfer performance differ materially between pediatric and adult chest X-ray analysis.

### Scaling beyond 512 × 512 yields limited benefit despite sharply increased computational cost

In the three cohorts evaluated at 1024 × 1024 pixels, additional scaling rarely improved performance (Fig. 2g–i, Table 3, and Supplementary Table 3). Across the 15 evaluated dataset-configuration pairs, only two yielded significant gains, both in MIMIC-CXR with ConvNeXt-B: ImageNet increased from 82.2 ± 0.1 to 83.2 ± 0.1 (ΔAUROC = + 1.0 ± 0.2) and DINOv3 from 82.7 ± 0.2 to 83.3 ± 0.1 (ΔAUROC = + 0.7 ± 0.2). All remaining comparisons were neutral or negative, including clear degradations for several ViT-B/16 settings, such as Pedi-CXR with ImageNet (73.7 ± 1.2 to 65.8 ± 1.4; ΔAUROC = − 7.9 ± 1.8) and ChestX-ray14 with DINOv2 (80.0 ± 0.2 to 73.4 ± 0.2; ΔAUROC = − 6.6 ± 0.3).

**Table 3:** Targeted high-resolution comparison and computational efficiency

Configuration	Pedi-CXR	ChestX-ray14	MIMIC-CXR
ViT-B ImageNet: AUROC at 512 × 512	73.7 ± 1.2 [71.1, 76.0] (*p* = 0.37)	79.5 ± 0.2 [79.1, 80.0] (*p* = 0.006)	81.4 ± 0.1 [81.1, 81.7] (*p* = 0.006)
ViT-B ImageNet: AUROC at 1024 × 1024	65.8 ± 1.4 [63.0, 68.4] (*p* = 0.001)	78.8 ± 0.2 [78.3, 79.2] (*p* = 0.002)	81.1 ± 0.2 [80.8, 81.4] (*p* = 0.002)
ViT-B ImageNet: ΔAUROC (1024–512)	−7.9 ± 1.8 [−11.5, −4.2]	−0.8 ± 0.3 [−1.4, −0.1]	−0.3 ± 0.2 [−0.7, 0.1]
ViT-B ImageNet: total training-time ratio (1024/512)	11.1 ± 5.5	11.1 ± 5.5	11.1 ± 5.5
ViT-B DINOv2: AUROC at 512 × 512	74.1 ± 1.2 [72.0, 76.4] (*p* = 0.54)	80.0 ± 0.2 [79.6, 80.4] (*p* = 0.006)	81.2 ± 0.1 [80.9, 81.5] (*p* = 0.006)
ViT-B DINOv2: AUROC at 1024 × 1024	70.8 ± 1.2 [68.3, 73.2] (*p* = 0.001)	73.4 ± 0.2 [72.9, 73.8] (*p* = 0.002)	81.1 ± 0.1 [80.8, 81.4] (*p* = 0.002)
ViT-B DINOv2: ΔAUROC (1024–512)	−3.4 ± 1.7 [−6.7, −0.0]	−6.6 ± 0.3 [−7.2, −6.0]	−0.1 ± 0.2 [−0.5, 0.3]
ViT-B DINOv2: total training-time ratio (1024/512)	13.9 ± 7.9	13.9 ± 7.9	13.9 ± 7.9
ViT-B DINOv3: AUROC at 512 × 512	73.9 ± 1.2 [71.6, 76.3]	81.3 ± 0.2 [80.9, 81.8]	82.5 ± 0.1 [82.2, 82.8]
ViT-B DINOv3: AUROC at 1024 × 1024	73.7 ± 1.2 [71.4, 75.9]	81.0 ± 0.2 [80.5, 81.4]	82.7 ± 0.1 [82.4, 83.0]
ViT-B DINOv3: ΔAUROC (1024–512)	−0.3 ± 1.7 [−3.5, 3.0]	−0.4 ± 0.3 [−1.0, 0.3]	0.2 ± 0.2 [−0.2, 0.6]
ViT-B DINOv3: total training-time ratio (1024/512)	6.7 ± 1.9	6.7 ± 1.9	6.7 ± 1.9
ConvNeXt-B ImageNet: AUROC at 512 × 512	74.0 ± 1.1 [71.8, 76.2] (*p* = 0.66)	81.5 ± 0.2 [81.1, 81.9] (*p* = 0.001)	82.2 ± 0.1 [81.9, 82.4] (*p* = 0.001)
ConvNeXt-B ImageNet: AUROC at 1024 × 1024	74.3 ± 1.2 [72.1, 76.7] (*p* = 0.76)	82.0 ± 0.2 [81.6, 82.4] (*p* = 0.002)	83.2 ± 0.1 [82.9, 83.5] (*p* = 0.038)
ConvNeXt-B ImageNet: ΔAUROC (1024–512)	0.3 ± 1.6 [−2.9, 3.5]	0.5 ± 0.3 [−0.1, 1.0]	1.0 ± 0.2 [0.6, 1.4]
ConvNeXt-B ImageNet: total training-time ratio (1024/512)	3.1 ± 1.1	3.1 ± 1.1	3.1 ± 1.1
ConvNeXt-B DINOv3: AUROC at 512 × 512	73.6 ± 1.2 [71.2, 75.9]	82.3 ± 0.2 [81.8, 82.7]	82.7 ± 0.2 [82.3, 83.0]
ConvNeXt-B DINOv3: AUROC at 1024 × 1024	73.8 ± 1.2 [71.5, 76.1]	82.2 ± 0.2 [81.7, 82.6]	83.3 ± 0.1 [83.1, 83.6]
ConvNeXt-B DINOv3: ΔAUROC (1024– 512)	0.2 ± 1.7 [−3.1, 3.5]	−0.1 ± 0.3 [−0.8, 0.5]	0.7 ± 0.2 [0.3, 1.1]
ConvNeXt-B DINOv3: total training-time ratio (1024/512)	3.5 ± 1.2	3.5 ± 1.2	3.5 ± 1.2

Focused 512 × 512 vs. 1024 × 1024 comparison for the three representative datasets evaluated at 1024 × 1024 resolution, namely Pedi-CXR (training *n* = 6955; test *n* = 1397), ChestX-ray14 (training *n* = 77,870; validation *n* = 8654; test *n* = 25,596), and MIMIC-CXR (training *n* = 153,255; validation *n* = 18,139; test *n* = 43,793). For each model configuration, the table reports AUROC at 512 × 512 and 1024 × 1024 as mean ± SD [95% CI]. For non-DINOv3 initialization strategies, the corresponding adjusted pairwise bootstrap *p*-value vs. DINOv3 at the same dataset and resolution is appended to the absolute AUROC estimate. The table further reports the absolute cross-resolution change in AUROC (ΔAUROC = AUROC-1024–AUROC-512) as mean ± SD [95% CI], and the relative total training-time ratio (1024/512). Pairwise bootstrap *p*-values were computed as two-sided *p*-values from paired bootstrap tests using 1000 resampled AUROC pairs per model, with identical resampling across initialization strategies to ensure fair comparison. The total training-time ratio is reported once per backbone-initialization configuration as mean ± SD across the three cohorts evaluated at both resolutions, and not separately for each dataset.

The overall pattern was therefore not one of continued monotonic improvement with increasing resolution. In Pedi-CXR, 1024 × 1024 provided no measurable benefit for any configuration. In ChestX-ray14, performance changes were small for ConvNeXt-B and negative for all ViT-B/16 initializations. Only MIMIC-CXR showed a modest positive shift, and even there the gains were confined to ConvNeXt-B and remained well below one AUROC point for DINOv3 (Fig. 2g and Table 3). Thus, while DINOv3 retained its strong relative standing at 1024 × 1024 wherever tested against alternative initializations at the same resolution, scaling beyond 512 × 512 did not produce a consistent cross-dataset accuracy benefit.

These limited returns contrasted sharply with the computational cost of 1024-pixel training. Across all full fine-tuning experiments, moving from 512 × 512 to 1024 × 1024 increased per-epoch training time by 6.2 ± 4.4-fold overall, with a larger penalty for ViT-B/16 than for ConvNeXt-B (7.9 ± 5.1-fold vs. 3.6 ± 0.9-fold; Supplementary Table 3). The effect on end-to-end training time was even more pronounced: the median total wall-clock ratio for 1024 × 1024 vs. 512 × 512 was 4.8 overall, but 8.9 for ViT-B/16 and 2.9 for ConvNeXt-B. In the targeted 1024 × 1024 experiments, this translated to mean total training-time ratios of 11.1 ± 5.5 for ViT-B/ImageNet, 13.9 ± 7.9 for ViT-B/DINOv2, 6.7 ± 1.9 for ViT-B/DINOv3, 3.1 ± 1.1 for ConvNeXt-B/ImageNet, and 3.5 ± 1.2 for ConvNeXt-B/DINOv3 (Table 3 and Fig. 2h, i). Under the present training setup, 1024 × 1024 training rarely improves classification performance enough to justify its substantially higher cost. The practical operating point of the benchmark therefore remained at 512 × 512 pixels, where DINOv3 already achieved its most reproducible gains across adult cohorts without incurring the marked efficiency penalty of further resolution scaling.

### ConvNeXt-B remains superior to ViT-B/16 under both full and parameter-efficient adaptation

**Fig. 4:**
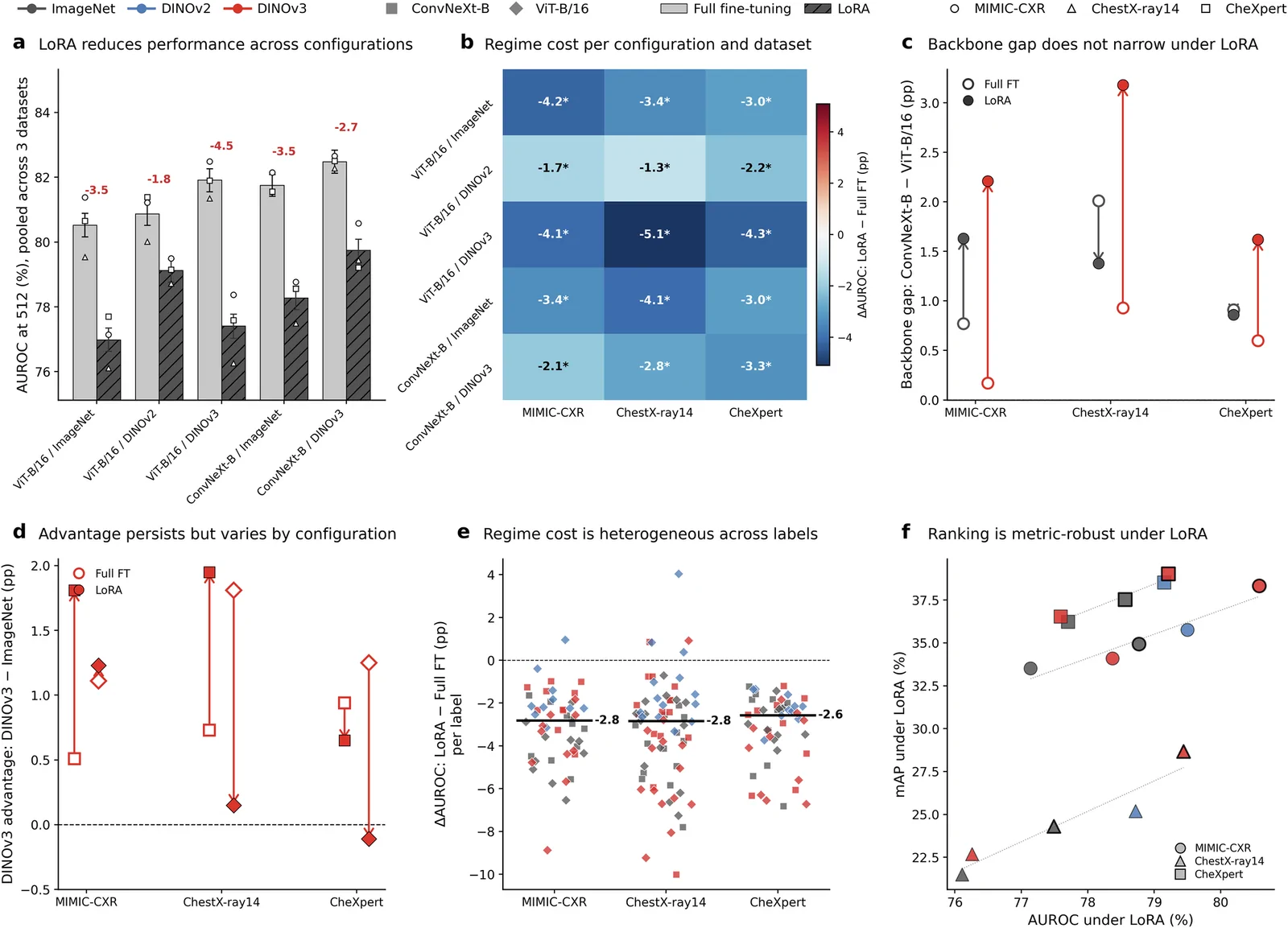
Full fine-tuning compared with parameter-efficient fine-tuning by low-rank adaptation (LoRA). All panels use the three adult datasets on which LoRA was evaluated, at 512 × 512 pixels, and the five configurations (ViT-B/16 with ImageNet, DINOv2 or DINOv3, and ConvNeXt-B with ImageNet or DINOv3). Color denotes the initialization in (**c**–**f**); open and filled markers denote full fine-tuning and LoRA in (**c**, **d**), and the light and dark hatched bars of **a** denote those same two regimes. Marker shape denotes the dataset in (**a**, **f**) (circles MIMIC-CXR, triangles ChestX-ray14, squares CheXpert) and the backbone in (**d**, **e**) (squares ConvNeXt-B, diamonds ViT-B/16). Every AUROC and mAP is the mean over 1000 bootstrap resamples of the relevant test set (*n* = 43,793 for MIMIC-CXR, 25,596 for ChestX-ray14 and 29,318 for CheXpert). **a** Mean AUROC of each configuration under both regimes; each bar is the mean across the three datasets, the error bars are the corresponding 95% confidence intervals averaged over those datasets, and the overlaid markers are the three individual dataset values. **b** AUROC change from full fine-tuning to LoRA, one cell per configuration and dataset, 15 in total; asterisks mark non-overlapping 95% confidence intervals between the two regimes. **c** ConvNeXt-B minus ViT-B/16 AUROC under each regime, six pairs; DINOv2 is excluded because it was not evaluated on ConvNeXt-B. **d** DINOv3 minus ImageNet AUROC under each regime, six pairs. **e** Per-label AUROC change from full fine-tuning to LoRA, one point per label and configuration from 14 labels in ChestX-ray14 and 10 each in CheXpert and MIMIC-CXR, with horizontal bars marking the median per dataset. **f** mAP against AUROC under LoRA, 15 points. AUROC, area under the receiver operating characteristic curve; mAP, mean average precision.

Under full fine-tuning, ConvNeXt-B consistently outperformed ViT-B/16 across adult datasets and resolutions (Table 2), with the gap most apparent at 512 × 512 pixels. For example, with DINOv3 initialization at 512 × 512, ConvNeXt-B reached 82.7 ± 0.2 vs. 82.5 ± 0.1 in MIMIC-CXR, 82.3 ± 0.2 vs. 81.3 ± 0.2 in ChestX-ray14, and 82.5 ± 0.1 vs. 81.9 ± 0.2 in CheXpert. Thus, the main benchmark already indicated a stable backbone advantage under standard end-to-end optimization. Under low-rank adaptation (LoRA)^41^ (Fig. 4a–c and Supplementary Table 4), AUROC fell relative to full fine-tuning in all evaluated dataset-configuration pairs. The magnitude of this loss ranged from 1.3 ± 0.3 to 5.1 ± 0.3 percentage points for ViT-B/16 and from 2.1 ± 0.2 to 4.1 ± 0.3 percentage points for ConvNeXt-B. For DINOv3 specifically, the drop under LoRA was larger for ViT-B/16 than for ConvNeXt-B in all three datasets: 5.1 ± 0.3 vs. 2.8 ± 0.3 in ChestX-ray14, 4.3 ± 0.2 vs. 3.3 ± 0.2 in CheXpert, and 4.1 ± 0.2 vs. 2.1 ± 0.2 in MIMIC-CXR. Thus, reduced adaptation capacity did not preferentially benefit ViT-B/16.

Accordingly, the backbone gap did not narrow under parameter-efficient adaptation. Instead, ConvNeXt-B remained superior to ViT-B/16 in most of the six backbone-initialization pairs, and the gap widened in several cases (Fig. 4c). For DINOv3, the ConvNeXt-B minus ViT-B/16 difference increased from 0.9 to 3.2 percentage points in ChestX-ray14 and from 0.2 to 2.2 percentage points in MIMIC-CXR, while remaining positive in CheXpert (0.6 to 1.6 percentage points). The same qualitative pattern held for ImageNet initialization. These findings indicate that the ConvNeXt advantage cannot be attributed merely to having used full end-to-end fine-tuning in the main benchmark.

The initialization story also remained broadly stable. Under parameter-efficient adaptation, DINOv3 retained an advantage over ImageNet for ConvNeXt-B in all three datasets and for ViT-B/16 in MIMIC-CXR, but this advantage weakened or disappeared for ViT-B/16 in ChestX-ray14 and CheXpert (Fig. 4d). This suggests that ViT-B/16 was more sensitive to the restricted adaptation regime, whereas ConvNeXt-B preserved both its stronger absolute performance and its relative benefit from DINOv3 initialization.

At the label level, parameter-efficient adaptation produced heterogeneous but predominantly negative changes, with median per-label AUROC shifts of about −2.8 percentage points in MIMIC-CXR, −2.8 percentage points in ChestX-ray14, and −2.6 percentage points in CheXpert (Fig. 4e). Importantly, these shifts did not alter the overall ranking of configurations. Class-imbalance-aware evaluation supported the same conclusion: under LoRA, AUROC, and mAP remained strongly aligned across configurations (Spearman *ρ* = 1.00 for MIMIC-CXR, *ρ* = 1.00 for ChestX-ray14, and *ρ* = 0.90 for CheXpert; all *p* ≤ 0.037), indicating that the backbone comparison was not an artifact of AUROC alone (Fig. 4f). The superiority of ConvNeXt-B over ViT-B/16 cannot be explained simply by the use of full fine-tuning. Instead, it persisted under both full and parameter-efficient adaptation, supporting a genuine backbone-level difference in transfer behavior for chest radiograph classification.

### DINOv3’s clean-label advantage diminishes under synthetic label corruption

**Fig. 5:**
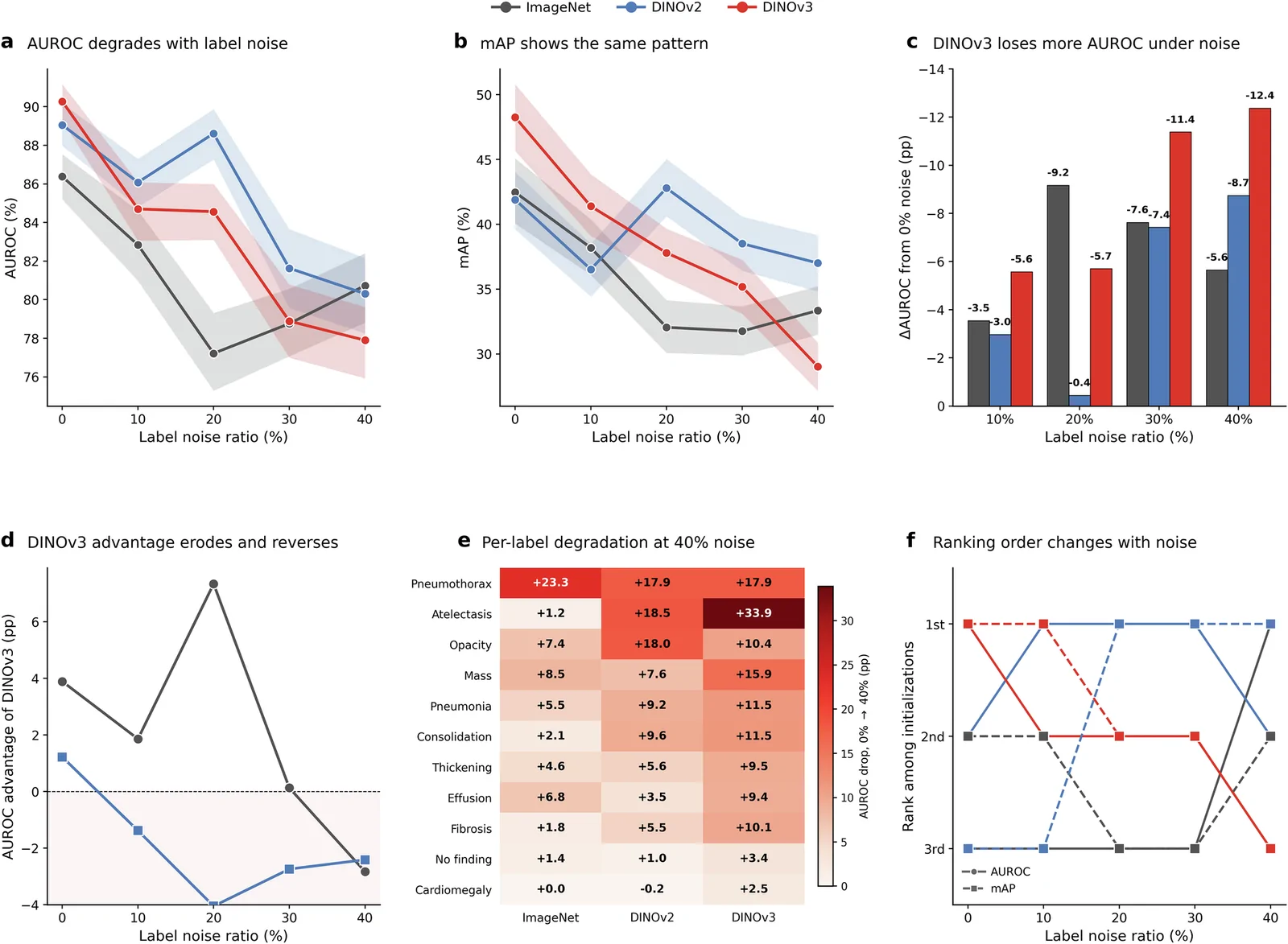
Effect of synthetic label noise on the three initializations. All panels use VinDr-CXR with ViT-B/16 at 512 × 512 pixels, with asymmetric synthetic label noise applied to the training set at five ratios (0, 10, 20, 30, and 40%) and the test set held fixed at *n* = 3000 radiographs. In **a**–**c**, **f** color denotes the initialization, and every value is the mean over 1000 bootstrap resamples of that test set. Macro-averaged AUROC (**a**) and mAP (**b**) against the noise ratio; the shaded bands are the 95% confidence interval, taken as the 2.5th and 97.5th percentiles of the bootstrap distribution. **c** AUROC loss from the clean-label baseline, one bar per initialization at each of the four noise levels. **d** The AUROC difference between DINOv3 and ImageNet (gray) and between DINOv3 and DINOv2 (blue) against the noise ratio, with the region below zero shaded. **e** Per-label AUROC drop from 0 to 40% noise, 33 cells for the 11 VinDr-CXR labels and three initializations, ordered by the mean drop across initializations. **f** Rank of the three initializations at each noise level under AUROC (solid lines) and mAP (dashed lines). **c**, **d**, **f** Carry no error bars. AUROC, area under the receiver operating characteristic curve; mAP, mean average precision.

In the controlled label-noise experiment on VinDr-CXR, DINOv3 gave the strongest performance under clean labels, reaching 90.3 ± 0.5 AUROC, compared with 89.0 ± 0.6 for DINOv2 and 86.4 ± 0.6 for ImageNet (Fig. 5 and Supplementary Table 5). The same ordering was seen for class-imbalance-aware metrics, with DINOv3 also leading in PR-AUC and mAP (47.7 ± 1.4 and 48.3 ± 1.4, respectively).

This advantage did not persist as label corruption increased. DINOv3 degraded more steeply than the other initializations, falling to 84.7 ± 0.8 at 10% noise, 84.6 ± 0.8 at 20%, 78.9 ± 1.0 at 30%, and 77.9 ± 0.9 at 40% noise (Fig. 5a). By comparison, DINOv2 retained higher AUROC at 20, 30, and 40% noise, and ImageNet surpassed DINOv3 at 40% noise (80.7 ± 0.9 vs. 77.9 ± 0.9). The same crossover was visible for mAP, where DINOv3 decreased from 48.3 ± 1.4 under clean labels to 29.0 ± 0.9 at 40% noise, below both DINOv2 (37.0 ± 1.1) and ImageNet (33.4 ± 1.0) (Fig. 5b, f).

Viewed relative to the clean-label baseline, DINOv3 also showed the largest overall loss. Its AUROC dropped by 5.6 points at 10% noise and by 12.4 points at 40% noise, compared with drops of 8.7 points for DINOv2 and 5.6 points for ImageNet at the highest corruption level (Fig. 5c). Retained-performance analysis showed the same pattern: at 40% noise, DINOv3 preserved 86.3 ± 1.0 of its clean-label AUROC, compared with 90.2 ± 1.1 for DINOv2 and 93.5 ± 1.1 for ImageNet (Supplementary Table 5). Thus, although DINOv3 started from the highest clean-label baseline, it was not the most stable initialization under progressive label corruption.

This instability was not uniform across findings. At 40% noise, several labels showed substantially larger degradation for DINOv3 than for the other initializations, most notably atelectasis, where the AUROC drop was 33.9 points for DINOv3 compared with 18.5 for DINOv2 and 1.2 for ImageNet (Fig. 5e). More broadly, DINOv3’s advantage over ImageNet and DINOv2 narrowed with increasing corruption and eventually reversed, so that by 40% noise it ranked last under both AUROC and mAP (Fig. 5d, f). These results establish the label-noise experiment as a boundary-condition analysis and not as a robustness result. DINOv3 was strongest when labels were clean, but its advantage diminished and could reverse under synthetic corruption. Accordingly, the gains observed on weakly labeled adult datasets in the main benchmark should not be interpreted simply as evidence that DINOv3 is intrinsically more robust to noisy supervision.

### DINOv3 generalizes across institutions under external validation

**Fig. 6:**
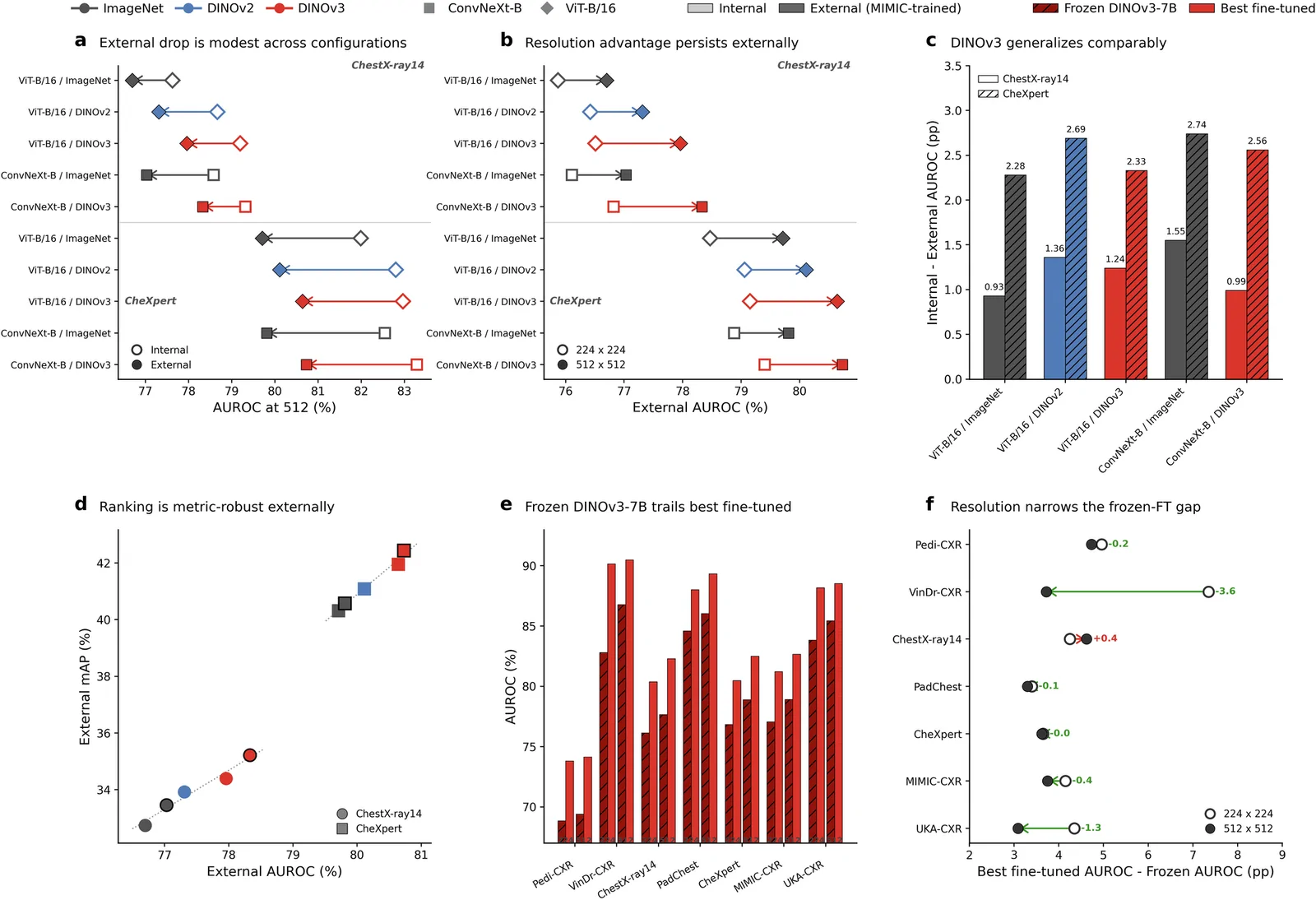
External validation and comparison with frozen DINOv3-7B features. **a**–**d** Cover the five configurations (ViT-B/16 with ImageNet, DINOv2 or DINOv3, and ConvNeXt-B with ImageNet or DINOv3), trained on MIMIC-CXR and tested on ChestX-ray14 and CheXpert, which gives 10 estimates per panel, and there color denotes the initialization; squares denote ConvNeXt-B and diamonds ViT-B/16. **e**, **f** Cover all seven datasets at both resolutions. Every value is the mean over 1000 bootstrap resamples of the relevant test set (*n* = 1397 for Pedi-CXR, 3000 for VinDr-CXR, 25,596 for ChestX-ray14, 22,045 for PadChest, 29,318 for CheXpert, 43,793 for MIMIC-CXR and 40,106 for UKA-CXR), and the figure carries no error bars. **a** Internal and external AUROC at 512 × 512 as dumbbells, open markers for internal and filled markers for external, grouped by target dataset. **b** External AUROC at 224 × 224 (open) and at 512 × 512 (filled). **c** Internal minus external AUROC per configuration, with unhatched bars for ChestX-ray14 and hatched bars for CheXpert. **d** External mAP against external AUROC at 512 × 512, with dotted regression lines and marker shape denoting the target dataset, circles for ChestX-ray14 and squares for CheXpert. **e** AUROC of the frozen DINOv3-7B features with a lightweight classifier (hatched bars) and of the best fine-tuned configuration (solid bars), at 224 × 224 and 512 × 512 for each dataset. **f** Change in the gap between those two from 224 × 224 (open) to 512  × 512 (filled), one dumbbell per dataset, with green arrows marking a narrowing gap and red arrows a widening one. AUROC, area under the receiver operating characteristic curve; mAP, mean average precision.

Under external validation, performance remained close to the corresponding internal test performance in all five evaluated configurations, with AUROC drops of 0.9–2.7 percentage points at 512 × 512 pixels (Fig. 6a–d and Table 4). The smallest external drop was observed on ChestX-ray14, at 0.9 ± 0.3 for ViT-B/16 with ImageNet and 1.0 ± 0.3 for ConvNeXt-B with DINOv3, the latter falling from 79.3 ± 0.2 internally to 78.3 ± 0.2 externally. On CheXpert, the external gaps were somewhat larger but remained modest overall, ranging from 2.3 ± 0.3 to 2.7 ± 0.3.

**Table 4:** External validation across target datasets and resolutions

Resolution	Backbone	Initialization	Internal ChestX-ray14	External ChestX-ray14	Internal- external	Internal CheXpert	External CheXpert	Internal-external
AUROC values								
224 × 224	ViT-B	ImageNet	77.4 ± 0.2	75.9 ± 0.2	1.5 ± 0.4	81.2 ± 0.2	78.5 ± 0.2	2.7 ± 0.3
224 × 224	ViT-B	DINOv2	78.0 ± 0.2	76.4 ± 0.2	1.6 ± 0.3	81.8 ± 0.2	79.1 ± 0.2	2.7 ± 0.3
224 × 224	ViT-B	DINOv3	77.8 ± 0.2	76.5 ± 0.2	1.3 ± 0.3	81.5 ± 0.2	79.2 ± 0.2	2.4 ± 0.3
224 × 224	ConvNeXt-B	ImageNet	77.7 ± 0.2	76.1 ± 0.2	1.6 ± 0.3	81.2 ± 0.2	78.9 ± 0.2	2.3 ± 0.3
224 × 224	ConvNeXt-B	DINOv3	78.0 ± 0.2	76.8 ± 0.2	1.2 ± 0.3	81.9 ± 0.2	79.4 ± 0.2	2.5 ± 0.3
512 × 512	ViT-B	ImageNet	77.6 ± 0.2	76.7 ± 0.2	0.9 ± 0.3	82.0 ± 0.2	79.7 ± 0.2	2.3 ± 0.3
512 × 512	ViT-B	DINOv2	78.7 ± 0.2	77.3 ± 0.2	1.4 ± 0.3	82.8 ± 0.2	80.1 ± 0.2	2.7 ± 0.3
512 × 512	ViT-B	DINOv3	79.2 ± 0.2	78.0 ± 0.2	1.2 ± 0.3	83.0 ± 0.2	80.6 ± 0.2	2.3 ± 0.3
512 × 512	ConvNeXt-B	ImageNet	78.6 ± 0.2	77.0 ± 0.2	1.5 ± 0.3	82.5 ± 0.2	79.8 ± 0.2	2.7 ± 0.3
512 × 512	ConvNeXt-B	DINOv3	79.3 ± 0.2	78.3 ± 0.2	1.0 ± 0.3	83.3 ± 0.2	80.7 ± 0.2	2.6 ± 0.3
PR-AUC values								
224 × 224	ViT-B	ImageNet	33.3 ± 0.3	31.3 ± 0.3	2.0 ± 0.5	42.3 ± 0.3	38.2 ± 0.3	4.1 ± 0.4
224 × 224	ViT-B	DINOv2	34.2 ± 0.3	32.5 ± 0.3	1.7 ± 0.5	43.5 ± 0.3	39.3 ± 0.3	4.2 ± 0.4
224 × 224	ViT-B	DINOv3	34.2 ± 0.3	32.3 ± 0.3	1.9 ± 0.5	42.5 ± 0.3	38.9 ± 0.3	3.6 ± 0.4
224 × 224	ConvNeXt-B	ImageNet	33.6 ± 0.3	31.3 ± 0.3	2.3 ± 0.5	42.0 ± 0.3	38.5 ± 0.3	3.5 ± 0.4
224 × 224	ConvNeXt-B	DINOv3	34.8 ± 0.3	33.0 ± 0.3	1.8 ± 0.5	44.2 ± 0.3	40.0 ± 0.3	4.2 ± 0.5
512 × 512	ViT-B	ImageNet	33.6 ± 0.3	32.7 ± 0.3	1.0 ± 0.5	44.2 ± 0.3	40.3 ± 0.3	4.0 ± 0.5
512 × 512	ViT-B	DINOv2	34.7 ± 0.3	33.9 ± 0.3	0.8 ± 0.5	45.2 ± 0.3	41.0 ± 0.3	4.2 ± 0.5
512 × 512	ViT-B	DINOv3	35.2 ± 0.3	34.3 ± 0.3	0.8 ± 0.5	45.8 ± 0.3	41.9 ± 0.3	3.9 ± 0.5
512 × 512	ConvNeXt-B	ImageNet	34.8 ± 0.3	33.4 ± 0.3	1.4 ± 0.5	44.5 ± 0.3	40.5 ± 0.3	3.9 ± 0.4
512 × 512	ConvNeXt-B	DINOv3	36.1 ± 0.3	35.1 ± 0.3	1.0 ± 0.5	46.9 ± 0.3	42.4 ± 0.3	4.5 ± 0.5
mAP values								
224 × 224	ViT-B	ImageNet	33.4 ± 0.3	31.4 ± 0.3	2.0 ± 0.5	42.4 ± 0.3	38.3 ± 0.3	4.1 ± 0.4
224 × 224	ViT-B	DINOv2	34.3 ± 0.3	32.6 ± 0.3	1.7 ± 0.5	43.5 ± 0.3	39.4 ± 0.3	4.1 ± 0.4
224 × 224	ViT-B	DINOv3	34.2 ± 0.3	32.4 ± 0.3	1.9 ± 0.5	42.6 ± 0.3	39.0 ± 0.3	3.6 ± 0.4
224 × 224	ConvNeXt-B	ImageNet	33.7 ± 0.3	31.4 ± 0.3	2.3 ± 0.5	42.1 ± 0.3	38.5 ± 0.3	3.6 ± 0.4
224 × 224	ConvNeXt-B	DINOv3	34.8 ± 0.3	33.1 ± 0.3	1.8 ± 0.5	44.2 ± 0.3	40.0 ± 0.3	4.2 ± 0.5
512 × 512	ViT-B	ImageNet	33.7 ± 0.3	32.7 ± 0.3	1.0 ± 0.5	44.3 ± 0.3	40.3 ± 0.3	4.0 ± 0.5
512 × 512	ViT-B	DINOv2	34.8 ± 0.3	33.9 ± 0.3	0.8 ± 0.5	45.3 ± 0.3	41.1 ± 0.3	4.2 ± 0.5
512 × 512	ViT-B	DINOv3	35.2 ± 0.3	34.4 ± 0.3	0.8 ± 0.5	45.9 ± 0.3	42.0 ± 0.3	3.9 ± 0.5
512 × 512	ConvNeXt-B	ImageNet	34.8 ± 0.3	33.5 ± 0.3	1.4 ± 0.5	44.5 ± 0.3	40.6 ± 0.3	3.9 ± 0.4
512 × 512	ConvNeXt-B	DINOv3	36.2 ± 0.3	35.2 ± 0.3	1.0 ± 0.5	46.9 ± 0.3	42.4 ± 0.3	4.5 ± 0.5

External evaluation results for models trained on MIMIC-CXR (training *n* = 153,255; validation *n* = 18,139; test *n* = 43,793) and tested on ChestX-ray14 (training *n* = 77,870; validation *n* = 8654; test *n* = 25,596) and CheXpert (training *n* = 115,449; validation *n* = 13,098; test *n* = 29,318) using the overlapping seven-label classification setting comprising cardiomegaly, pleural effusion, pneumonia, atelectasis, no finding, consolidation, and pneumothorax. Results are shown for ViT-B/16 initialized from ImageNet, DINOv2, and DINOv3, and for ConvNeXt-B initialized from ImageNet and DINOv3, each evaluated at 224 × 224 and 512 × 512 resolution. For each configuration, the table reports the internal test AUROC of the model trained on the target dataset itself, together with the external AUROC, precision-recall area under the curve (PR-AUC), and mean average precision (mAP) of the MIMIC-CXR-trained model on ChestX-ray14 and CheXpert.

Importantly, the relative ranking of configurations was largely preserved under domain shift. At 512 × 512, DINOv3 remained the strongest initialization for both backbones on both external target datasets. For ViT-B/16, DINOv3 reached 78.0 ± 0.2 on ChestX-ray14 and 80.6 ± 0.2 on CheXpert, exceeding both ImageNet and DINOv2. The same pattern held for ConvNeXt-B, where DINOv3 achieved the highest external AUROC on ChestX-ray14 (78.3 ± 0.2) and CheXpert (80.7 ± 0.2). Thus, the DINOv3 advantage observed in the internal benchmark was not lost when transferring across institutions.

The resolution effect also persisted externally. Across both target datasets and all configurations, moving from 224 × 224 to 512 × 512 improved external AUROC by 0.8–1.5 percentage points (Fig. 6b and Table 4). For example, ViT-B with DINOv3 improved from 76.5 ± 0.2 to 78.0 ± 0.2 on ChestX-ray14 and from 79.2 ± 0.2 to 80.6 ± 0.2 on CheXpert. ConvNeXt-B with DINOv3 showed a similar pattern, increasing from 76.8 ± 0.2 to 78.3 ± 0.2 on ChestX-ray14 and from 79.4 ± 0.2 to 80.7 ± 0.2 on CheXpert. This indicates that the 512 × 512 advantage was not specific to in-domain evaluation. Class-imbalance-aware metrics supported the same conclusion. External PR-AUC and mAP closely tracked AUROC across configurations, with DINOv3 again ranking highest at 512 × 512 for both backbones and both target datasets. For example, ConvNeXt-B with DINOv3 achieved the best external PR-AUC and mAP on both ChestX-ray14 (35.1 ± 0.3 and 35.2 ± 0.3) and CheXpert (42.4 ± 0.3 and 42.4 ± 0.3), and the external AUROC-mAP relationship remained strongly aligned across institutions (Fig. 6d). These findings show that the main adult benchmark result, namely the advantage of DINOv3 at 512 × 512, remained visible under external validation and was not confined to internal splits.

### Frozen billion-parameter features remain inferior to task-adapted mid-sized models

Despite its much larger scale, the frozen DINOv3-7B encoder consistently underperformed the best task-adapted ViT-B/16 and ConvNeXt-B models across all seven datasets and both universal resolutions (Fig. 6e, f, Supplementary Table 6, and Supplementary Fig. 9). At 224 ×  224, the gap to the best task-adapted model ranged from 3.4 percentage points in PadChest (84.6 ± 0.2 vs. 88.0 ± 0.2) to 7.4 percentage points in VinDr-CXR (82.8 ± 0.8 vs. 90.2 ± 0.6), and reached 5.0 percentage points in Pedi-CXR (68.8 ± 1.3 vs. 73.8 ± 1.1). Similar deficits were seen in adult cohorts, such as ChestX-ray14 (76.1 ± 0.2 vs. 80.4 ± 0.2), CheXpert (76.8 ± 0.2 vs. 80.5 ± 0.2), MIMIC-CXR (77.1 ± 0.2 vs. 81.2 ± 0.1), and UKA-CXR (83.8 ± 0.1 vs. 88.2 ± 0.1).

The same pattern persisted at 512 × 512. Frozen DINOv3-7B reached 69.4 ± 1.3 in Pedi-CXR, 86.8 ± 0.6 in VinDr-CXR, 77.7 ± 0.3 in ChestX-ray14, 78.9 ± 0.2 in CheXpert, 78.9 ± 0.2 in MIMIC-CXR, and 85.4 ± 0.1 in UKA-CXR, whereas the best task-adapted models achieved 74.1 ± 1.2, 90.5 ± 0.5, 82.3 ± 0.2, 82.5 ± 0.1, 82.7 ± 0.2, and 88.5 ± 0.1, respectively. Thus, the frozen-to-fine-tuned gap remained substantial at higher resolution, typically around 3–5 percentage points and reaching 4.6 points in ChestX-ray14 and 3.8 points in MIMIC-CXR.

Increasing resolution from 224 × 224 to 512 × 512 did not close this gap systematically (Fig. 6f). In some datasets the difference narrowed modestly, such as VinDr-CXR and UKA-CXR, whereas in others it remained nearly unchanged or widened slightly, including ChestX-ray14. The overall conclusion therefore did not depend on resolution: in the present setting, larger frozen natural-image representations did not substitute for downstream adaptation. These results show that model scale alone was insufficient to match the performance of task-adapted mid-sized backbones in chest radiograph classification. Even against the 7B-parameter natural-image-pretrained foundation encoder evaluated here, full adaptation of substantially smaller ViT-B/16 and ConvNeXt-B models remained decisively superior, underscoring the importance of domain- and task-specific fine-tuning in medical imaging.

## Discussion

In this large-scale chest radiograph benchmark across *n* = 816,183 images from seven datasets, we found that the benefit of DINOv3 is conditional and not universal. Six observations emerged. First, DINOv3 transferred well to adult chest radiograph classification, but its advantage over DINOv2 was weak at 224 × 224 pixels and became consistent only at 512 × 512 pixels. Second, this pattern did not extend to the pediatric cohort, which showed little sensitivity to either resolution or initialization. Third, scaling further to 1024 × 1024 pixels rarely improved performance and incurred a marked computational penalty. Fourth, ConvNeXt-B remained stronger than ViT-B/16 under both full fine-tuning and parameter-efficient adaptation. Fifth, the 512 × 512 DINOv3 advantage persisted under external validation, but it could not be explained simply by superior robustness to noisy labels. Sixth, frozen 7B features remained clearly inferior to task-adapted mid-sized models. The results show that modern self-supervised pretraining can improve chest radiograph classification, but its value depends on resolution, cohort type, adaptation strategy, and label quality, and not on model scale alone.

Resolution dependence is the central result. At 224 × 224 pixels, DINOv3 generally improved over ImageNet, yet it did not consistently exceed DINOv2. At 512 × 512 pixels, however, DINOv3 became the strongest initialization across most adult cohorts, with the clearest gains seen when paired with ConvNeXt-B. This pattern is consistent with the design of DINOv3, which emphasizes fine-grained feature preservation through Gram-anchored distillation and explicit high-resolution adaptation^22^. It also aligns with the fact that chest radiographs contain clinically relevant structures at much finer spatial scales than are typically preserved in natural-image benchmarks^48^. At the same time, the magnitude of the gain was not uniform across adult datasets, which likely reflects differences in finding composition, label provenance, and cohort characteristics, and not a single dataset-level factor alone. Our cross-dataset finding-group summary supports this interpretation: the largest average gains from 224 × 224 to 512 × 512 were seen for small focal lesions and boundary-dependent findings, whereas large-structure abnormalities, such as cardiomegaly and pleural effusion, changed little. In practical terms, DINOv3 appears to matter most when diagnostic information is spatially subtle.

The pediatric cohort provides an important boundary condition. Pedi-CXR was not merely lower performing overall, but qualitatively different. It showed minimal separation between initializations and almost no consistent improvement from 224 × 224 to 512 × 512 pixels. Several factors may contribute. The cohort is substantially smaller than the adult datasets, contains only three labels, and focuses on a pediatric disease spectrum that differs from the adult multi-label setting^26,37,49^. Moreover, the labels in Pedi-CXR do not emphasize the same small focal and boundary-dependent abnormalities that benefited most from higher resolution in adults. These findings suggest that the adult DINOv3-at-512 pattern should not be generalized automatically to pediatric chest radiography. More broadly, they indicate that transfer behavior in pediatric imaging may be shaped less by high-resolution representation quality alone and more by cohort size, label ontology, and disease phenotype.

Our targeted 1024 × 1024 analysis sharpened this picture by showing that more resolution is not necessarily better. Across the three representative cohorts evaluated at 1024 × 1024, formal cross-resolution testing showed only two significant gains, both in MIMIC-CXR with ConvNeXt-B, whereas the remaining comparisons were neutral or negative. At the same time, the cost increase was substantial: from 512 × 512 to 1024 × 1024, per-epoch training time rose by 6.2-fold overall and by 7.9-fold for ViT-B/16, while total training time to convergence increased by a median of 4.8-fold overall and 8.9-fold for ViT-B/16. In the present setting, 512  × 512 pixels was therefore the best performance-cost operating point. This conclusion should, however, be interpreted within the scope of the efficiency analysis performed here, which was based on measured training time and not on inference latency, GPU memory consumption, or FLOPs. It should also be interpreted within the present architectural setup, as alternative high-resolution designs, such as different patch sizes or adaptive pooling strategies, could potentially shift the resolution-performance trade-off.

The backbone findings were also robust to changes in adaptation regime. Under full fine-tuning, ConvNeXt-B consistently outperformed ViT-B/16, particularly at 512 × 512 pixels. Parameter-efficient adaptation, implemented here with LoRA, did not narrow this gap. Instead, it reduced performance in all evaluated settings and generally affected ViT-B/16 more strongly than ConvNeXt-B, especially for DINOv3 initialization. This suggests that the ConvNeXt advantage cannot be explained simply by the use of full end-to-end fine-tuning. A plausible interpretation is that convolutional inductive biases and hierarchical local feature aggregation remain advantageous in chest radiograph analysis, particularly when diagnostically relevant information is spatially local and when high-resolution inputs emphasize subtle boundaries and fine structures. This interpretation is also consistent with our scaling results, in which ConvNeXt-B incurred a smaller computational penalty than ViT-B/16 at higher resolution and was the only backbone to show significant gains at 1024 × 1024 in MIMIC-CXR. While this does not prove that convolutional backbones are universally superior to transformers in radiology, it does show that, at base scale and under both full and parameter-efficient adaptation, ConvNeXt-B provided the more reliable transfer behavior in our benchmark.

The additional experiments also clarify how DINOv3 should and should not be interpreted in the presence of weak supervision. In the controlled label-noise experiment on VinDr-CXR, DINOv3 performed best under clean labels but degraded more steeply as corruption increased, ultimately falling below both DINOv2 and ImageNet at 40% noise. This is an important cautionary result. The gains we observed on large report-labeled cohorts, such as MIMIC-CXR and CheXpert, should not be attributed simply to superior robustness to noisy labels. Instead, DINOv3 appears to be strongest when the downstream supervision remains reasonably informative, while its advantage can diminish or reverse under sufficiently corrupted targets. In other words, DINOv3 may help on weakly labeled clinical datasets, but that benefit is not equivalent to intrinsic noise immunity.

At the same time, the external-validation experiment shows that the main adult finding is not merely an artifact of within-dataset splits. When models were trained on MIMIC-CXR and evaluated on the fully held-out external datasets ChestX-ray14 and CheXpert using a harmonized seven-label setting, external AUROC drops were modest, and the relative ordering of configurations was largely preserved. DINOv3 remained the strongest initialization at 512 × 512 pixels for both backbones on both external target datasets, and the external advantage of 512 × 512 over 224 × 224 also persisted. Importantly, PR-AUC and mAP tracked AUROC closely in these analyses, arguing against the possibility that the observed ranking depended mainly on the choice of metric. Together, the noise and external-validation results refine the overall interpretation: DINOv3 generalizes across institutions, but its gains are better understood as improved transferable representation quality than as broad robustness to corrupted supervision. At the same time, this experiment used a single training source and a harmonized label subset instead of a full all-to-all cross-dataset design, so broader external generalization should still be tested in future work. The two experiments answer different questions and should be read together. The main benchmark establishes how initialization, backbone, and resolution rank under patient-wise within-dataset generalization, where scanner characteristics, acquisition protocols, demographics, and label-generation procedures are shared between the training and test partitions, and the external experiment shows that this ranking carries across institutions at a cost of 0.9–2.7 AUROC percentage points. Neither result on its own establishes dataset-level generalization to an arbitrary new cohort.

The frozen 7B comparison provides a final practical lesson. Despite its scale, frozen DINOv3-7B with a lightweight classifier was consistently inferior to the best fine-tuned base models across all datasets and both universal resolutions. The gap remained substantial even at 512 × 512 pixels and did not close systematically with higher resolution. This result is not entirely surprising, because the frozen encoder was pretrained on natural images and never adapted to the clinical domain. Still, it is informative: in the present setting, model size alone did not compensate for the absence of task-specific adaptation. For chest radiograph classification, carefully adapted 86–89 million-parameter models delivered more diagnostic value than the much larger frozen vision encoder evaluated here. An important next step will be to compare such frozen natural-image features directly against frozen representations from medical-domain self-supervised models, in order to disentangle the effect of domain mismatch from the broader question of whether frozen representations can approach the performance of task-adapted models in radiology.

From a clinical perspective, the magnitude of the gains in adult cohorts was modest in absolute AUROC terms, often around 0.5–1.0 points, but the distribution of those gains matters. Improvements concentrated in small focal lesions and boundary-dependent findings are more likely to be relevant to emergency, inpatient, and triage settings than improvements on already easy large-structure labels. A system that better resolves subtle pneumothorax, small nodules, or faint focal opacities can be clinically valuable even when the mean AUROC shift seems small. We therefore do not propose a single universal probability threshold from the present benchmark, because the appropriate operating point depends on the intended clinical task, local prevalence, and the relative cost of false-negative and false-positive decisions. In practice, triage-oriented use cases would typically favor higher sensitivity, whereas workflow-prioritization or rule-out settings may require a different trade-off. Our results therefore support a pragmatic recommendation at the model-selection level: when using off-the-shelf self-supervised visual initialization for adult chest radiograph classification, prioritize 512 × 512 inputs and a ConvNeXt-B backbone, and treat further scaling in size or resolution cautiously unless a clear task-specific benefit is shown.

This study has several limitations. First, although we included seven datasets from multiple countries and both public and internal sources, the main benchmark remained limited to AP and PA chest radiographs and to classification tasks; the relevance of these conclusions to lateral views, localization, segmentation, or report generation remains to be established. Indeed, because DINOv3 is designed to preserve dense patch-level structure and our strongest gains were observed for small focal and boundary-dependent findings, extending this analysis to localization and segmentation tasks represents a particularly important future direction. In addition, the relative distribution of AP and PA projections differed across datasets, so projection type remained partially confounded with dataset and may have contributed to apparent cross-dataset differences in performance. Second, the 1024 × 1024 analysis was deliberately targeted instead of exhaustive, and only three cohorts were evaluated at that resolution. This design was chosen because of the substantial computational burden of full high-resolution training and was intended as a focused test of whether scaling beyond 512 × 512 yielded meaningful additional benefit under the present training setup. Third, the backbone comparison under parameter-efficient adaptation was restricted to LoRA and to three adult datasets, so broader conclusions about all parameter-efficient fine-tuning strategies or all model scales would be premature. Fourth, the external-validation analysis used a harmonized seven-label subset and one training source, not a full all-to-all cross-dataset design. Fifth, the frozen-feature comparison was restricted to a natural-image-pretrained DINOv3-7B encoder and did not include frozen medical-domain self-supervised encoders, including emerging DINOv3-based medical adaptations, such as MedDINOv3^25^, so it does not by itself distinguish domain mismatch from a more general limitation of frozen representations. Sixth, the label-noise experiment used synthetic asymmetric corruption in a single dataset, which provides a controlled stress test but cannot capture all properties of real report-derived labeling noise. In addition, class imbalance was handled using inverse-frequency weighting, but we did not systematically compare alternative strategies, such as focal loss, oversampling, or other cost-sensitive objectives, which may be relevant particularly for rare findings. Finally, although class-imbalance-aware metrics supported the main follow-up analyses, AUROC remained the primary metric for the benchmark as a whole, and the study did not include prospective clinical validation or workflow-level evaluation.

In conclusion, DINOv3 improves adult chest radiograph classification most reliably at 512 × 512 pixels, especially when paired with ConvNeXt-B, but this advantage is conditional and not universal. It does not extend clearly to the pediatric cohort; it rarely improves further at 1024 × 1024, it is not explained by superior noise robustness, and it does not remove the need for task-specific fine-tuning. For adult chest radiograph classification, the best trade-off in our benchmark came from fully adapted mid-sized backbones operating at 512 × 512 pixels, whereas larger frozen encoders and higher resolutions offered limited additional value. These findings provide concrete guidance for applying modern self-supervised representations in radiology and clarify where their benefits are most likely to matter in practice.

## Supplementary information

Transparent Peer Review File

Supplementary Information

Description of Additional Supplementary Files

Supplementary Data 1

Supplementary Data 2

Supplementary Data 3

Supplementary Data 4

Supplementary Data 5

Supplementary Data 6

Supplementary Data 7

Supplementary Data 8

Supplementary Data 9

## Data Availability

The datasets used in this study are either publicly accessible, available under controlled access, or internal. The ChestX-ray14 and PadChest datasets are publicly available at https://www.kaggle.com/datasets/nih-chest-xrays/data and https://bimcv.cipf.es/bimcv-projects/padchest/, respectively. The VinDr-CXR and MIMIC-CXR datasets are restricted-access resources hosted on PhysioNet and can be obtained by agreeing to the relevant data protection requirements at https://physionet.org/content/vindr-cxr/1.0.0/ and https://physionet.org/content/mimic-cxr-jpg/2.0.0/. The Pedi-CXR dataset (VinDr-PCXR) is also available through PhysioNet at https://physionet.org/content/vindr-pcxr/1.0.0/. The CheXpert dataset may be requested from Stanford University at https://stanfordmlgroup.github.io/competitions/chexpert/. The UKA-CXR dataset contains patient data from the University Hospital RWTH Aachen, Germany, and is not publicly available due to patient privacy regulations. Access requests can be directed to the corresponding author and are answered within 4 weeks. Access requires a written cooperation agreement that limits use to non-commercial academic research and prohibits redistribution and any attempt at re-identification. A subset of the UKA-CXR dataset is publicly available on Hugging Face via https://huggingface.co/TLAIM. The source data for Figs. 2–6 is available as Supplementary Data 1–9.

## Code availability

All source code, configuration files, and instructions to reproduce the experiments are available at https://github.com/tayebiarasteh/vit-med and archived at^50^. Training and evaluation were performed strictly in full 32-bit floating point (FP32) precision. Experiments were conducted between August 2025 and April 2026.

Implementation details: Python 3.9 with PyTorch 2.8 and torchvision 0.23. Core libraries: NumPy 1.22, SciPy 1.10, scikit-learn 1.2, pandas 1.4, timm 0.6, and OpenCV (cv2) 4.7. Hugging Face tooling: transformers 4.56, huggingface-hub 0.34, datasets 2.19, accelerate 1.10, tokenizers 0.21, and safetensors 0.4.

Pretrained initialization weights were obtained from official public repositories:

 • ImageNet-21K:

    • ViT-B/16 from timm (model identifier: vit_base_patch16_224_in21k), with the corresponding Hugging Face model repository:

      • https://huggingface.co/timm/vit_base_patch16_224.orig_in21k

    • ConvNeXt-B from Hugging Face (loaded using the safetensors format): https://huggingface.co/facebook/convnext-base-224-22k

• DINOv2:

    • ViT-B/16 from Hugging Face, configured with scaled-dot product attention: https://huggingface.co/facebook/dinov2-base

• DINOv3:

    • ViT-B/16 from Hugging Face, configured with scaled-dot product attention: https://huggingface.co/facebook/dinov3-vitb16-pretrain-lvd1689m

    • ViT-7B from Hugging Face, configured with scaled-dot product attention: https://huggingface.co/facebook/dinov3-vit7b16-pretrain-lvd1689m

    • ConvNeXt-B from Hugging Face (loaded using the safetensors format): https://huggingface.co/facebook/dinov3-convnext-base-pretrain-lvd1689m
