# Supplementary material for: Resolution-dependent self-supervised transfer in chest radiograph classification
Source: Commun Med (Lond). 2026 Sep 9;6:479. doi: 10.1038/s43856-026-01897-9 (PMC13558615; doi:10.1038/s43856-026-01897-9)
Supplement: Supplementary file 1 [file 43856_2026_1897_MOESM1_ESM.pdf]

nature portfolio

Peer Review File

Resolution-dependent self-supervised transfer in chest radiograph classification

Corresponding Author: Dr Soroosh Tayebi Arasteh

This file contains all reviewer reports in order by version, followed by all author rebuttals in order by version.

Version 0:

Reviewer comments:

Reviewer #1

(Remarks to the Author)
Areas for Improvement
Major Issues
1. Pediatric Dataset Interpretation
The manuscript states Pedi-CXR showed "no significant improvement in any comparison" but doesn't adequately explore why. Possible explanations warrant discussion:

Is the sample size truly insufficient (n=9,125 isn't trivially small)?
Are pediatric chest radiographs fundamentally different in their feature requirements?
Could the narrow label space (3 labels vs 10-17 in adult datasets) limit the benefit of richer representations?

This finding has important implications for pediatric AI development but feels underexplored.
2. 1024×1024 Analysis Lacks Depth
You tested 1024×1024 on only 3 datasets and report "did not yield systematic gains" but:

No formal statistical tests comparing 512 vs 1024 are presented
The statement "MIMIC-CXR showed a modest rise...while ChestX-ray14 changed little or decreased slightly" needs quantification
Could architectural modifications (different patch sizes, adaptive pooling) unlock benefits at this resolution?

Given the computational cost implications, this deserves more thorough investigation or clearer acknowledgment of the limited scope.
3. Computational Cost Analysis Missing
You mention "substantial computational cost" and "minimal return on cost" but provide no concrete metrics:

What are the actual training times and inference speeds at each resolution?
GPU memory requirements?
FLOPs comparison?

For clinical deployment decisions, efficiency metrics are as critical as AUROC values.
4. Label Noise Inconsistency
You appropriately note that NLP-derived labels in MIMIC-CXR, CheXpert, and ChestX-ray14 "likely attenuate apparent performance gains," but this creates an interpretation challenge. When DINOv3 shows significant improvements on these datasets, is it:

Actually better at the underlying diagnostic task?
Better at learning from noisy labels?
Capturing different features than the NLP system?

This ambiguity deserves explicit discussion.
Minor Issues

5. Writing and Presentation

Line 51-52: "512×512 pixels represent a practical upper limit" contradicts later nuanced discussion about 1024×1024. Consider: "512×512 pixels represent an optimal balance..."
Line 145: "underperform compared with full finetuning" - use "fine-tuning" consistently (you use both forms)
Abstract line 48: "Resolution-related gains were most evident for boundary-dependent and small focal abnormalities" is a key finding but feels buried. Consider elevating this insight.
Figure references: Ensure all supplementary figures cited in text actually exist and are numbered correctly

6. Statistical Reporting

You report "$p \leq 0.0060$" frequently - why this specific threshold? Standard practice would be $p < 0.01$ or $p < 0.001$
Some comparisons show "$p = 0.0060$" exactly across multiple tests, which seems suspiciously uniform. Are these adjusted p-values being rounded?

7. Methods Details

Line 537: "learning rate of 10^-5" - was this optimized, or taken from prior work? A brief justification would strengthen reproducibility
Line 509: "scaled by the maximum" - maximum of what? Maximum pixel value in that image? Across the dataset? Clarify.
Batch sizes: You mention they were "adapted to fit available GPU memory" but don't specify what they were. This affects reproducibility and interpretation.

Technical Questions
8. Class Imbalance Handling
You use inverse-frequency weighting for the loss function. Did you explore other strategies (focal loss, oversampling)? Given the clinical importance of rare findings, this choice impacts interpretation.
9. Architecture-Resolution Interaction
ConvNeXt-B's superiority over ViT-B is interesting, but could this be partially explained by ConvNeXt's better handling of higher resolutions due to its convolutional nature? A brief mechanistic discussion would add insight.
10. External Validation
All seven datasets were used for training and testing (with splits). Did you consider holding out an entire dataset for external validation to test true generalization?
Specific Suggestions
For the Discussion Section

Add a limitations paragraph specifically addressing:

Why 1024×1024 wasn't tested exhaustively
The confounding of projection type (AP vs PA) with dataset
Lack of prospective clinical validation

Expand on clinical implementation: What specific thresholds or operating points would you recommend? The AUROC improvements are modest (0.5-1.0 points) - when does this translate to clinical utility?
Future directions: You mention segmentation and localization briefly (line 400-401). Given that DINOv3 preserves patch-level features, this seems like a natural extension worth emphasizing.

For the Results Section

Table 2: Consider adding a column showing computational cost ratios
Add a figure showing the resolution-AUROC relationship across datasets more directly (currently spread across multiple figures)
Include example images demonstrating the types of findings that benefit most from 512×512 resolution

Minor Corrections

Line 89: "intelligence (AI)" - AI already defined in abstract
Line 293: "dataset-level trends reflect differences in annotation quality more than dataset size" - strong claim that needs either a citation or more supporting evidence
Line 325: formatting issue with citation "straightforwardly extend to medical imaging^25

Reviewer #2

(Remarks to the Author)
This manuscript presents a large-scale benchmark study evaluating DINOv3 for chest radiograph classification across multiple datasets, resolutions, and backbone architectures. The authors systematically compare ImageNet, DINOv2, and DINOv3 initializations on ViT-B/16 and ConvNeXt-B backbones and report that DINOv3, particularly when paired with ConvNeXt-B at 512×512 resolution, achieves the strongest overall performance. The scale of the evaluation and the focus

on resolution effects are valuable contributions. While the experimental effort is substantial and the results are generally well presented, I have several concerns regarding the experimental design and the interpretation of the findings, which should be addressed.

1, All main experiments rely exclusively on full end-to-end fine-tuning of the backbone, which may systematically favor ConvNeXt over ViT architectures. Convolutional models are generally more stable and data-efficient under full fine-tuning, whereas vision transformers often benefit from parameter-efficient fine-tuning (PEFT) strategies such as adapters or LoRA. Consequently, the conclusion that ConvNeXt-B “consistently outperforms” ViT-B may be partly attributable to the chosen optimization regime rather than an inherent architectural advantage. Incorporating PEFT-based baselines, or explicitly acknowledging this limitation, would strengthen the validity of the backbone comparison.

2. As shown in lines 168–182, the reported benefits of DINOv3 initialization and higher input resolution are not uniform across datasets. Although the overall trend holds on average, several adult datasets exhibit weaker or non-significant improvements, and the pediatric cohort shows no measurable benefit. While the authors note the distinct behavior of the pediatric dataset, a more in-depth analysis that explain the observed inconsistencies between adult and pediatric populations, as well as among different adult datasets, can help the readers.

3. The finding that frozen features from DINOv3 underperform fully fine-tuned models is not unexpected, given that DINOv3 is pretrained exclusively on natural images and not adapted to the medical domain. A more informative comparison would be to evaluate frozen features from medical-specific self-supervised models, such as MedDINOv3 [1], to determine whether the observed performance gap reflects a general limitation of frozen representations or is primarily due to domain mismatch.

[1] https://arxiv.org/pdf/2509.02379

Reviewer #3

(Remarks to the Author)
SUMMARY:
In this paper, the authors present an extensive benchmark of DINOv3 for chest x-ray (CXR) classification across seven adult and pediatric datasets. Experiments primarily focus on evaluating DINOv3 compared to its previous iteration, DINOv2, and pre-trained ImageNet model across different input resolutions and vision backbones. Their results indicate the DINOv3 at 512x512 resolution, with diminishing returns after that despite increase in computational cost and that choice of vision backbone is a major factor impacting classifier performance. Additional analysis of label-wise effects (particularly labeling source) demonstrates variability in performance. In conclusion, this work shows the potential for SSL models serving as a strong backbone for CXR classification tasks.

OVERALL IMPRESSION:
My main concern with this work has limited novelty. Prior work (including the authors, as cited) has explored every experiment covered in the paper with much more detail. Extending prior findings to a single task and reporting concordant results is quite incremental, making the paper's conclusions trivial.

SPECIFIC COMMENTS:
- In prior work [1], the authors have explored a very similar experimental setup with DINOv2. Based on a quick skim through it, the only notable additions seem to be DINOv3 and ablations across different input resolutions and vision backbones. Their previous findings and conclusions are near identical, thus making it hard to justify this work's novelty.
- While the authors claim that this paper is the "first systematic evaluation of DINOv3 for chest radiograph classification", other groups have since published DINOv3 validation across numerous medical imaging tasks (including CXR classification) [2].
- Previous work has also extensively evaluated impact of image resolution for CXR classification. Similar to comments raised previously, the findings from this paper are aligned with those reported previously [3]. Beyond the exploration of SOTA SSL technique (vs supervised learning), there are no new conclusions drawn from experiments.
- Expanding upon the prior point, it is quite trivial that DINOv3 would yield superior performance than DINOv2 as higher resolution. The inclusion of a high-resolution post-training phase and passing high-resolution images to the Gram teacher (prior to downsampling) both can be attributed to this improvement and are also extensively discussed in the DINOv3 paper [4].
- Performance improvements in ConvNeXt could be attributed to the data-efficiency and inductive biases of CNNs compared to ViTs. Given the small dataset sizes, it is trivial that ViT would underperform ConvNeXt. For context, the original DINO paper showed superior performance with ViTs [5] and ConvNeXt paper showed comparable performance of Swin Transformers [6]. Without more in-depth analysis, claiming that CNNs are outright better than Transformers for CXR classification may be an overstatement.
- The finding that frozen ImageNet pre-trained DINOv3 with linear probe significantly underperforms fully fine-tuned equivalents is similarly trivial and covered extensively in prior literature [7].
- Could the authors comment on why performance is much lower on pediatric datasets? Is it simply lack of large, high-quality data to yield comparable performance?

REFERENCES:
[1] Tayebi Arasteh, S., Misera, L., Kather, J. N., Truhn, D., & Nebelung, S. (2024). Enhancing diagnostic deep learning via self-supervised pretraining on large-scale, unlabeled non-medical images. European Radiology Experimental, 8(1), 10.

[2] Liu, C., Chen, Y., Shi, H., Lu, J., Jian, B., Pan, J., ... & Arcucci, R. (2025). Does DINOv3 Set a New Medical Vision Standard? Benchmarking 2D and 3D Classification, Segmentation, and Registration. arXiv preprint arXiv:2509.06467.
[3] Sabottke, C. F., & Spieler, B. M. (2020). The effect of image resolution on deep learning in radiography. Radiology: Artificial Intelligence, 2(1), e190015.
[4] Siméoni, O., Vo, H. V., Seitzer, M., Baldassarre, F., Oquab, M., Jose, C., ... & Bojanowski, P. (2025). Dinov3. arXiv preprint arXiv:2508.10104.
[5] Caron, M., Touvron, H., Misra, I., Jégou, H., Mairal, J., Bojanowski, P., & Joulin, A. (2021). Emerging properties in self-supervised vision transformers. In Proceedings of the IEEE/CVF international conference on computer vision (pp. 9650-9660).
[6] Liu, Z., Mao, H., Wu, C. Y., Feichtenhofer, C., Darrell, T., & Xie, S. (2022). A convnet for the 2020s. In Proceedings of the IEEE/CVF conference on computer vision and pattern recognition (pp. 11976-11986).
[7] Davila, A., Colan, J., & Hasegawa, Y. (2024). Comparison of fine-tuning strategies for transfer learning in medical image classification. Image and Vision Computing, 146, 105012.

Reviewer #4

(Remarks to the Author)
Major Comments
1. While the paper evaluates DINOv3 in the context of medical imaging, it does not involve any retraining or adaptation of DINOv3 on medical images. The study simply applies pretrained weights from natural image training to downstream classification tasks, which is essentially a transfer learning evaluation. To strengthen the contribution, a broader and more systematic exploration is warranted.
2. Only one variant of each of the two backbone architectures is evaluated under full finetuning. Given that ViT has six variants and ConvNeXt has four, evaluating only the Base variant is insufficient to support generalizable conclusions about backbone behavior. The same applies to DINOv2. While a frozen ViT-7B is included as an additional comparison, this represents a fundamentally different experimental condition (frozen features with a lightweight head) rather than a systematic scaling analysis, and therefore does not substitute for evaluating multiple finetuned variants across model sizes.
3. It is unclear whether any hyperparameter tuning was performed. In 2D image classification, performance can vary substantially depending on the choice of learning rate, number of training epochs, and other training settings. While batch size may reasonably be constrained by model size, a minimum search over learning rate and epoch count should have been conducted. Given that methodological novelty is limited, comprehensive tuning is necessary for the findings to be considered reliable.
4. Although multiple datasets are used, each dataset appears to be split only into training and test sets without a dedicated validation set. An explicit validation split should be used for hyperparameter selection and model development, separate from the held-out test set. Without this, it is difficult to rule out overfitting to the test set and the evaluation may reflect internal fit rather than true generalization.
5. The primary metric is ROC-AUC; however, for datasets with substantial label imbalance such as ChestX-ray14 and CheXpert, this metric may not be the most appropriate. Metrics that better account for class imbalance, such as mean average precision (mAP) or PR-AUC, should be considered as primary or complementary evaluation criteria.
6. The Methods section contains detailed mathematical descriptions of the DINOv3 pretraining objectives, including Gram anchoring and the associated loss formulations. As these are entirely derived from the original DINOv3 paper and do not constitute a contribution of the present work, we recommend condensing this section considerably. A brief summary with appropriate citation would suffice.

Minor Comment
1. The ViT-B/16 ImageNet-21K weights are referenced via timm, while all other pretrained weights are linked to Hugging Face. For consistency and reproducibility, a corresponding Hugging Face link should also be provided for this model where available, while the timm identifier may be retained as additional reference.

Version 1:

Reviewer comments:

Reviewer #3

(Remarks to the Author)
- The authors have significantly broadened the scope of their work, compared to the previous submission. The inclusion of new analyses, such as: impact of PEFT, pediatric boundary-condition, computational cost trade-off, and synthetic label-noise provide a valuable insight into the scalability of DINOv3 to the chest x-ray domain. I feel that these changes help distinguish the paper from their previous DINOv2-based analysis, justifying sufficient novelty to not be incremental research.

Reviewer #4

(Remarks to the Author)
I appreciate the authors' substantial revision and the addition of external-validation experiments. However, I still have a concern regarding the primary data-splitting strategy. The manuscript describes patient-wise splits within individual datasets and includes a targeted external validation experiment in which models trained on MIMIC-CXR are evaluated on ChestX-

ray14 and CheXpert. While this partly addresses cross-institutional generalization, it does not fully explain why the primary benchmark was designed around within-dataset train/validation/test splits rather than dataset-level holdout splits.

Even when patient-level leakage is avoided, within-dataset splitting can allow scanner characteristics, acquisition protocols, patient demographics, and label-generation procedures to be shared between training and test subsets. Therefore, the primary benchmark should be interpreted mainly as patient-wise within-dataset generalization, complemented by targeted external validation, rather than as a strict dataset-level generalization assessment. I recommend that the authors explicitly justify this design choice and clarify in the Methods or Discussion how it affects the interpretation of the reported transfer-learning performance.

Open Access This Peer Review File is licensed under a Creative Commons Attribution 4.0 International License, which permits use, sharing, adaptation, distribution and reproduction in any medium or format, as long as you give appropriate credit to the original author(s) and the source, provide a link to the Creative Commons license, and indicate if changes were made.
In cases where reviewers are anonymous, credit should be given to 'Anonymous Referee' and the source.
The images or other third party material in this Peer Review File are included in the article’s Creative Commons license, unless indicated otherwise in a credit line to the material. If material is not included in the article’s Creative Commons license and your intended use is not permitted by statutory regulation or exceeds the permitted use, you will need to obtain permission directly from the copyright holder.
To view a copy of this license, visit https://creativecommons.org/licenses/by/4.0/

Reply to Reviewer #1
COMMSMED-25-2461

Title:
Resolution-dependent self-supervised transfer in chest radiograph classification

Comment 1: *"The manuscript states Pedi-CXR showed "no significant improvement in any comparison" but doesn't adequately explore why. Possible explanations warrant discussion:*
Is the sample size truly insufficient (n=9,125 isn't trivially small)?
Are pediatric chest radiographs fundamentally different in their feature requirements? Could the narrow label space (3 labels vs 10-17 in adult datasets) limit the benefit of richer representations?
This finding has important implications for pediatric AI development but feels underexplored."

Authors' Response and Action: We thank the Reviewer for this important comment. We agree that the pediatric finding required substantially deeper analysis and discussion. In the revised manuscript, we therefore elevated Pedi-CXR from a brief exception within the benchmark to a dedicated **Results** subsection and expanded the **Discussion** accordingly. The revised text now makes clear that Pedi-CXR was not simply the lowest-performing cohort, but the only cohort that failed to follow the adult resolution-scaling pattern. We further discuss several plausible contributors, including the smaller cohort size, the much narrower label ontology, and a pediatric disease spectrum that does not emphasize the same small focal and boundary-dependent abnormalities that benefited most from higher resolution in the adult cohorts.

The new **Results** subsection reads as follows:

"The pediatric cohort shows a distinct transfer pattern that does not mirror adult resolution scaling

Pedi-CXR was not simply the weakest-performing dataset, but the only cohort that failed to follow the adult resolution-scaling pattern (**Table 2**). Whereas adult configurations showed a consistent positive lift when moving from 224 × 224 to 512 × 512 pixels, pediatric configurations clustered around zero, with a median gain of only +0.74 percentage points compared with +1.05 percentage points across adult cohorts (**Figure 3a**). This difference was also evident when plotting performance at 224 × 224 vs. 512 × 512 pixels directly: adult configurations lay predominantly above the identity line, whereas the pediatric points remained close to it (**Figure 3b**).
At the dataset level, performance differences between initialization and backbone choices were minimal in Pedi-CXR. At 512 × 512 pixels, all five evaluated configurations fell within a narrow AUROC range of 0.49 percentage points, far smaller than in any adult cohort (**Figure 3c**). Their uncertainty intervals also overlapped broadly

(**Figure 3d**), and none of the pairwise comparisons between DINOv3 and ImageNet or DINOv2 reached significance at either 224 × 224 or 512 × 512 pixels (all $p \geq 0.31$; **Table 2, Supplementary Table 1**). Even the nominally highest pediatric configuration, ViT-B/16 with DINOv2 at 512 × 512, reached only 74.1 ± 1.2, while ConvNeXt-B with DINOv3 reached 73.6 ± 1.2.

This absence of a pediatric resolution effect also held at the label level. None of the three Pedi-CXR labels showed a consistent upward shift from 224 to 512 pixels across configurations (**Figure 3e**). Moreover, when comparing DINOv3 with ImageNet at 512 × 512 using ConvNeXt-B, Pedi-CXR was the only cohort with a negative median label-level effect, whereas all adult datasets showed positive medians (**Figure 3f**). Thus, the adult result that DINOv3 benefits from higher resolution did not transfer to the pediatric setting. These findings indicate that the adult DINOv3-at-512 pattern should not be generalized automatically to pediatric chest radiography. In this cohort, increasing resolution and changing initialization or backbone had little measurable impact, suggesting that the determinants of transfer performance differ materially between pediatric and adult chest X-ray analysis."

Moreover, the **Discussion** now states:

"**Discussion**

[…]

The pediatric cohort provides an important boundary condition. Pedi-CXR was not merely lower performing overall, but qualitatively different. It showed minimal separation between initializations and almost no consistent improvement from 224 × 224 to 512 × 512 pixels. Several factors may contribute. The cohort is substantially smaller than the adult datasets, contains only three labels, and focuses on a pediatric disease spectrum that differs from the adult multi-label setting. Moreover, the labels in Pedi-CXR do not emphasize the same small focal and boundary-dependent abnormalities that benefited most from higher resolution in adults. These findings suggest that the adult DINOv3-at-512 pattern should not be generalized automatically to pediatric chest radiography. More broadly, they indicate that transfer behavior in pediatric imaging may be shaped less by high-resolution representation quality alone and more by cohort size, label ontology, and disease phenotype.

[…]"

Comment 2: *"You tested 1024×1024 on only 3 datasets and report "did not yield systematic gains" but:*

No formal statistical tests comparing 512 vs 1024 are presented

The statement "MIMIC-CXR showed a modest rise...while ChestX-ray14 changed little or decreased slightly" needs quantification

Could architectural modifications (different patch sizes, adaptive pooling) unlock benefits at this resolution?
Given the computational cost implications, this deserves more thorough investigation or clearer acknowledgment of the limited scope.""

Authors': We address **Comments 2** and **3** together in one response

Comment 3: *"You mention "substantial computational cost" and "minimal return on cost" but provide no concrete metrics:*
What are the actual training times and inference speeds at each resolution?
GPU memory requirements?
FLOPs comparison?
For clinical deployment decisions, efficiency metrics are as critical as AUROC values."

Authors' Response and Action: We address Comments 2 and 3 together, as both concern the depth of the high-resolution analysis and the lack of explicit efficiency metrics in the previous version. We agree that the original manuscript did not analyze the 1024 × 1024 experiments with sufficient depth. In the revised manuscript, we therefore expanded this part substantially in both the **Results** and **Discussion.** Specifically, we now provide formal 512 × 512 vs. 1024 × 1024 cross-resolution comparisons for all evaluated configurations, quantify the magnitude and direction of the observed changes, and add a dedicated computational efficiency analysis based on measured per-epoch training time and total wall-clock time to convergence on the same hardware platform.

These new analyses show that only 2 of the 15 evaluated dataset-configuration pairs yielded significant gains, both in MIMIC-CXR with ConvNeXt-B, whereas the remaining comparisons were neutral or negative. At the same time, increasing resolution from 512 × 512 to 1024 × 1024 increased per-epoch training time by 6.2 ± 4.4-fold overall and total wall-clock time to convergence by a median of 4.8-fold overall, with a substantially larger penalty for ViT-B/16 than for ConvNeXt-B. We also now state explicitly in the **Discussion** that the 1024 × 1024 analysis was targeted rather than exhaustive and should be interpreted within the present training setup.

Regarding the efficiency request in **Comment 3**, the revised manuscript now reports concrete training-time metrics, both as configuration-specific ratios and as summarized scaling behavior across experiments. We agree that inference speed, GPU memory requirements, and FLOPs would also be valuable for deployment-oriented analysis. However, these quantities were not collected systematically in the present study and therefore are not added to the revised manuscript. We would prefer not to overstate what was measured. Instead, we now frame the efficiency analysis explicitly around the training-time metrics that were actually recorded. Finally, we agree that alternative architectural modifications, such as different patch sizes or adaptive pooling, could potentially alter the behavior at 1024 × 1024 resolution, but these were beyond the

scope of the present study and are now acknowledged as an important direction for future work.

In the **Results** section, we now have the following subsection in this regard:
"Scaling beyond 512 × 512 yields limited benefit despite sharply increased computational cost
We next examined whether the adult 512 × 512 advantage extended to 1024 × 1024 training in the three cohorts evaluated at this resolution, namely Pedi-CXR, ChestX-ray14, and MIMIC-CXR (**Figure 2g-i, Table 3, Supplementary Table 3**). Formal 512 vs. 1024 comparison showed that additional scaling rarely improved performance. Across the 15 evaluated dataset-configuration pairs, only two yielded significant gains, both in MIMIC-CXR with ConvNeXt-B: ImageNet increased from 82.2 ± 0.1 to 83.2 ± 0.1 (ΔAUROC = +1.0 ± 0.2) and DINOv3 from 82.7 ± 0.2 to 83.3 ± 0.1 (ΔAUROC = +0.7 ± 0.2). All remaining comparisons were neutral or negative, including clear degradations for several ViT-B/16 settings, such as Pedi-CXR with ImageNet (73.7 ± 1.2 to 65.8 ± 1.4; ΔAUROC = -7.9 ± 1.8) and ChestX-ray14 with DINOv2 (80.0 ± 0.2 to 73.4 ± 0.2; ΔAUROC = -6.6 ± 0.3).
The overall pattern was therefore not one of continued monotonic improvement with increasing resolution. In Pedi-CXR, 1024 × 1024 provided no measurable benefit for any configuration. In ChestX-ray14, performance changes were small for ConvNeXt-B and negative for all ViT-B/16 initializations. Only MIMIC-CXR showed a modest positive shift, and even there the gains were confined to ConvNeXt-B and remained well below one AUROC point for DINOv3 (**Figure 2g, Table 3**). Thus, while DINOv3 retained its strong relative standing at 1024 × 1024 wherever tested against alternative initializations at the same resolution, scaling beyond 512 × 512 did not produce a consistent cross-dataset accuracy benefit.
These limited returns contrasted sharply with the computational cost of 1024-pixel training. Across all full fine-tuning experiments, moving from 512 × 512 to 1024 × 1024 increased per-epoch training time by 6.2 ± 4.4-fold overall, with a larger penalty for ViT-B/16 than for ConvNeXt-B (7.9 ± 5.1-fold vs. 3.6 ± 0.9-fold; **Supplementary Table 3**). The effect on end-to-end training time was even more pronounced: the median total wall-clock ratio for 1024 × 1024 vs. 512 × 512 was 4.8 overall, but 8.9 for ViT-B/16 and 2.9 for ConvNeXt-B. In the targeted 1024 × 1024 experiments, this translated to mean total training-time ratios of 11.1 ± 5.5 for ViT-B/ImageNet, 13.9 ± 7.9 for ViT-B/DINOv2, 6.7 ± 1.9 for ViT-B/DINOv3, 3.1 ± 1.1 for ConvNeXt-B/ImageNet, and 3.5 ± 1.2 for ConvNeXt-B/DINOv3 (**Table 3, Figure 2h,i**). Under the present training setup, 1024 × 1024 training rarely improves classification performance enough to justify its substantially higher cost. The practical operating point of the benchmark therefore remained at 512 × 512 pixels, where DINOv3 already achieved its most reproducible gains across adult cohorts without incurring the marked efficiency penalty of further resolution scaling."

Relevant paragraph in **Discussion**:

"Our targeted 1024 × 1024 analysis sharpened this picture by showing that more resolution is not necessarily better. Across the three representative cohorts evaluated at 1024 × 1024, formal cross-resolution testing showed only two significant gains, both in MIMIC-CXR with ConvNeXt-B, whereas the remaining comparisons were neutral or negative. At the same time, the cost increase was substantial: from 512 × 512 to 1024 × 1024, per-epoch training time rose by 6.2-fold overall and by 7.9-fold for ViT-B/16, while total training time to convergence increased by a median of 4.8-fold overall and 8.9-fold for ViT-B/16. In the present setting, 512 × 512 pixels was therefore the best performance-cost operating point. This conclusion should, however, be interpreted within the scope of the efficiency analysis performed here, which was based on measured training time rather than inference latency, GPU memory consumption, or FLOPs. It should also be interpreted within the present architectural setup, as alternative high-resolution designs, such as different patch sizes or adaptive pooling strategies, could potentially shift the resolution-performance trade-off."

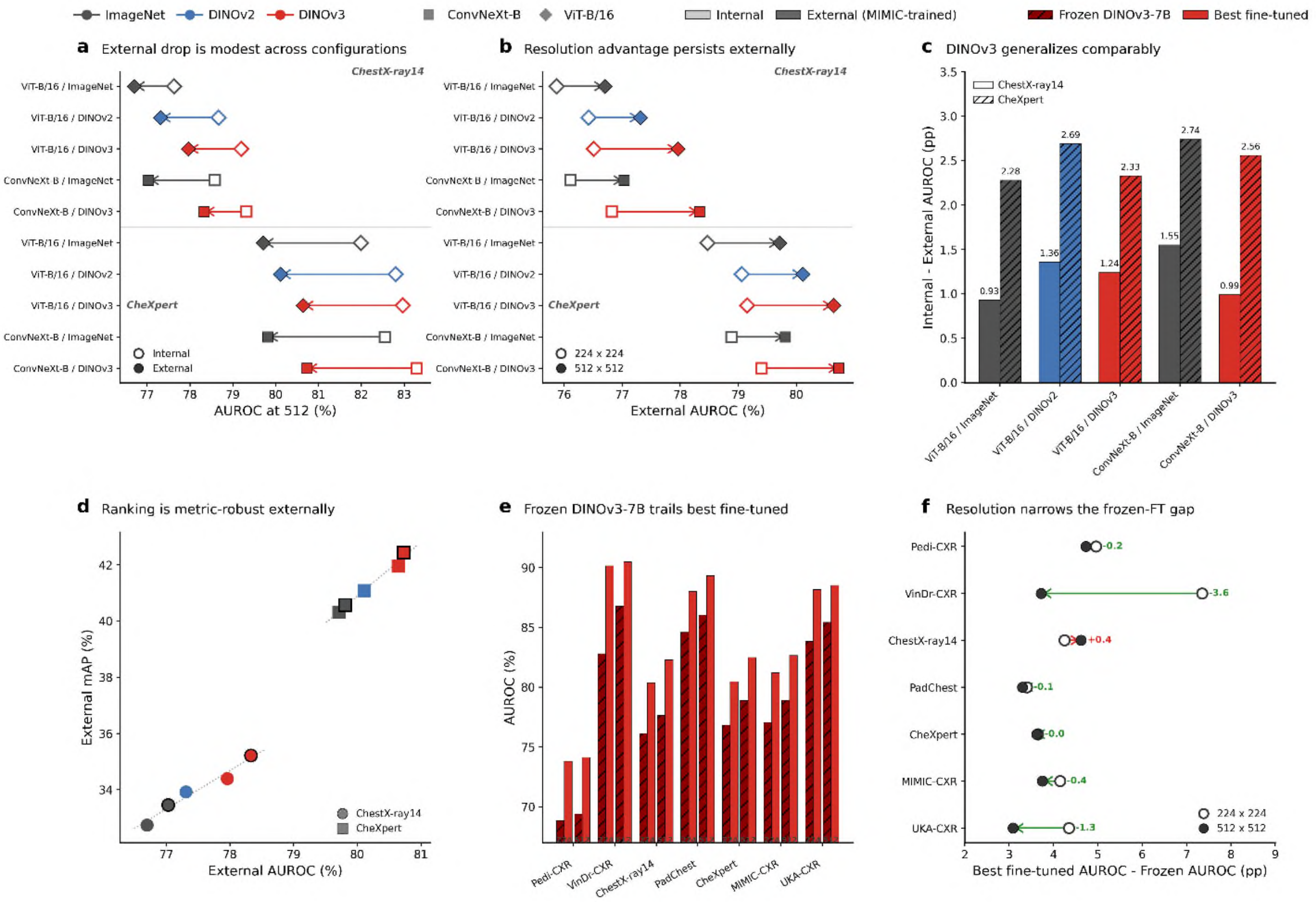

Figure 6: External validation and frozen feature comparison. **a** Internal vs. external AUROC at 512×512 for five configurations tested on ChestX-ray14 (test n=25,596), and CheXpert (test n=29,318) after training on MIMIC-CXR (training n=153,255; validation n=18,139; test n=43,793). Dumbbells show internal performance (open circles) and external performance (filled circles) connected by arrows indicating the magnitude of domain shift. External drops range from 0.9 to 2.7 percentage points. ConvNeXt-B/DINOv3 exhibits the smallest external drop on ChestX-ray14 (0.99 pp). **b** Resolution advantage from 224×224 (open) to 512×512 (filled) persists on external test sets, with lifts ranging

from +0.83 to +1.51 pp across all configurations and both target datasets. **c** Internal-external AUROC gap per configuration, grouped by target dataset. Hatching distinguishes CheXpert from ChestX-ray14. DINOv3 configurations generalize comparably to or better than ImageNet baselines. **d** External mAP vs. AUROC at 512×512 for all configuration-dataset combinations. Dotted regression lines show strong ranking correspondence. Dataset shapes (circles for ChestX-ray14, squares for CheXpert) confirm metric robustness holds across institutions, addressing concerns about AUROC reliability under class imbalance. **e** Frozen DINOv3-7B linear probe performance (hatched bars) vs. best fine-tuned configuration (solid bars) across seven datasets at 224×224 and 512×512. Frozen features consistently lag fine-tuned mid-sized models by 3 to 7 percentage points at both resolutions. Dataset order: Pedi-CXR, VinDr-CXR, ChestX-ray14, PadChest, CheXpert, MIMIC-CXR, UKA-CXR. **f** Change in frozen-to-fine-tuned gap from 224×224 (open) to 512×512 (filled) per dataset. Green arrows indicate gap narrowing; red indicates widening.

Supplementary Table 3: Relative computational scaling across resolution transitions, backbones, and initialization strategies. Summary of computational scaling for all full fine-tuning experiments in this study, expressed as resolution-transition ratios. For each transition, metric, backbone group, and initialization group, the table reports the mean ratio ± standard deviation (SD), median ratio, minimum ratio, maximum ratio, and the number of evaluated configurations (n). Ratios are shown for both per-epoch training time and total wall-clock training time to convergence. DINOv2 ratios are reported only for ViT-B/16, as DINOv2 was not evaluated with ConvNeXt-B. Results are shown for all seven datasets: Pedi-CXR (training n=7,728; test n=1,397), VinDr-CXR (training n=15,000; test n=3,000), ChestX-ray14 (training n=77,870; validation n=8,654; test n=25,596), PadChest (training n=79,697; validation n=8,783; test n=22,045), CheXpert (training n=115,449; validation n=13,098; test n=29,318), MIMIC-CXR (training n=153,255; validation n=18,139; test n=43,793), and UKA-CXR (training n=137,902; validation n=15,353; test n=40,106).

Transition	Metric	Backbone	Initialization	Ratio (mean ± SD)	Ratio (Median	Ratio (Minimum)	Ratio (Maximum)	n
224 → 512	Epoch time	All	All	3.3 ± 1.3	2.8	1.6	7.0	35
224 → 512	Epoch time	ViT-B/16	All	3.5 ± 1.4	3.0	1.6	7.0	21
224 → 512	Epoch time	ConvNeXt-B	All	3.0 ± 1.0	2.5	2.1	4.8	14
224 → 512	Epoch time	ViT-B/16	ImageNet	3.1 ± 1.2	3.0	1.6	5.2	7
224 → 512	Epoch time	ViT-B/16	DINOv2	3.7 ± 1.8	2.8	2.4	7.0	7
224 → 512	Epoch time	ViT-B/16	DINOv3	3.7 ± 1.3	3.1	2.7	6.2	7
224 → 512	Epoch time	ConvNeXt-B	ImageNet	2.9 ± 0.9	2.5	2.2	4.8	7
224 → 512	Epoch time	ConvNeXt-B	DINOv3	3.0 ± 1.1	2.6	2.1	4.8	7
224 → 512	Total time to converge	All	All	3.6 ± 1.5	3.5	0.6	7.9	35
224 → 512	Total time to converge	ViT-B/16	All	4.0 ± 1.6	4.1	0.6	7.9	21
224 → 512	Total time to converge	ConvNeXt-B	All	3.0 ± 1.3	2.8	1.4	4.8	14
224 → 512	Total time to converge	ViT-B/16	ImageNet	4.4 ± 1.4	4.9	1.8	5.7	7
224 → 512	Total time to converge	ViT-B/16	DINOv2	4.1 ± 2.3	4.1	0.6	7.9	7
224 → 512	Total time to converge	ViT-B/16	DINOv3	3.6 ± 0.7	3.5	2.5	4.5	7
224 → 512	Total time to converge	ConvNeXt-B	ImageNet	2.7 ± 1.2	2.7	1.5	4.8	7
224 → 512	Total time to converge	ConvNeXt-B	DINOv3	3.4 ± 1.4	4.0	1.4	4.8	7
512 → 1024	Epoch time	All	All	6.2 ± 4.4	5.1	2.4	18.8	15
512 → 1024	Epoch time	ViT-B/16	All	7.9 ± 5.1	6.0	3.2	18.8	9
512 → 1024	Epoch time	ConvNeXt-B	All	3.6 ± 0.9	3.6	2.4	5.1	6
512 → 1024	Epoch time	ViT-B/16	ImageNet	9.3 ± 8.3	6.0	3.2	18.8	3
512 → 1024	Epoch time	ViT-B/16	DINOv2	6.2 ± 2.4	6.5	3.7	8.3	3
512 → 1024	Epoch time	ViT-B/16	DINOv3	8.1 ± 4.5	5.7	5.4	13.3	3
512 → 1024	Epoch time	ConvNeXt-B	ImageNet	4.2 ± 0.8	3.7	3.7	5.1	3
512 → 1024	Epoch time	ConvNeXt-B	DINOv3	3.1 ± 0.6	3.3	2.4	3.5	3
512 → 1024	Total time to converge	All	All	7.7 ± 5.8	4.8	2.2	18.9	15
512 → 1024	Total time to converge	ViT-B/16	All	10.6 ± 5.8	8.9	4.7	18.9	9
512 → 1024	Total time to converge	ConvNeXt-B	All	3.3 ± 1.1	2.9	2.2	4.8	6
512 → 1024	Total time to converge	ViT-B/16	ImageNet	11.1 ± 5.5	14.0	4.8	14.6	3
512 → 1024	Total time to converge	ViT-B/16	DINOv2	13.9 ± 7.9	18.0	4.7	18.9	3
512 → 1024	Total time to converge	ViT-B/16	DINOv3	6.7 ± 1.9	5.7	5.4	8.9	3
512 → 1024	Total time to converge	ConvNeXt-B	ImageNet	3.1 ± 1.1	2.8	2.2	4.4	3
512 → 1024	Total time to converge	ConvNeXt-B	DINOv3	3.5 ± 1.2	3.0	2.5	4.8	3
224 → 1024	Epoch time	All	All	17.4 ± 10.3	16.4	7.2	40.4	15
224 → 1024	Epoch time	ViT-B/16	All	22.4 ± 10.6	18.2	9.6	40.4	9
224 → 1024	Epoch time	ConvNeXt-B	All	10.0 ± 2.0	10.1	7.2	12.1	6
224 → 1024	Epoch time	ViT-B/16	ImageNet	22.1 ± 16.2	16.4	9.6	40.4	3
224 → 1024	Epoch time	ViT-B/16	DINOv2	19.7 ± 1.5	20.1	18	20.8	3
224 → 1024	Epoch time	ViT-B/16	DINOv3	25.3 ± 12.7	18.2	17.8	40.0	3
224 → 1024	Epoch time	ConvNeXt-B	ImageNet	11.2 ± 1.6	12.0	9.4	12.1	3
224 → 1024	Epoch time	ConvNeXt-B	DINOv3	8.8 ± 1.9	8.3	7.2	10.9	3
224 → 1024	Total time to converge	All	All	29.7 ± 26.3	20.9	4.6	94.0	15
224 → 1024	Total time to converge	ViT-B/16	All	42.9 ± 26.4	37.5	17.8	94.0	9
224 → 1024	Total time to converge	ConvNeXt-B	All	9.9 ± 6.2	7.3	4.6	20.9	6
224 → 1024	Total time to converge	ViT-B/16	ImageNet	40.2 ± 26.4	25.2	24.6	70.7	3
224 → 1024	Total time to converge	ViT-B/16	DINOv2	63.3 ± 28.6	58.5	37.5	94.0	3
224 → 1024	Total time to converge	ViT-B/16	DINOv3	25.3 ± 12.7	18.2	17.8	40.0	3
224 → 1024	Total time to converge	ConvNeXt-B	ImageNet	6.9 ± 1.0	6.5	6.1	8.0	3
224 → 1024	Total time to converge	ConvNeXt-B	DINOv3	13.0 ± 8.2	13.6	4.6	20.9	3

Comment 4: *"You appropriately note that NLP-derived labels in MIMIC-CXR, CheXpert, and ChestX-ray14 "likely attenuate apparent performance gains," but this creates an interpretation challenge. When DINOv3 shows significant improvements on these datasets, is it:*
Actually better at the underlying diagnostic task?
Better at learning from noisy labels?
Capturing different features than the NLP system?
This ambiguity deserves explicit discussion."

Authors' Response and Action: We thank the Reviewer for highlighting this important interpretive issue. We agree that, in the previous version, the improvements observed on report-labeled datasets such as MIMIC-CXR, CheXpert, and ChestX-ray14 could be interpreted in multiple ways and required more explicit discussion. This concern was one of the main motivations for adding the controlled synthetic label-noise experiment in the revised manuscript. In the current version, we now address this directly in both the **Results** and the **Discussion**.
Specifically, the new VinDr-CXR label-noise analysis shows that DINOv3 performs best under clean labels but degrades more steeply as synthetic corruption increases, ultimately falling below both DINOv2 and ImageNet at high noise levels. We therefore do not interpret the gains observed on weakly labeled adult datasets as evidence that DINOv3 is intrinsically more robust to noisy supervision. Instead, the revised manuscript now adopts a more cautious interpretation: DINOv3 appears to improve transferable representation quality when downstream supervision remains reasonably informative, but this is not equivalent to broad robustness against corrupted labels. In other words, the current data support the conclusion that DINOv3 is better at the downstream task under the label conditions present in these datasets, but they do not support the stronger claim that its advantage arises from superior noise tolerance per se.

In the **Results** section, we now have the following subsection in this regard:
"DINOv3's clean-label advantage diminishes under synthetic label corruption
To test whether DINOv3's gains on adult datasets could be explained by superior robustness to weak supervision, we performed a controlled label-noise experiment on VinDr-CXR using ViT-B/16 at 512 × 512 pixels (**Figure 5, Supplementary Table 5**). Under clean labels, DINOv3 gave the strongest performance, reaching 90.3 ± 0.5 AUROC, compared with 89.0 ± 0.6 for DINOv2 and 86.4 ± 0.6 for ImageNet. The same ordering was seen for class-imbalance-aware metrics, with DINOv3 also leading in PR-AUC and mAP (47.7 ± 1.4 and 48.3 ± 1.4, respectively).
This advantage did not persist as label corruption increased. DINOv3 degraded more steeply than the other initializations, falling to 84.7 ± 0.8 at 10% noise, 84.6 ± 0.8 at 20%, 78.9 ± 1.0 at 30%, and 77.9 ± 0.9 at 40% noise (**Figure 5a**). By comparison, DINOv2 retained higher AUROC at 20%, 30%, and 40% noise, and ImageNet surpassed DINOv3 at 40% noise (80.7 ± 0.9 vs. 77.9 ± 0.9). The same crossover was

visible for mAP, where DINOv3 decreased from 48.3 ± 1.4 under clean labels to 29.0 ± 0.9 at 40% noise, below both DINOv2 (37.0 ± 1.1) and ImageNet (33.4 ± 1.0) (**Figure 5b,f**).

Viewed relative to the clean-label baseline, DINOv3 also showed the largest overall loss. Its AUROC dropped by 5.6 points at 10% noise and by 12.4 points at 40% noise, compared with drops of 8.7 points for DINOv2 and 5.6 points for ImageNet at the highest corruption level (**Figure 5c**). Retained-performance analysis showed the same pattern: at 40% noise, DINOv3 preserved 86.3 ± 1.0 of its clean-label AUROC, compared with 90.2 ± 1.1 for DINOv2 and 93.5 ± 1.1 for ImageNet (**Supplementary Table 5**). Thus, although DINOv3 started from the highest clean-label baseline, it was not the most stable initialization under progressive label corruption.

This instability was not uniform across findings. At 40% noise, several labels showed substantially larger degradation for DINOv3 than for the other initializations, most notably atelectasis, where the AUROC drop was 33.9 points for DINOv3 compared with 18.5 for DINOv2 and 1.2 for ImageNet (**Figure 5e**). More broadly, DINOv3's advantage over ImageNet and DINOv2 narrowed with increasing corruption and eventually reversed, so that by 40% noise it ranked last under both AUROC and mAP (**Figure 5d,f**). These results establish the label-noise experiment as a boundary-condition analysis rather than a robustness result. DINOv3 was strongest when labels were clean, but its advantage diminished and could reverse under synthetic corruption. Accordingly, the gains observed on weakly labeled adult datasets in the main benchmark should not be interpreted simply as evidence that DINOv3 is intrinsically more robust to noisy supervision."

The relevant paragraph in **Discussion**:

"The additional experiments also clarify how DINOv3 should and should not be interpreted in the presence of weak supervision. In the controlled label-noise experiment on VinDr-CXR, DINOv3 performed best under clean labels but degraded more steeply as corruption increased, ultimately falling below both DINOv2 and ImageNet at 40% noise. This is an important cautionary result. The gains we observed on large report-labeled cohorts such as MIMIC-CXR and CheXpert should not be attributed simply to superior robustness to noisy labels. Instead, DINOv3 appears to be strongest when the downstream supervision remains reasonably informative, while its advantage can diminish or reverse under sufficiently corrupted targets. In other words, DINOv3 may help on weakly labeled clinical datasets, but that benefit is not equivalent to intrinsic noise immunity."

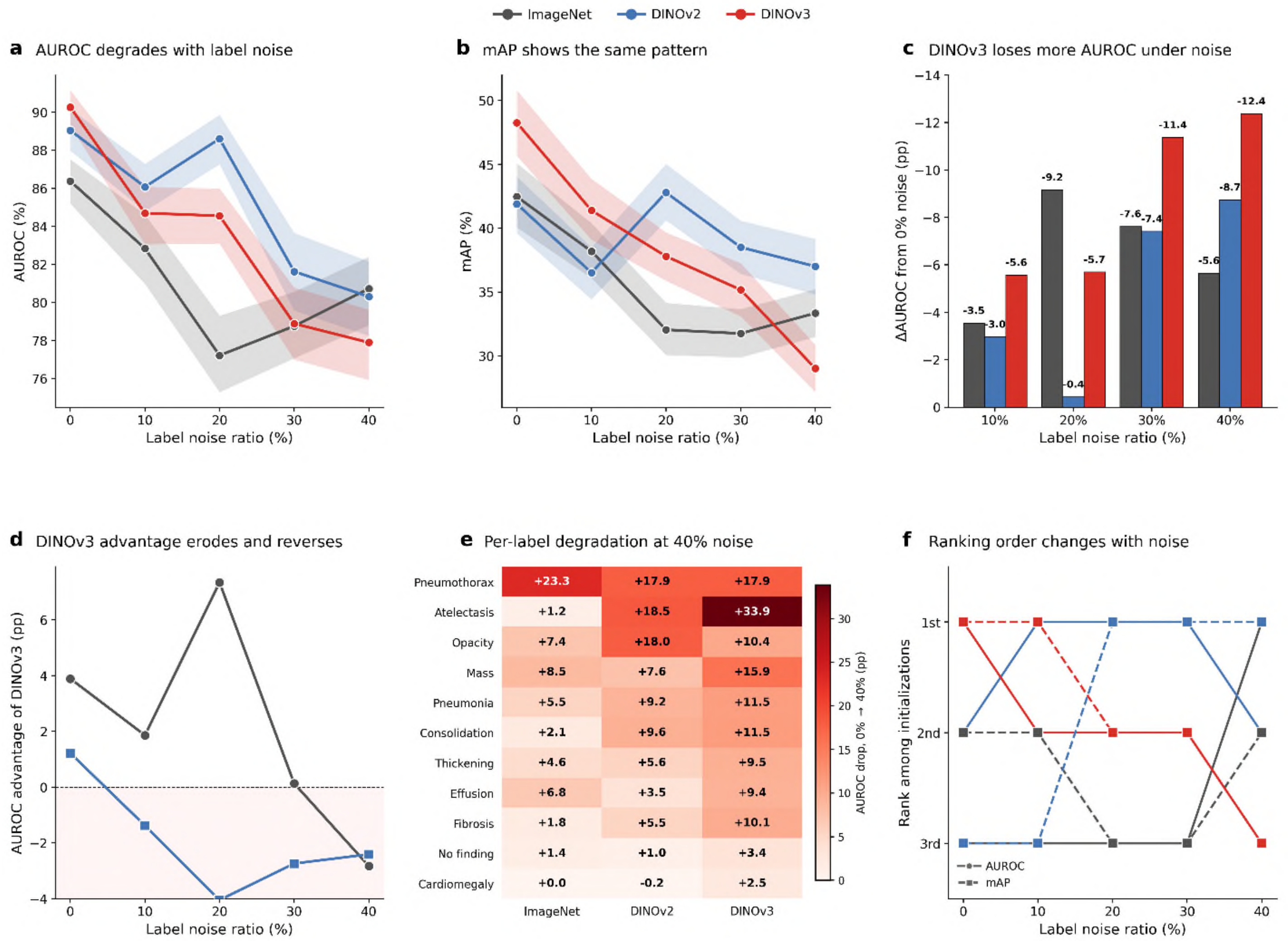

Figure 5: DINOv3's advantage over earlier initializations diminishes and reverses under label noise. All panels use VinDr-CXR (full training set contains n=15,000; test set is fixed at n=3,000 for all experiments) (ViT-B/16, 512 × 512) with synthetic symmetric label noise introduced on the training set at five ratios (0, 10, 20, 30, 40%). **a** Macro-averaged AUROC as a function of label noise ratio for each initialization, with 95% bootstrap confidence intervals shown as shaded bands. DINOv3 achieves the highest clean-label AUROC (90.27%) but degrades most steeply, falling below ImageNet at 40% noise. **b** Macro-averaged mAP as a function of noise ratio. The same crossover pattern holds under a class-imbalance-aware metric. **c** AUROC loss from the clean-label baseline at each noise level, per initialization. DINOv3 incurs the largest losses at three of the four noise levels and the largest loss overall (−12.4 pp at 40% noise). **d** DINOv3's AUROC advantage over ImageNet and over DINOv2 as a function of noise. The advantage over ImageNet starts at +3.9 pp, peaks at +7.3 pp at 20% noise, and reverses to −2.8 pp by 40%. The red-shaded region marks the range where DINOv3 has lost its advantage. **e** Per-label AUROC drop from 0% to 40% label noise, ordered by mean drop across initializations. Atelectasis shows an especially asymmetric pattern: ImageNet loses only 1.2 pp while DINOv3 loses 33.9 pp. **f** Configuration ranking at each noise level under AUROC (solid lines) and mAP (dashed lines). DINOv3 is the best-ranked initialization on clean labels under both metrics but drops to third at 40% noise under AUROC and to third under mAP.

Comment 5: *“Line 51-52: "512×512 pixels represent a practical upper limit" contradicts later nuanced discussion about 1024×1024. Consider: "512×512 pixels represent an optimal balance..."”*

> **Authors’ Response and Action**: We agree with this wording suggestion. The revised manuscript no longer frames 512 × 512 as a hard upper limit. Instead, it now describes 512 × 512 as the best performance-cost operating point or the best trade-off under the

present training setup. This wording is used consistently in the revised **Results**, **Discussion**, and abstract-level summary statements.

Comment 6: *“Line 145: "underperform compared with full finetuning" - use "fine-tuning" consistently (you use both forms)”*

Authors’ Response and Action: We agree. This terminology has been standardized in the revised manuscript, and “fine-tuning” is now used consistently in the main text.

Comment 7: *“Abstract line 48: "Resolution-related gains were most evident for boundary-dependent and small focal abnormalities" is a key finding but feels buried. Consider elevating this insight.”*

Authors’ Response and Action: We agree that this is an important biological and clinical interpretation of the benchmark and that it should appear more prominently in the Abstract. In the revised version, we have elevated this point within the Results portion of the **Abstract** rather than leaving it implicit or secondary. The current Results of **Abstract** now states:

“In adult cohorts, DINOv3 did not consistently outperform DINOv2 at 224 × 224 pixels, but became the strongest initialization at 512 × 512 pixels, especially with ConvNeXt-B. Gains were greatest for small focal and boundary-dependent abnormalities, whereas large-structure findings changed little. The pediatric cohort showed no significant benefit from DINOv3, higher resolution, or backbone choice. Scaling to 1024 × 1024 rarely improved performance and markedly increased computational cost. ConvNeXt-B remained superior to ViT-B/16 under both full and parameter-efficient adaptation. External validation preserved the 512 × 512 DINOv3 advantage, whereas synthetic label corruption showed that this benefit should not be interpreted simply as superior noise robustness. Frozen DINOv3-7B features underperformed relative to fully adapted 86 to 89M-parameter backbones.”

Comment 8: *“Figure references: Ensure all supplementary figures cited in text actually exist and are numbered correctly”*

Authors’ Response and Action: We have carefully checked all figure and supplementary figure citations throughout the revised manuscript and corrected the

cross-references where needed to ensure that all cited supplementary figures exist and are numbered consistently with the final submission.

Comment 9: *“You report "p ≤ 0.0060" frequently - why this specific threshold? Standard practice would be p < 0.01 or p < 0.001*
Some comparisons show "p = 0.0060" exactly across multiple tests, which seems suspiciously uniform. Are these adjusted p-values being rounded?”

Authors’ Response and Action: We thank the Reviewer for raising this point. The repeated value 0.006 is not being used as a reporting threshold in the statistical sense. Rather, it follows directly from our statistical procedure. Specifically, we used paired bootstrap testing with 1,000 redraws with replacement, consistent with our prior work and related studies. With 1,000 bootstrap samples, the smallest attainable p-value resolution is 0.001. We then applied Benjamini-Hochberg false discovery rate (FDR) correction within coherent families of related comparisons. For the adult-dataset comparisons in **Supplementary Table 1**, this means that multiple raw p-values of 0.001 can become 0.006 after correction across six adult datasets. This is why the same adjusted value appears repeatedly.
Thus, these values are not suspiciously uniform because of arbitrary rounding, but because several comparisons reached the same minimum raw bootstrap p-value and were then adjusted within the same FDR family. In the revised manuscript, we report the exact adjusted p-values in the tables, rather than replacing them with broad thresholds such as $p < 0.01$ or $p < 0.001$. In the running text, expressions such as $p \leq 0.006$ are used only as a compact way to summarize several comparisons that all support the same interpretation, while the exact per-comparison values are already provided in the table.
To make this clearer, we also revised the formatting and explanation in the manuscript. Small adjusted p-values are now reported to three decimal places where informative, for example 0.006, 0.003, and 0.001, whereas larger non-significant values are shown in compact journal-style format (two significant figures), for example 0.31 or 0.40. We have also clarified this point explicitly in the **Methods**.

The relevant **Methods** text now reads:
“Evaluation and statistical analysis
[…]
Statistical analysis was performed using Python 3.9 with NumPy 1.22, SciPy 1.10, and scikit-learn 1.2. Bootstrapping with 1,000 redraws was used to estimate means, standard deviations, and 95% confidence intervals (CI). For within-dataset comparisons between initialization strategies, backbones, resolutions, or adaptation regimes, paired bootstrap testing was performed on AUROC differences using identical

resampling across compared models. Formal cross-resolution testing between 512 × 512 and 1024 × 1024 was performed for all configurations evaluated at both resolutions. To account for multiple comparisons across related groups of tests, p-values were adjusted using the Benjamini-Hochberg false discovery rate (FDR) procedure, with statistical significance defined as FDR-adjusted as $p < 0.05$.
[…]"

Comment 10: *"Line 537: "learning rate of 10^-5" - was this optimized, or taken from prior work? A brief justification would strengthen reproducibility"*

Authors' Response and Action: We agree that the previous version did not sufficiently explain the choice of learning rate. In the revised Methods, we now clarify that the learning rate of 10^{-5} for full fine-tuning was not chosen arbitrarily. Rather, it was selected based on empirical testing of multiple candidate values in the present study, where it consistently provided the most stable convergence across datasets and model configurations. Although we did not perform a formal hyperparameter search, this value was also consistent with the range used successfully in our prior work on chest radiograph classification across several related settings and applications [1–6]. We have added this clarification to improve reproducibility.

The updated **Methods** segment now reads as follows:
"Main full fine-tuning benchmark
Universal experiments were performed at 224 × 224 and 512 × 512 pixels across all seven datasets. To probe whether the 512 × 512 advantage extended further, we additionally evaluated 1024 × 1024 pixels on three representative cohorts spanning pediatric and adult settings, namely Pedi-CXR, ChestX-ray14, and MIMIC-CXR.
All full fine-tuning experiments were optimized with AdamW using a learning rate of 10^{-5} and no weight decay. This learning rate was chosen based on empirical testing of multiple candidate values in the present study, where it provided the most stable convergence across datasets and model configurations, and it is also consistent with the range used in our prior work on chest radiograph classification. Across all experiments, data augmentation consisted of random horizontal flips and random rotations up to 7°. The loss function was binary weighted cross-entropy, with class weights set inversely proportional to the frequency of each label in the training set. All training was performed in full 32-bit floating point precision on a single NVIDIA L40S GPU with 48 GB VRAM and Intel Xeon Silver 4310 CPUs. For full fine-tuning, batch size was 128 at 224 × 224, 64 at 512 × 512, and 16 at 1024 × 1024. To preserve comparability across initialization strategies, backbones, and resolutions, all main benchmark models were trained under this shared optimization protocol rather than under separate per-configuration hyperparameter searches. […]"

Comment 11: *“Line 509: "scaled by the maximum" - maximum of what? Maximum pixel value in that image? Across the dataset? Clarify.”*

Authors’ Response and Action: We thank the Reviewer for noting this ambiguity. We have clarified the preprocessing description in the revised Methods. Intensity normalization was performed per image, not across the dataset. Specifically, each radiograph was first shifted so that its minimum pixel value became zero and was then scaled by its own maximum pixel value before clipping and conversion to 8-bit grayscale.

The revised **Methods** text now reads:

“To normalize intensity values, each image was shifted so that the minimum pixel value corresponded to zero, scaled by its maximum pixel value, and clipped to the valid range before conversion to 8-bit grayscale.”

Comment 12: *“Batch sizes: You mention they were "adapted to fit available GPU memory" but don't specify what they were. This affects reproducibility and interpretation.*
You use inverse-frequency weighting for the loss function. Did you explore other strategies (focal loss, oversampling)? Given the clinical importance of rare findings, this choice impacts interpretation.”

Authors’ Response and Action: We thank the Reviewer for these important points. We agree that the previous version did not provide enough detail on training batch sizes. In the revised Methods, we now specify the hardware and the exact batch sizes used for the main full fine-tuning experiments and for the LoRA experiments. Specifically, all training was performed in FP32 on a single NVIDIA L40S GPU with 48 GB VRAM and Intel Xeon Silver 4310 CPUs. For full fine-tuning, batch size was 128 at 224 × 224, 64 at 512 × 512, and 16 at 1024 × 1024. For the LoRA experiments at 512 × 512, batch size was 32. This clarification makes the resource constraints and optimization setup more reproducible.
Regarding class imbalance handling, we used binary weighted cross-entropy with inverse-frequency class weights, consistent with our prior work and common practice in large-scale multilabel chest radiograph classification. We did not perform a systematic comparison against alternative strategies such as focal loss or oversampling in the present study. We agree that this is relevant for interpretation, especially for rare findings, and we now state this more explicitly as a limitation in the **Discussion**. At the same time, we note that our main conclusions were supported not

only by AUROC but also by class-imbalance-aware metrics such as PR-AUC and mAP in the targeted follow-up analyses, which reduces the likelihood that the central findings are an artifact of this specific loss choice alone.

The updated **Methods** segment now reads as follows:

"Main full fine-tuning benchmark

Universal experiments were performed at 224 × 224 and 512 × 512 pixels across all seven datasets. To probe whether the 512 × 512 advantage extended further, we additionally evaluated 1024 × 1024 pixels on three representative cohorts spanning pediatric and adult settings, namely Pedi-CXR, ChestX-ray14, and MIMIC-CXR.
All full fine-tuning experiments were optimized with AdamW using a learning rate of 10^{-5} and no weight decay. This learning rate was chosen based on empirical testing of multiple candidate values in the present study, where it provided the most stable convergence across datasets and model configurations, and it is also consistent with the range used in our prior work on chest radiograph classification. Across all experiments, data augmentation consisted of random horizontal flips and random rotations up to 7°. The loss function was binary weighted cross-entropy, with class weights set inversely proportional to the frequency of each label in the training set. All training was performed in full 32-bit floating point precision on a single NVIDIA L40S GPU with 48 GB VRAM and Intel Xeon Silver 4310 CPUs. For full fine-tuning, batch size was 128 at 224 × 224, 64 at 512 × 512, and 16 at 1024 × 1024. To preserve comparability across initialization strategies, backbones, and resolutions, all main benchmark models were trained under this shared optimization protocol rather than under separate per-configuration hyperparameter searches.

Frozen-feature probing with DINOv3-7B

To test whether a much larger frozen foundation encoder could substitute for downstream adaptation, we additionally evaluated frozen representations from the DINOv3-7B model. In this setting, the backbone remained fully frozen and only a compact multilayer classification head, referred to as DINO-Net, was optimized. DINO-Net consisted of layer normalization applied to the 4096-dimensional backbone features, followed by a Gaussian error linear unit (GELU) activation and dropout with $p = 0.3$, a linear projection to a 512-dimensional embedding, a second dropout layer with $p = 0.3$, a second layer normalization, and a final linear mapping to the target label space. This head added approximately 2.1 million trainable parameters. Frozen-feature experiments were performed on all seven datasets at 224 × 224 and 512 × 512 pixels. Only the DINO-Net classifier was optimized, using AdamW with a learning rate of 10^{-4} and weight decay of 5×10^{-5}.

Parameter-efficient adaptation with LoRA

To test whether the observed backbone ranking depended on the use of full end-to-end fine-tuning, we performed a dedicated parameter-efficient adaptation analysis

using low-rank adaptation (LoRA)36. These experiments were conducted at 512 × 512 pixels on three adult datasets, namely ChestX-ray14, CheXpert, and MIMIC-CXR. We evaluated the same five backbone-initialization combinations as in the full fine-tuning benchmark: ViT-B/16 with ImageNet, DINOv2, or DINOv3 initialization, and ConvNeXt-B with ImageNet or DINOv3 initialization.

For ViT-B/16, LoRA adapters were applied to the attention projection layers, specifically query and value projections or their architecture-equivalent implementation. For ConvNeXt-B, adapters were applied to the pointwise projection layers within the ConvNeXt blocks, again using the corresponding implementation-specific module names. In all LoRA experiments, the classification head remained trainable. LoRA hyperparameters were fixed to rank 16, alpha 32, and dropout 0.05. Training used AdamW with learning rate 10^{-4} and batch size 32.

Synthetic label-noise experiment

To test whether gains from DINOv3 on weakly labeled adult datasets could be explained simply by improved tolerance to noisy supervision, we performed a controlled synthetic label-noise experiment on VinDr-CXR using ViT-B/16 at 512 × 512 pixels. This analysis compared the three ViT-B/16 initialization strategies, namely ImageNet, DINOv2, and DINOv3.

Noise was injected only into the training split, while the test set remained unchanged. Corruption was applied at predefined noise levels of 10%, 20%, 30%, and 40%, with the clean training labels serving as the 0% reference. Noise was introduced only for the 10 abnormal labels in the VinDr-CXR setup, excluding the “No finding” label. We used an asymmetric corruption scheme intended to better approximate weak-label errors: positive labels were flipped to negative at the specified noise rate, whereas negative labels were flipped to positive at one quarter of that rate. After corruption of the abnormal labels, the “No finding” label was recomputed on the training set so that it remained mutually consistent with the pathology labels. Test labels were never modified.

External validation across institutions

To determine whether the main benchmark findings extended beyond within-dataset train-test splits, we performed an external validation experiment using a harmonized seven-label setting comprising cardiomegaly, pleural effusion, pneumonia, atelectasis, no finding, consolidation, and pneumothorax. Models were trained on MIMIC-CXR and evaluated both on the internal MIMIC-CXR test set and on the fully held-out external datasets ChestX-ray14 and CheXpert. External validation was performed at 224 × 224 and 512 × 512 pixels for the same five backbone-initialization combinations used in the main benchmark.

Computational efficiency analysis

To place performance differences in a practical context, computational efficiency was assessed for all full fine-tuning experiments by measuring both per-epoch training time and total wall-clock time to convergence on the same hardware platform, namely a single NVIDIA L40S GPU with 48 GB VRAM and Intel Xeon Silver 4310 CPUs, using full 32-bit floating point precision throughout. This analysis was intended as a practical efficiency comparison within the present benchmark rather than as a formal systems-level profiling study; accordingly, it did not include inference latency, GPU memory traces, or FLOPs.

Per-epoch training time was recorded for each evaluated full fine-tuning configuration, defined by dataset, initialization, backbone, and image resolution. Across the universal 224 × 224 and 512 × 512 experiments, this yielded measurements for all seven datasets, three initialization strategies for ViT-B/16, and two initialization strategies for ConvNeXt-B. For the targeted 1024 × 1024 setting, measurements were obtained only for the three representative cohorts evaluated at that resolution, namely Pedi-CXR, ChestX-ray14, and MIMIC-CXR. For each configuration, we also recorded the number of epochs required to convergence, allowing computation of total wall-clock training time.

Relative computational cost was then summarized as ratios across resolution transitions, particularly for 224 × 224 to 512 × 512 and 512 × 512 to 1024 × 1024. These ratios were aggregated across the relevant sets of configurations and reported descriptively using means, standard deviations, medians, and ranges. This analysis allowed us to relate performance changes directly to measured training cost under a fixed implementation and hardware setting."

Moreover, the following has been added to the limitations part in **Discussion**:

"[…] In addition, class imbalance was handled using inverse-frequency weighting, but we did not systematically compare alternative strategies such as focal loss, oversampling, or other cost-sensitive objectives, which may be relevant particularly for rare findings."

Comment 13: *"ConvNeXt-B's superiority over ViT-B is interesting, but could this be partially explained by ConvNeXt's better handling of higher resolutions due to its convolutional nature? A brief mechanistic discussion would add insight."*

Authors' Response and Action: We thank the Reviewer for this insightful point. We agree that the backbone difference benefits from a brief mechanistic interpretation. In the revised manuscript, we now discuss this explicitly in the **Discussion**. We do not claim to prove the mechanism directly, but we now note that ConvNeXt-B's convolutional inductive biases and hierarchical local feature aggregation may make it better suited to exploit high-resolution chest radiographs, where diagnostically relevant

information is often spatially local and boundary-sensitive. We also note that this interpretation is consistent with our empirical findings: the ConvNeXt-B advantage became most apparent at 512 × 512 pixels, persisted under parameter-efficient adaptation, incurred a smaller computational penalty than ViT-B/16 when scaling to 1024 × 1024, and was the only backbone to show significant gains at 1024 × 1024 in MIMIC-CXR. We have therefore added a more explicit mechanistic discussion while keeping the interpretation appropriately cautious.

The revised **Discussion** text now reads:
"The backbone findings were also robust to changes in adaptation regime. Under full fine-tuning, ConvNeXt-B consistently outperformed ViT-B/16, particularly at 512 × 512 pixels. Parameter-efficient adaptation, implemented here with LoRA, did not narrow this gap. Instead, it reduced performance in all evaluated settings and generally affected ViT-B/16 more strongly than ConvNeXt-B, especially for DINOv3 initialization. This suggests that the ConvNeXt advantage cannot be explained simply by the use of full end-to-end fine-tuning. A plausible interpretation is that convolutional inductive biases and hierarchical local feature aggregation remain advantageous in chest radiograph analysis, particularly when diagnostically relevant information is spatially local and when high-resolution inputs emphasize subtle boundaries and fine structures. This interpretation is also consistent with our scaling results, in which ConvNeXt-B incurred a smaller computational penalty than ViT-B/16 at higher resolution and was the only backbone to show significant gains at 1024 × 1024 in MIMIC-CXR. While this does not prove that convolutional backbones are universally superior to transformers in radiology, it does show that, at base scale and under both full and parameter-efficient adaptation, ConvNeXt-B provided the more reliable transfer behavior in our benchmark."

Comment 14: *"All seven datasets were used for training and testing (with splits). Did you consider holding out an entire dataset for external validation to test true generalization?"*

Authors' Response and Action: We thank the Reviewer for this important suggestion. We agree that true dataset-level external validation is essential to test whether the observed ranking of initialization strategies generalizes beyond within-dataset splits. In the revised manuscript, we therefore added a dedicated external-validation experiment in which models were trained on MIMIC-CXR and then evaluated on the fully held-out external datasets ChestX-ray14 and CheXpert using a harmonized seven-label setting. This new analysis is presented in **Table 4** and **Figure 6a-d** and discussed explicitly in both the **Results** and **Discussion**.
The new external-validation analysis showed that external AUROC drops were modest, ranging from 0.9 to 2.7 percentage points at 512 × 512, and that the relative

ranking of configurations was largely preserved under domain shift. In particular, DINOv3 remained the strongest initialization at 512 × 512 for both ViT-B/16 and ConvNeXt-B on both external target datasets, and the resolution advantage of 512 × 512 over 224 × 224 also persisted externally. Class-imbalance-aware metrics supported the same conclusion, with PR-AUC and mAP closely tracking AUROC across configurations.
At the same time, we state explicitly in the manuscript that this external-validation design is still not a full all-to-all cross-dataset study, since it uses one training source and a harmonized seven-label subset. This is now acknowledged in the limitations part of **Discussion** section.

The new **Results** subsection reads as follows:
"DINOv3 generalizes across institutions under external validation

To test whether the main benchmark findings extended beyond within-dataset splits, we trained models on MIMIC-CXR and evaluated them externally on ChestX-ray14 and CheXpert using a harmonized seven-label setting comprising cardiomegaly, pleural effusion, pneumonia, atelectasis, no finding, consolidation, and pneumothorax (**Figure 6a-d, Table 4**). Across all five evaluated configurations, external performance remained close to the corresponding internal test performance, with AUROC drops of 0.9 to 2.7 percentage points at 512 × 512. The smallest external drop was observed for ConvNeXt-B with DINOv3 on ChestX-ray14, from 79.3 ± 0.2 internally to 78.3 ± 0.2 externally, corresponding to a gap of 1.0 ± 0.3. On CheXpert, the external gaps were somewhat larger but remained modest overall, ranging from 2.3 ± 0.3 to 2.7 ± 0.3. Importantly, the relative ranking of configurations was largely preserved under domain shift. At 512 × 512, DINOv3 remained the strongest initialization for both backbones on both external target datasets. For ViT-B/16, DINOv3 reached 78.0 ± 0.2 on ChestX-ray14 and 80.6 ± 0.2 on CheXpert, exceeding both ImageNet and DINOv2. The same pattern held for ConvNeXt-B, where DINOv3 achieved the highest external AUROC on ChestX-ray14 (78.3 ± 0.2) and CheXpert (80.7 ± 0.2). Thus, the DINOv3 advantage observed in the internal benchmark was not lost when transferring across institutions. The resolution effect also persisted externally. Across both target datasets and all configurations, moving from 224 × 224 to 512 × 512 improved external AUROC by 0.8 to 1.5 percentage points (**Figure 6b, Table 4**). For example, ViT-B with DINOv3 improved from 76.5 ± 0.2 to 78.0 ± 0.2 on ChestX-ray14 and from 79.2 ± 0.2 to 80.6 ± 0.2 on CheXpert. ConvNeXt-B with DINOv3 showed a similar pattern, increasing from 76.8 ± 0.2 to 78.3 ± 0.2 on ChestX-ray14 and from 79.4 ± 0.2 to 80.7 ± 0.2 on CheXpert. This indicates that the 512 × 512 advantage was not specific to in-domain evaluation. Class-imbalance-aware metrics supported the same conclusion. External PR-AUC and mAP closely tracked AUROC across configurations, with DINOv3 again ranking highest at 512 × 512 for both backbones and both target datasets. For example, ConvNeXt-B with DINOv3 achieved the best external PR-AUC and mAP on both

ChestX-ray14 (35.1 ± 0.3 and 35.2 ± 0.3) and CheXpert (42.4 ± 0.3 and 42.4 ± 0.3), and the external AUROC-mAP relationship remained strongly aligned across institutions (**Figure 6d**). These findings show that the main adult benchmark result, namely the advantage of DINOv3 at 512 × 512, remained visible under external validation rather than being confined to internal splits.”

The relevant **Discussion** paragraph:
“At the same time, the external-validation experiment shows that the main adult finding is not merely an artifact of within-dataset splits. When models were trained on MIMIC-CXR and evaluated on the fully held-out external datasets ChestX-ray14 and CheXpert using a harmonized seven-label setting, external AUROC drops were modest and the relative ordering of configurations was largely preserved. DINOv3 remained the strongest initialization at 512 × 512 pixels for both backbones on both external target datasets, and the external advantage of 512 × 512 over 224 × 224 also persisted. Importantly, PR-AUC and mAP tracked AUROC closely in these analyses, arguing against the possibility that the observed ranking depended mainly on the choice of metric. Together, the noise and external-validation results refine the overall interpretation: DINOv3 generalizes across institutions, but its gains are better understood as improved transferable representation quality than as broad robustness to corrupted supervision. At the same time, this experiment used a single training source and a harmonized label subset rather than a full all-to-all cross-dataset design, so broader external generalization should still be tested in future work.”

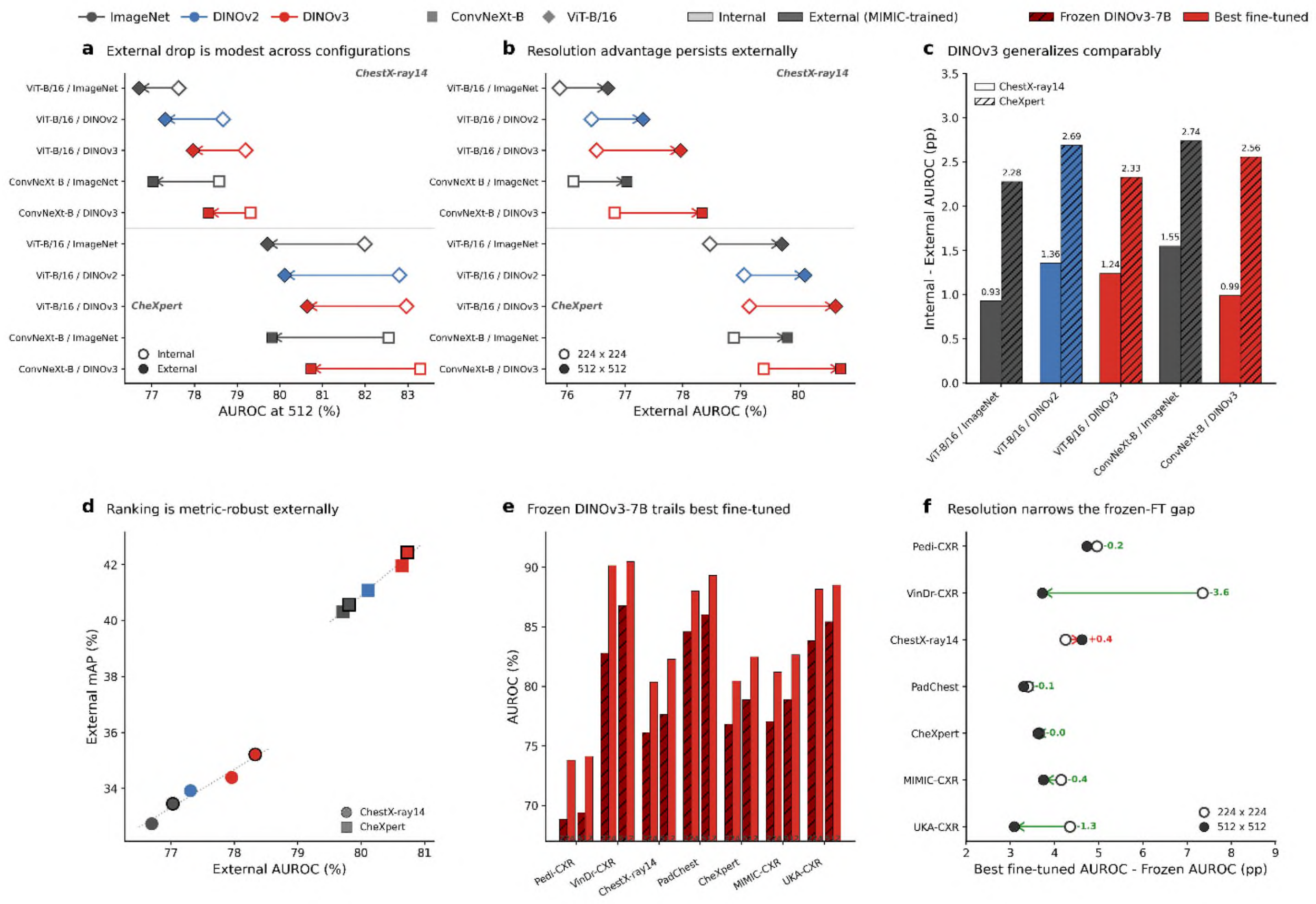

Figure 6: External validation and frozen feature comparison.

Comment 15: *"Add a limitations paragraph specifically addressing:*
Why 1024×1024 wasn't tested exhaustively
Add a limitations paragraph specifically addressing:
The confounding of projection type (AP vs PA) with dataset"
Add a limitations paragraph specifically addressing:
Lack of prospective clinical validation"

Authors' Response and Action: We thank the Reviewer for these important suggestions. We agree that these points should be stated more explicitly in the Limitations section. In the revised manuscript, we now clarify three issues. First, the 1024 × 1024 analysis was intentionally targeted rather than exhaustive because of the substantial computational burden of full high-resolution training across all backbone-initialization-dataset combinations, and it was designed as a focused probe of whether scaling beyond 512 × 512 yielded meaningful additional benefit. Second, although we restricted all analyses to AP and PA radiographs, the relative distribution of AP vs. PA projections differs across datasets, so projection type remains partially confounded with dataset and may influence apparent cross-dataset performance differences. Third, we now state explicitly that this work remains retrospective and benchmark-based,

without prospective clinical validation or workflow testing. We agree that these are important boundaries on the interpretation of the present findings.

The revised **Limitations** paragraph of **Discussion** now reads:
"This study has several limitations. First, although we included seven datasets from multiple countries and both public and internal sources, the main benchmark remained limited to AP and PA chest radiographs and to classification tasks; the relevance of these conclusions to lateral views, localization, segmentation, or report generation remains to be established. Indeed, because DINOv3 is designed to preserve dense patch-level structure and our strongest gains were observed for small focal and boundary-dependent findings, extending this analysis to localization and segmentation tasks represents a particularly important future direction. In addition, the relative distribution of AP and PA projections differed across datasets, so projection type remained partially confounded with dataset and may have contributed to apparent cross-dataset differences in performance. Second, the 1024 × 1024 analysis was deliberately targeted rather than exhaustive, and only three cohorts were evaluated at that resolution. This design was chosen because of the substantial computational burden of full high-resolution training and was intended as a focused test of whether scaling beyond 512 × 512 yielded meaningful additional benefit under the present training setup. Third, the backbone comparison under parameter-efficient adaptation was restricted to LoRA and to three adult datasets, so broader conclusions about all parameter-efficient fine-tuning strategies or all model scales would be premature. Fourth, the external-validation analysis used a harmonized seven-label subset and one training source, not a full all-to-all cross-dataset design. Fifth, the frozen-feature comparison was restricted to a natural-image-pretrained DINOv3-7B encoder and did not include frozen medical-domain self-supervised encoders, including emerging DINOv3-based medical adaptations such as MedDINOv3, so it does not by itself distinguish domain mismatch from a more general limitation of frozen representations. Sixth, the label-noise experiment used synthetic symmetric corruption in a single dataset, which provides a controlled stress test but cannot capture all properties of real report-derived labeling noise. In addition, class imbalance was handled using inverse-frequency weighting, but we did not systematically compare alternative strategies such as focal loss, oversampling, or other cost-sensitive objectives, which may be relevant particularly for rare findings. Finally, although class-imbalance-aware metrics supported the main follow-up analyses, AUROC remained the primary metric for the benchmark as a whole, and the study did not include prospective clinical validation or workflow-level evaluation."

Comment 16: *“Expand on clinical implementation: What specific thresholds or operating points would you recommend? The AUROC improvements are modest (0.5-1.0 points) - when does this translate to clinical utility?”*

Authors’ Response and Action: We thank the Reviewer for this important point. We agree that AUROC gains of 0.5 to 1.0 percentage points need to be interpreted in terms of where they occur and how a system would be deployed clinically. In the revised **Discussion**, we now make this more explicit. Specifically, we clarify that we do not propose a single universal operating threshold from the present benchmark, because threshold selection depends on the intended clinical use case, local disease prevalence, and the relative cost of false-negative vs. false-positive decisions. Instead, we now emphasize a more practical recommendation: for adult chest radiograph classification, the most suitable technical operating point in our benchmark was a fully adapted ConvNeXt-B model at 512 × 512 pixels, while the downstream decision threshold should be selected locally according to the deployment objective. For example, triage or emergency screening would typically favor higher sensitivity, whereas workflow-prioritization or rule-out applications may require a different trade-off. We also clarify that the observed AUROC gains are most likely to matter when they are concentrated in subtle, clinically consequential findings such as pneumothorax, small nodules, or faint focal opacities, rather than in already easy large-structure findings.

The revised **Discussion** text now reads:
“From a clinical perspective, the magnitude of the gains in adult cohorts was modest in absolute AUROC terms, often around 0.5 to 1.0 points, but the distribution of those gains matters. Improvements concentrated in small focal lesions and boundary-dependent findings are more likely to be relevant to emergency, inpatient, and triage settings than improvements on already easy large-structure labels. A system that better resolves subtle pneumothorax, small nodules, or faint focal opacities can be clinically valuable even when the mean AUROC shift seems small. We therefore do not propose a single universal probability threshold from the present benchmark, because the appropriate operating point depends on the intended clinical task, local prevalence, and the relative cost of false-negative and false-positive decisions. In practice, triage-oriented use cases would typically favor higher sensitivity, whereas workflow-prioritization or rule-out settings may require a different trade-off. Our results therefore support a pragmatic recommendation at the model-selection level: when using off-the-shelf self-supervised visual initialization for adult chest radiograph classification, prioritize 512 × 512 inputs and a ConvNeXt-B backbone, and treat further scaling in size or resolution cautiously unless a clear task-specific benefit is shown.”

Comment 17: *"Future directions: You mention segmentation and localization briefly (line 400-401). Given that DINOv3 preserves patch-level features, this seems like a natural extension worth emphasizing."*

Authors' Response and Action: We thank the Reviewer for this suggestion. We agree that segmentation and localization are a natural next step, particularly because DINOv3 was designed to preserve dense patch-level structure. In the revised **Discussion**, we now emphasize this point more explicitly. Specifically, we clarify that, although the present study is limited to classification, the observed resolution dependence and the concentration of gains in small focal and boundary-dependent findings make localization- and segmentation-oriented tasks especially relevant future directions. We have therefore expanded the limitations/future-directions paragraph accordingly.

The revised **Discussion** text now reads:
"**Discussion**
[…]
This study has several limitations. First, although we included seven datasets from multiple countries and both public and internal sources, the main benchmark remained limited to AP and PA chest radiographs and to classification tasks; the relevance of these conclusions to lateral views, localization, segmentation, or report generation remains to be established. Indeed, because DINOv3 is designed to preserve dense patch-level structure and our strongest gains were observed for small focal and boundary-dependent findings, extending this analysis to localization and segmentation tasks represents a particularly important future direction. […]"

Comment 18: *"Table 2: Consider adding a column showing computational cost ratios"*

Authors' Response and Action: We thank the Reviewer for this helpful suggestion. Rather than adding computational ratios directly into **Table 2**, which is intended to remain the core performance benchmark at 224 × 224 and 512 × 512, we addressed this point by adding a dedicated high-resolution and efficiency table (**Table 3**) together with a broader computational scaling summary (**Supplementary Table 3**) and explicit visualization in **Figure 2h,i.** This allows us to present the cost analysis in a clearer and more interpretable way than a single additional column in **Table 2.** Please also refer to our joint response to **Comments 2** and **3**.

Comment 19: *“Add a figure showing the resolution-AUROC relationship across datasets more directly (currently spread across multiple figures)”*

Authors’ Response and Action: We thank the Reviewer for this valuable suggestion. This point has been addressed in the revised manuscript through the reorganization of **Figure 2** and the addition of **Figure 3**. In particular, **Figure 2c,d,f** now visualize the AUROC change from 224 × 224 to 512 × 512 directly across datasets and configurations, while **Figure 3a,b** summarize the same resolution-performance relationship at the cohort level and highlight the distinct pediatric behavior. We believe this revised figure structure now presents the resolution-AUROC relationship much more directly than in the previous version.

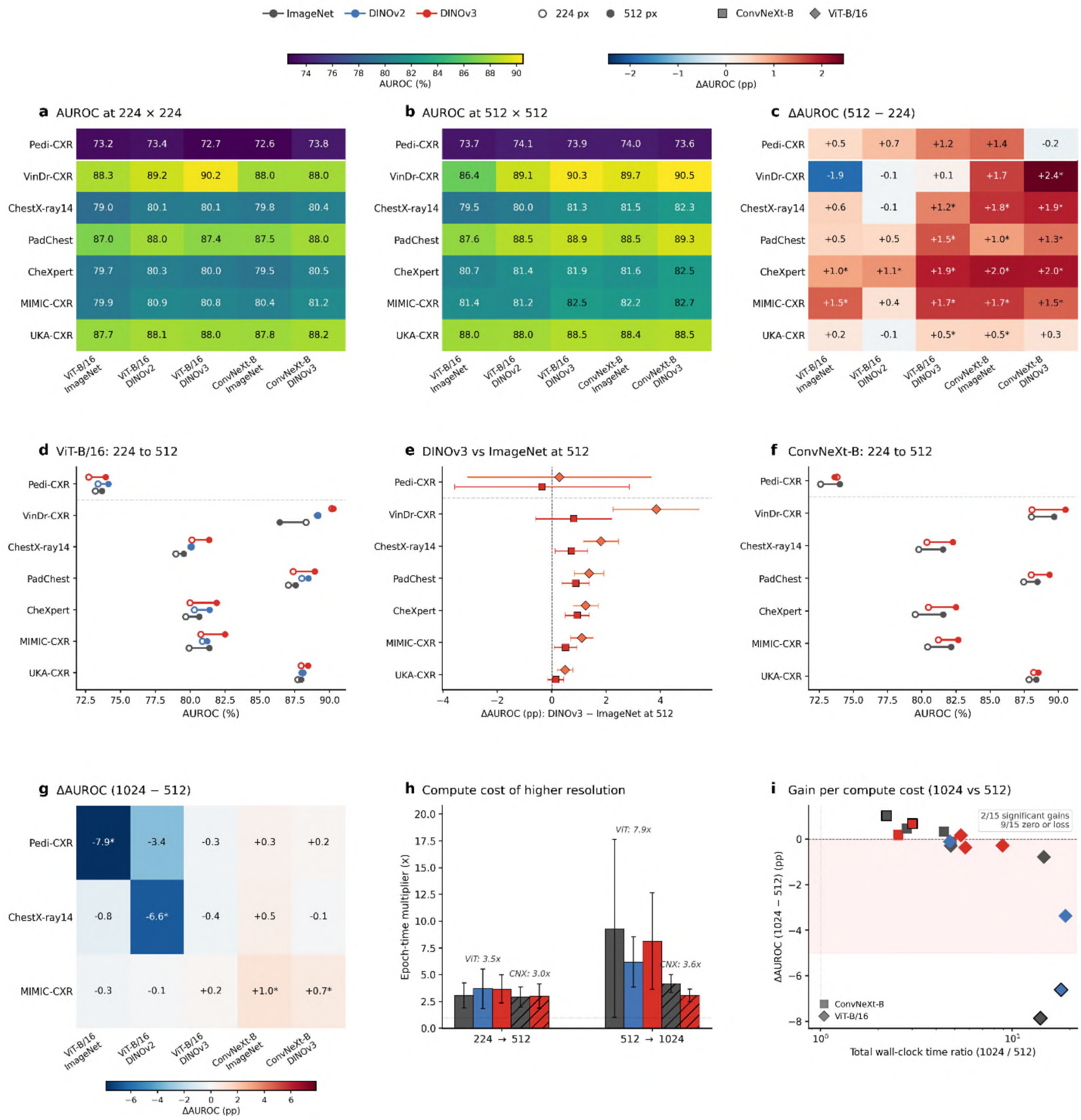

Figure 2: Resolution scaling and computational cost-benefit analysis.

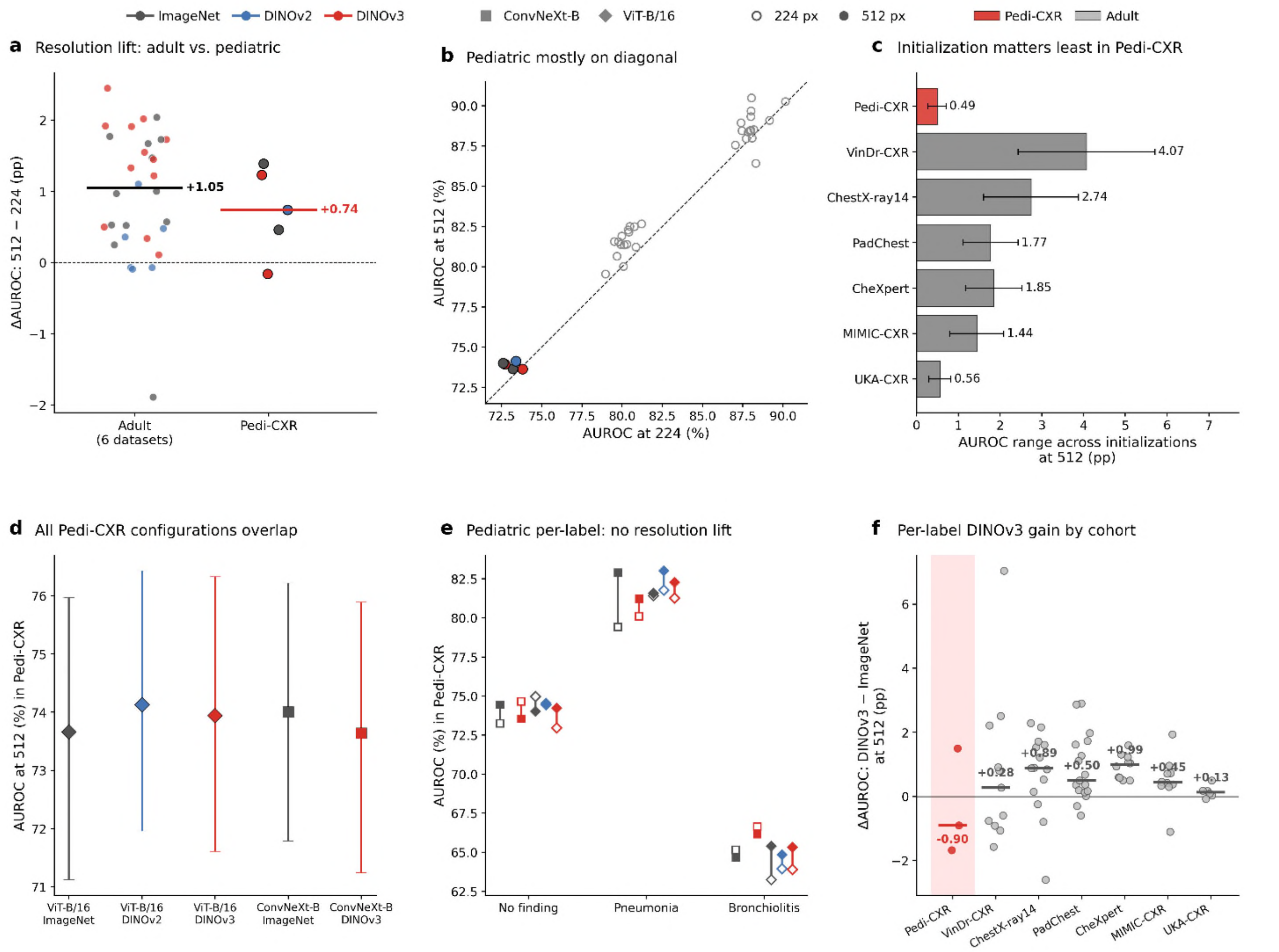

Figure 3: The pediatric cohort shows a distinct transfer pattern that does not follow the adult resolution-scaling behavior. Results are shown for all seven datasets: Pedi-CXR (training n=7,728; test n=1,397), VinDr-CXR (training n=15,000; test n=3,000), ChestX-ray14 (training n=77,870; validation n=8,654; test n=25,596), PadChest (training n = 79,697; validation n=8,783; test n=22,045), CheXpert (training n=115,449; validation n=13,098; test n=29,318), MIMIC-CXR (training n=153,255; validation n=18,139; test n=43,793), and UKA-CXR (training n=137,902; validation n=15,353; test n=40,106). **a** Distribution of AUROC change when increasing resolution from 224 to 512 pixels for every backbone-initialization combination, separated by adult cohorts (6 datasets, 30 configurations) and Pedi-CXR (5 configurations). Each point represents one configuration within one dataset. Horizontal bars mark the median per group, annotated in bold. Adult configurations show a consistently positive resolution lift; Pedi-CXR configurations cluster around zero. **b** AUROC at 224 pixels against AUROC at 512 pixels for every configuration across all seven datasets. The dashed line denotes equality. Adult configurations (grey) sit above the line; pediatric configurations (colored by initialization) sit on it. **c** Range of mean AUROC across the five initialization-backbone configurations at 512 pixels for each dataset, with standard deviation error bars. Pedi-CXR exhibits the narrowest range (0.49 pp), indicating that initialization choice has minimal influence on pediatric performance. **d** Mean AUROC at 512 pixels with 95% bootstrap confidence intervals for all five Pedi-CXR configurations. All intervals overlap, confirming that no initialization or backbone offers a statistically distinguishable advantage. **e** Per-label dumbbell plot of the 224-to-512 AUROC shift for each of the three pediatric labels (Any finding, Pneumonia, Bronchiolitis). Open markers denote 224-pixel performance; filled markers denote 512-pixel performance. Colors denote initialization; marker shapes denote backbone (squares for ConvNeXt-B, diamonds for ViT-B/16). No label shows a consistent resolution-driven gain. **f** Distribution of per-label AUROC differences (DINOv3 minus ImageNet at 512, ConvNeXt-B) across all seven datasets. Each point represents one pathology label; horizontal bars mark the median. Pedi-CXR is the only cohort with a negative median, confirming that the DINOv3 advantage seen at the label level in adult cohorts does not transfer to the pediatric cohort.

Comment 20: *"Include example images demonstrating the types of findings that benefit most from 512×512 resolution"*

Authors' Response and Action: We thank the Reviewer for this helpful suggestion. In the revised manuscript, we have now added a new supplementary figure (**Supplementary Figure 8**) that provides representative example radiographs illustrating the types of findings for which the largest resolution-related gains were observed. Specifically, the new figure visually highlights small focal lesions and boundary-dependent abnormalities, which were also the groups showing the largest average improvements in our quantitative cross-dataset summary (**Supplementary Table 2**). We emphasize that this new figure is intended as an illustrative complement to the quantitative analysis, rather than as a separate source of evidence.

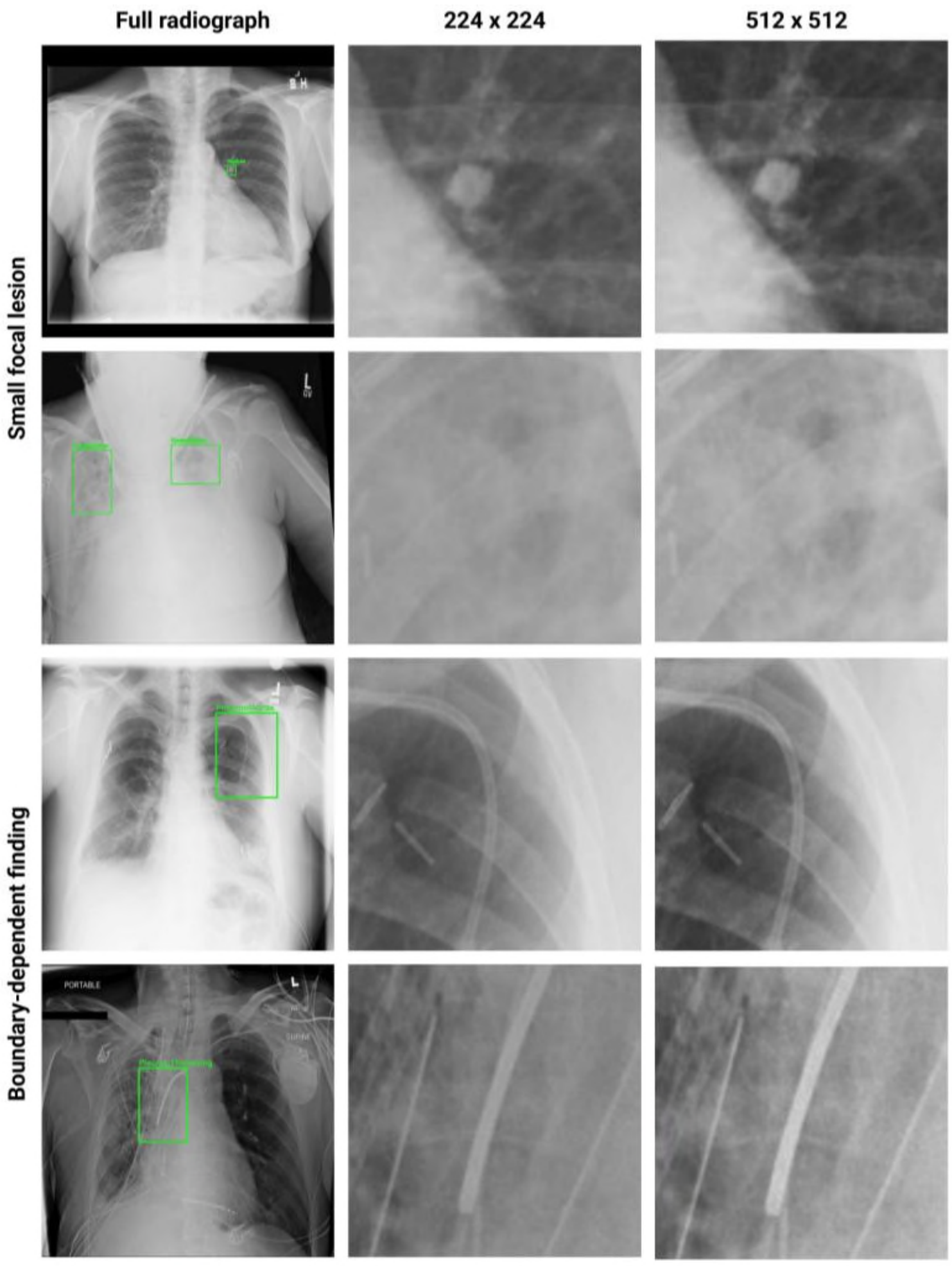

Supplementary Figure 8: Illustrative adult chest radiographs highlighting regions corresponding to findings with the greatest apparent benefit from higher input resolution. Shown are four representative examples from the adult ChestX-ray14 cohort, selected to visualize regions corresponding to small focal and boundary-dependent findings, the two groups that showed the largest average resolution-related gains in the quantitative cross-dataset summary (**Supplementary Table 2**). The left column shows the full radiograph with a manually chosen region of interest and the associated dataset label. The middle and right columns show the corresponding region after standard preprocessing and resizing to 224 × 224 and 512 × 512 pixels, respectively. The cropped regions from the resized images were enlarged to a common display size for visual comparison. Across these illustrative examples, the 512 × 512 versions preserve finer local structure and boundary detail more clearly than the 224 × 224 versions. This figure is intended as a qualitative complement to the quantitative analysis, not as a model-explanation figure and not as a separate performance experiment.

Comment 21: *"Line 89: "intelligence (AI)" - AI already defined in abstract"*

Authors' Response and Action: We thank the Reviewer for noting this stylistic point. In the current manuscript, we retained the definition of artificial intelligence (AI) in the main text, because abbreviations are commonly defined independently in the abstract and in the main manuscript body, which are often read as separate sections. We therefore did not regard this as an error requiring substantive revision. If preferred at the proof stage, this can be harmonized.

Comment 22: *"Line 293: "dataset-level trends reflect differences in annotation quality more than dataset size" - strong claim that needs either a citation or more supporting evidence"*

Authors' Response and Action: We thank the Reviewer for this important point. We agree that this statement was too strong as written. In the revised manuscript, this sentence is no longer present because the corresponding section was removed during the major restructuring of the **Results** and **Discussion**. We therefore no longer make this claim in the current version of the paper. Instead, the revised manuscript addresses related issues more cautiously through the dedicated synthetic label-noise experiment and the discussion of label provenance and cohort differences, without attributing dataset-level trends primarily to annotation quality over dataset size.

Comment 23: *"Line 325: formatting issue with citation "straightforwardly extend to medical imaging^25"*

Authors' Response and Action: We thank the Reviewer for noting this formatting issue. The citation formatting has been corrected in the revised manuscript.

Reply to Reviewer #2
COMMSMED-25-2461

Title:
Resolution-dependent self-supervised transfer in chest radiograph classification

General Comment: *"This manuscript presents a large-scale benchmark study evaluating DINOv3 for chest radiograph classification across multiple datasets, resolutions, and backbone architectures. The authors systematically compare ImageNet, DINOv2, and DINOv3 initializations on ViT-B/16 and ConvNeXt-B backbones and report that DINOv3, particularly when paired with ConvNeXt-B at 512×512 resolution, achieves the strongest overall performance. The scale of the evaluation and the focus on resolution effects are valuable contributions. While the experimental effort is substantial and the results are generally well presented, I have several concerns regarding the experimental design and the interpretation of the findings, which should be addressed."*

> **Authors' Response and Action**: We thank the Reviewer for the careful reading of our manuscript and for recognizing both the scale of the benchmark and the value of the resolution-focused analysis. We also appreciate the Reviewer's concerns regarding experimental design and interpretation. In response, we substantially revised the manuscript. We address each of the Reviewer's specific concerns in detail below.

Comment 1: *"All main experiments rely exclusively on full end-to-end fine-tuning of the backbone, which may systematically favor ConvNeXt over ViT architectures. Convolutional models are generally more stable and data-efficient under full fine-tuning, whereas vision transformers often benefit from parameter-efficient fine-tuning (PEFT) strategies such as adapters or LoRA. Consequently, the conclusion that ConvNeXt-B "consistently outperforms" ViT-B may be partly attributable to the chosen optimization regime rather than an inherent architectural advantage. Incorporating PEFT-based baselines, or explicitly acknowledging this limitation, would strengthen the validity of the backbone comparison."*

> **Authors' Response and Action**: We thank the Reviewer for this important point. We agree that, in the original version, the backbone comparison could have been interpreted as partly entangled with the use of full end-to-end fine-tuning. To address this directly, we added a dedicated parameter-efficient adaptation analysis in the revised manuscript, implemented with LoRA at 512 × 512 on three adult datasets: ChestX-ray14, CheXpert, and MIMIC-CXR. This new experiment was designed specifically to test whether the observed ConvNeXt-B advantage could be explained mainly by the optimization regime rather than by backbone behavior itself.

The new analysis showed that replacing full fine-tuning with LoRA did not rescue ViT-B/16 or narrow the backbone gap. Instead, LoRA reduced performance in all evaluated settings, and ConvNeXt-B remained superior to ViT-B/16 in five of six backbone-initialization pairs. For DINOv3 specifically, the ConvNeXt-B minus ViT-B/16 difference increased from 1.0 to 3.2 percentage points in ChestX-ray14 and from 0.2 to 2.2 percentage points in MIMIC-CXR, while remaining positive in CheXpert. Moreover, the LoRA-associated performance drop was larger for ViT-B/16 than for ConvNeXt-B in all three datasets for DINOv3. These results indicate that the ConvNeXt-B advantage cannot be attributed simply to having used full fine-tuning in the main benchmark.
We also now state this more explicitly in the limitations of **Discussion**. At the same time, we acknowledge that the PEFT comparison in the present study was restricted to one strategy, namely LoRA, and to three adult datasets, so broader claims about all PEFT methods or all transformer scales would still be premature.

The new **Results** subsection reads:
"ConvNeXt-B remains superior to ViT-B/16 under both full and parameter-efficient adaptation
Under full fine-tuning, ConvNeXt-B consistently outperformed ViT-B/16 across adult datasets and resolutions (**Table 2**), with the gap most apparent at 512 × 512 pixels. For example, with DINOv3 initialization at 512 × 512, ConvNeXt-B reached 82.7 ± 0.2 vs. 82.5 ± 0.1 in MIMIC-CXR, 82.3 ± 0.2 vs. 81.3 ± 0.2 in ChestX-ray14, and 82.5 ± 0.1 vs. 81.9 ± 0.2 in CheXpert. Thus, the main benchmark already indicated a stable backbone advantage under standard end-to-end optimization. We then asked whether this result could be explained simply by the use of full fine-tuning by replacing it with a parameter-efficient adaptation regime, implemented here with LoRA (**Figure 4a-c, Supplementary Table 4**). Across all evaluated dataset-configuration pairs, LoRA reduced AUROC relative to full fine-tuning. The magnitude of this loss ranged from 1.3 ± 0.3 to 5.1 ± 0.3 percentage points for ViT-B/16 and from 2.1 ± 0.2 to 4.1 ± 0.3 percentage points for ConvNeXt-B. For DINOv3 specifically, the drop under LoRA was larger for ViT-B/16 than for ConvNeXt-B in all three datasets: 5.1 ± 0.3 vs. 2.8 ± 0.3 in ChestX-ray14, 4.3 ± 0.2 vs. 3.3 ± 0.2 in CheXpert, and 4.1 ± 0.2 vs. 2.1 ± 0.2 in MIMIC-CXR. Thus, reduced adaptation capacity did not preferentially benefit ViT-B/16.
Accordingly, the backbone gap did not narrow under parameter-efficient adaptation. Instead, ConvNeXt-B remained superior to ViT-B/16 in five of six backbone-initialization pairs, and the gap widened in several cases (**Figure 4c**). For DINOv3, the ConvNeXt-B minus ViT-B/16 difference increased from 1.0 to 3.2 percentage points in ChestX-ray14 and from 0.2 to 2.2 percentage points in MIMIC-CXR, while remaining positive in CheXpert (0.6 to 1.6 percentage points). The same qualitative pattern held for ImageNet initialization. These findings indicate that the ConvNeXt advantage cannot be attributed merely to having used full end-to-end fine-tuning in the main benchmark.

The initialization story also remained broadly stable. Under parameter-efficient adaptation, DINOv3 retained an advantage over ImageNet for ConvNeXt-B in all three datasets and for ViT-B/16 in MIMIC-CXR, but this advantage weakened or disappeared for ViT-B/16 in ChestX-ray14 and CheXpert (**Figure 4d**). This suggests that ViT-B/16 was more sensitive to the restricted adaptation regime, whereas ConvNeXt-B preserved both its stronger absolute performance and its relative benefit from DINOv3 initialization.

At the label level, parameter-efficient adaptation produced heterogeneous but predominantly negative changes, with median per-label AUROC shifts of about -2.8 percentage points in MIMIC-CXR, -2.8 percentage points in ChestX-ray14, and -2.6 percentage points in CheXpert (**Figure 4e**). Importantly, these shifts did not alter the overall ranking of configurations. Class-imbalance-aware evaluation supported the same conclusion: under LoRA, AUROC and mAP remained strongly aligned across configurations (Spearman $\rho = 1.00$ for MIMIC-CXR, $\rho = 1.00$ for ChestX-ray14, and $\rho = 0.90$ for CheXpert; all $p \leq 0.037$), indicating that the backbone comparison was not an artifact of AUROC alone (**Figure 4f**). The superiority of ConvNeXt-B over ViT-B/16 cannot be explained simply by the use of full fine-tuning. Instead, it persisted under both full and parameter-efficient adaptation, supporting a genuine backbone-level difference in transfer behavior for chest radiograph classification."

The relevant **Discussion** text now states:

"The backbone findings were also robust to changes in adaptation regime. Under full fine-tuning, ConvNeXt-B consistently outperformed ViT-B/16, particularly at 512 × 512 pixels. Parameter-efficient adaptation, implemented here with LoRA, did not narrow this gap. Instead, it reduced performance in all evaluated settings and generally affected ViT-B/16 more strongly than ConvNeXt-B, especially for DINOv3 initialization. This suggests that the ConvNeXt advantage cannot be explained simply by the use of full end-to-end fine-tuning. A plausible interpretation is that convolutional inductive biases and hierarchical local feature aggregation remain advantageous in chest radiograph analysis, particularly when diagnostically relevant information is spatially local and when high-resolution inputs emphasize subtle boundaries and fine structures. This interpretation is also consistent with our scaling results, in which ConvNeXt-B incurred a smaller computational penalty than ViT-B/16 at higher resolution and was the only backbone to show significant gains at 1024 × 1024 in MIMIC-CXR. While this does not prove that convolutional backbones are universally superior to transformers in radiology, it does show that, at base scale and under both full and parameter-efficient adaptation, ConvNeXt-B provided the more reliable transfer behavior in our benchmark."

And the **Limitations** part of **Discussion** section now includes:

“Third, the backbone comparison under parameter-efficient adaptation was restricted to LoRA and to three adult datasets, so broader conclusions about all parameter-efficient fine-tuning strategies or all model scales would be premature.”

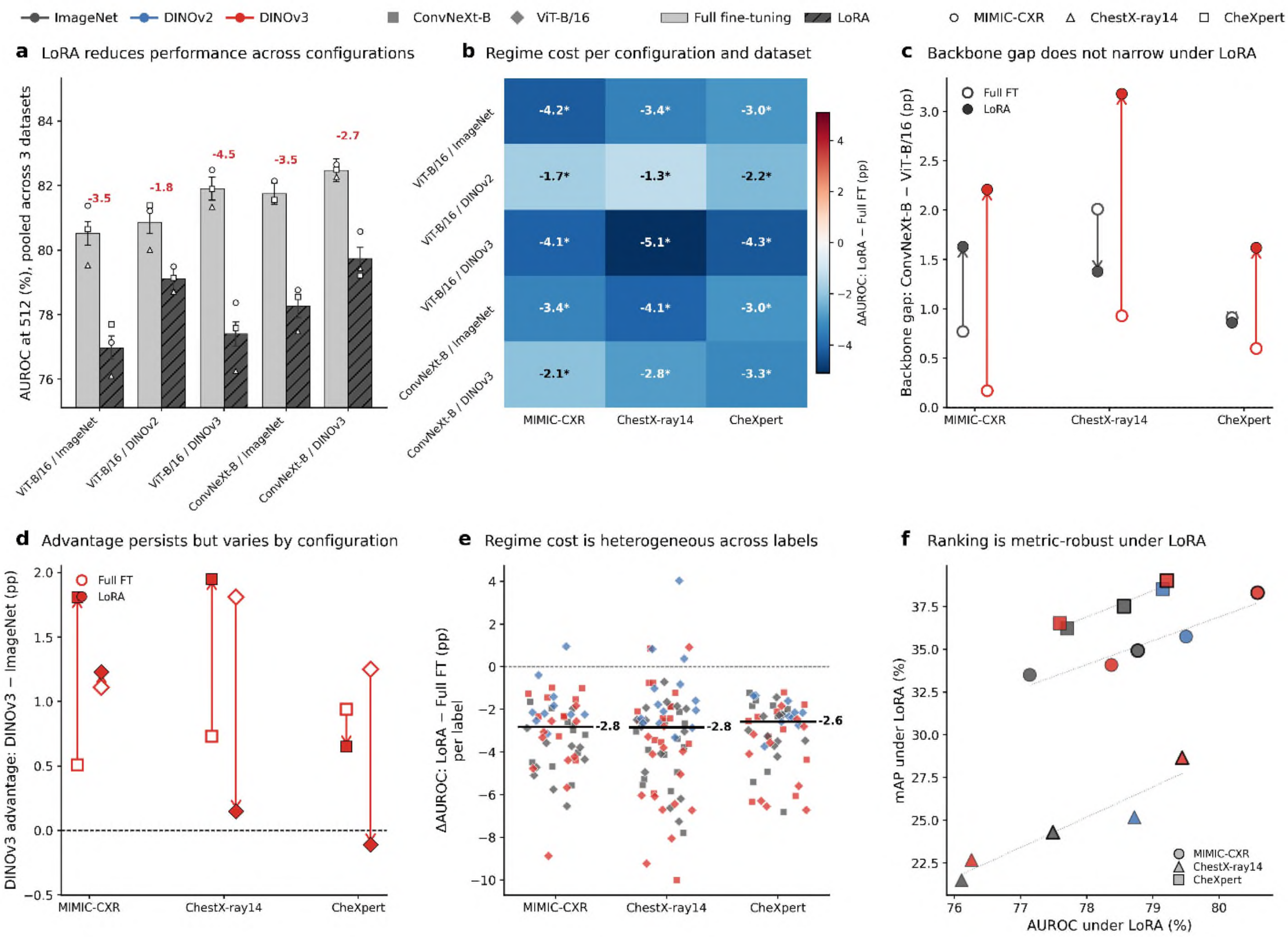

Figure 4: Parameter-efficient fine-tuning (LoRA) does not narrow the ConvNeXt-B over ViT-B/16 gap; the conclusion is robust to class-imbalance-aware metrics. All panels use the three adult datasets where LoRA was evaluated: MIMIC-CXR (training n=153,255; validation n=18,139; test n=43,793), ChestX-ray14 (training n=77,870; validation n=8,654; test n=25,596), CheXpert (training n=115,449; validation n=13,098; test n=29,318) at 512 pixels. **a** Mean AUROC across the three datasets for each of the five backbone-initialization configurations, under full fine-tuning (light bars) and LoRA (dark hatched bars). Individual dataset values are overlaid as markers (circles, triangles, squares for MIMIC-CXR, ChestX-ray14, CheXpert). Error bars denote averaged 95% bootstrap confidence intervals. Red annotations show the AUROC change from full fine-tuning to LoRA. **b** Heatmap of the per-configuration, per-dataset AUROC change when switching from full fine-tuning to LoRA. Asterisks denote non-overlapping 95% confidence intervals between regimes. **c** Backbone gap (ConvNeXt-B minus ViT-B/16 AUROC) under full fine-tuning (open circles) and LoRA (filled circles), for each dataset and initialization (excluding DINOv2, which is not evaluated on ConvNeXt-B). Arrows show the direction of change. The gap widens or persists in five of six pairs under LoRA. **d** DINOv3 advantage (DINOv3 minus ImageNet AUROC) under each regime, for each dataset and backbone. The advantage is preserved or grows in four of six pairs under LoRA and collapses for ViT-B/16 on ChestX-ray14 and CheXpert. **e** Distribution of per-label AUROC changes (LoRA minus full fine-tuning) across all available configurations, per dataset. Each point represents one pathology label under one configuration; colours denote initialization and shapes denote backbone. Horizontal bars mark the median. Regime cost is concentrated between zero and −4 pp but with substantial label-level heterogeneity. **f** Mean average precision (mAP) against AUROC under LoRA for every configuration-dataset combination. Marker shapes denote datasets (circles for MIMIC-CXR, triangles for ChestX-ray14, squares for CheXpert). Spearman rank correlations between AUROC and mAP are $\rho = 1.00$ for MIMIC-CXR ($p < 0.0001$), $\rho = 1.00$ for ChestX-ray14 ($p < 0.0001$), and $\rho = 0.90$ for CheXpert ($p = 0.037$).

Comment 2: *"As shown in lines 168–182, the reported benefits of DINOv3 initialization and higher input resolution are not uniform across datasets. Although the overall trend holds on average, several adult datasets exhibit weaker or non-significant improvements, and the pediatric cohort shows no measurable benefit. While the authors note the distinct behavior of the pediatric dataset, a more in-depth analysis that explain the observed inconsistencies between adult and pediatric populations, as well as among different adult datasets, can help the readers."*

Authors' Response and Action: We thank the Reviewer for this important point. We agree that the prior version did not explain the heterogeneity of the resolution and initialization effects in sufficient depth. In the revised manuscript, we addressed this in two ways.

First, we now treat the pediatric cohort as a dedicated boundary-condition analysis rather than a brief exception within the main benchmark. This includes a separate Results subsection and an expanded **Discussion** paragraph that consider several possible explanations, including cohort size, label-space breadth, and the distinct pediatric disease spectrum.

Second, we now analyze heterogeneity within the adult cohorts more explicitly. In the revised **Results**, we added a cross-dataset per-label and per-finding-group summary showing that the largest gains from 224 × 224 to 512 × 512 were observed for small focal lesions and boundary-dependent findings, whereas large-structure findings changed little. This helps explain why some adult datasets show stronger gains than others: the magnitude of the resolution benefit depends not only on dataset identity but also on the types of findings emphasized by the dataset and label set. We also now discuss more cautiously that differences in cohort composition and label provenance likely contribute to residual variation across adult datasets.

The new **Results** subsection reads as follows:

"The pediatric cohort shows a distinct transfer pattern that does not mirror adult resolution scaling

Pedi-CXR was not simply the weakest-performing dataset, but the only cohort that failed to follow the adult resolution-scaling pattern (**Table 2**). Whereas adult configurations showed a consistent positive lift when moving from 224 × 224 to 512 × 512 pixels, pediatric configurations clustered around zero, with a median gain of only +0.74 percentage points compared with +1.05 percentage points across adult cohorts (**Figure 3a**). This difference was also evident when plotting performance at 224 × 224 vs. 512 × 512 pixels directly: adult configurations lay predominantly above the identity line, whereas the pediatric points remained close to it (**Figure 3b**).

At the dataset level, performance differences between initialization and backbone choices were minimal in Pedi-CXR. At 512 × 512 pixels, all five evaluated configurations fell within a narrow AUROC range of 0.49 percentage points, far smaller

than in any adult cohort (**Figure 3c**). Their uncertainty intervals also overlapped broadly (**Figure 3d**), and none of the pairwise comparisons between DINOv3 and ImageNet or DINOv2 reached significance at either 224 × 224 or 512 × 512 pixels (all $p \geq 0.31$; **Table 2, Supplementary Table 1**). Even the nominally highest pediatric configuration, ViT-B/16 with DINOv2 at 512 × 512, reached only 74.1 ± 1.2, while ConvNeXt-B with DINOv3 reached 73.6 ± 1.2.
This absence of a pediatric resolution effect also held at the label level. None of the three Pedi-CXR labels showed a consistent upward shift from 224 to 512 pixels across configurations (**Figure 3e**). Moreover, when comparing DINOv3 with ImageNet at 512 × 512 using ConvNeXt-B, Pedi-CXR was the only cohort with a negative median label-level effect, whereas all adult datasets showed positive medians (**Figure 3f**). Thus, the adult result that DINOv3 benefits from higher resolution did not transfer to the pediatric setting. These findings indicate that the adult DINOv3-at-512 pattern should not be generalized automatically to pediatric chest radiography. In this cohort, increasing resolution and changing initialization or backbone had little measurable impact, suggesting that the determinants of transfer performance differ materially between pediatric and adult chest X-ray analysis."

Moreover, the **Discussion** now states:
"**Discussion**
[…]
The pediatric cohort provides an important boundary condition. Pedi-CXR was not merely lower performing overall, but qualitatively different. It showed minimal separation between initializations and almost no consistent improvement from 224 × 224 to 512 × 512 pixels. Several factors may contribute. The cohort is substantially smaller than the adult datasets, contains only three labels, and focuses on a pediatric disease spectrum that differs from the adult multi-label setting. Moreover, the labels in Pedi-CXR do not emphasize the same small focal and boundary-dependent abnormalities that benefited most from higher resolution in adults. These findings suggest that the adult DINOv3-at-512 pattern should not be generalized automatically to pediatric chest radiography. More broadly, they indicate that transfer behavior in pediatric imaging may be shaped less by high-resolution representation quality alone and more by cohort size, label ontology, and disease phenotype.
[…]"

And for the adult heterogeneity, the **Discussion** now also states:
"Resolution dependence is the central result. At 224 × 224 pixels, DINOv3 generally improved over ImageNet, yet it did not consistently exceed DINOv2. At 512 × 512 pixels, however, DINOv3 became the strongest initialization across most adult cohorts, with the clearest gains seen when paired with ConvNeXt-B. This pattern is consistent with the design of DINOv3, which emphasizes fine-grained feature preservation through Gram-anchored distillation and explicit high-resolution adaptation. It also

aligns with the fact that chest radiographs contain clinically relevant structures at much finer spatial scales than are typically preserved in natural-image benchmarks. At the same time, the magnitude of the gain was not uniform across adult datasets, which likely reflects differences in finding composition, label provenance, and cohort characteristics rather than a single dataset-level factor alone. Our cross-dataset finding-group summary supports this interpretation: the largest average gains from 224 × 224 to 512 × 512 were seen for small focal lesions and boundary-dependent findings, whereas large-structure abnormalities such as cardiomegaly and pleural effusion changed little. In practical terms, DINOv3 appears to matter most when diagnostic information is spatially subtle."

Comment 3: *"The finding that frozen features from DINOv3 underperform fully fine-tuned models is not unexpected, given that DINOv3 is pretrained exclusively on natural images and not adapted to the medical domain. A more informative comparison would be to evaluate frozen features from medical-specific self-supervised models, such as MedDINOv3 [1], to determine whether the observed performance gap reflects a general limitation of frozen representations or is primarily due to domain mismatch."*

Authors' Response and Action: We thank the Reviewer for this important and well-taken point. We agree that a comparison against frozen medical-domain self-supervised models, such as MedDINOv3, would be highly informative for disentangling domain mismatch from the broader question of whether frozen representations can approach the performance of task-adapted models in chest radiograph classification.

In the revised manuscript, we substantially restructured the study and added several new follow-up experiments to address the main interpretive questions raised by the benchmark, including parameter-efficient adaptation, synthetic label corruption, external validation across institutions, targeted 1024 × 1024 scaling with explicit computational analysis, and a dedicated pediatric boundary-condition analysis. Within this revised design, the frozen DINOv3-7B experiment was intended as a focused practical comparison between a very large natural-image-pretrained frozen encoder and smaller task-adapted mid-sized backbones, rather than as an exhaustive benchmark of frozen-representation strategies.

We therefore agree that the conclusion should be interpreted narrowly. In the revised manuscript, we now clarify that the frozen-feature result applies specifically to the natural-image-pretrained DINOv3-7B encoder evaluated here, and does not by itself establish that frozen representations are generally inferior to fine-tuned models irrespective of pretraining domain. We also now acknowledge explicitly in the **Discussion** and **Limitations** that domain mismatch is a plausible contributor to the observed gap, and that future work should compare frozen natural-image and medical-domain self-supervised encoders under matched evaluation settings.

The corresponding **Results** subsection reads as follows:

"Frozen billion-parameter features remain inferior to task-adapted mid-sized models

We finally compared frozen DINOv3-7B features coupled to a lightweight classifier with the best task-adapted ViT-B/16 and ConvNeXt-B models obtained under full fine-tuning across all seven datasets and both universal resolutions (**Figure 6e,f, Supplementary Table 6, Supplementary Figure 9).** Despite its much larger scale, the frozen encoder consistently underperformed the best fine-tuned mid-sized models. At 224 × 224, the gap to the best task-adapted model ranged from 4.6 percentage points in Pedi-CXR (68.8 ± 1.3 vs. 73.4 ± 1.2) to 7.4 percentage points in VinDr-CXR (82.8 ± 0.8 vs. 90.2 ± 0.6). Similar deficits were seen in adult cohorts such as ChestX-ray14 (76.1 ± 0.2 vs. 80.4 ± 0.2), CheXpert (76.8 ± 0.2 vs. 80.5 ± 0.2), MIMIC-CXR (77.1 ± 0.2 vs. 81.2 ± 0.1), and UKA-CXR (83.8 ± 0.1 vs. 88.2 ± 0.1).

The same pattern persisted at 512 × 512. Frozen DINOv3-7B reached 69.4 ± 1.3 in Pedi-CXR, 86.8 ± 0.6 in VinDr-CXR, 77.7 ± 0.3 in ChestX-ray14, 78.9 ± 0.2 in CheXpert, 78.9 ± 0.2 in MIMIC-CXR, and 85.4 ± 0.1 in UKA-CXR, whereas the best task-adapted models achieved 74.1 ± 1.2, 90.5 ± 0.5, 82.3 ± 0.2, 82.5 ± 0.1, 82.7 ± 0.2, and 88.5 ± 0.1, respectively. Thus, the frozen-to-fine-tuned gap remained substantial at higher resolution, typically around 3 to 5 percentage points and reaching 4.6 points in ChestX-ray14 and 3.8 points in MIMIC-CXR.

Increasing resolution from 224 × 224 to 512 × 512 did not close this gap systematically (**Figure 6f**). In some datasets the difference narrowed modestly, such as VinDr-CXR and UKA-CXR, whereas in others it remained nearly unchanged or widened slightly, including ChestX-ray14. The overall conclusion therefore did not depend on resolution: in the present setting, larger frozen natural-image representations did not substitute for downstream adaptation. These results show that model scale alone was insufficient to match the performance of task-adapted mid-sized backbones in chest radiograph classification. Even against the 7B-parameter natural-image-pretrained foundation encoder evaluated here, full adaptation of substantially smaller ViT-B/16 and ConvNeXt-B models remained decisively superior, underscoring the importance of domain- and task-specific fine-tuning in medical imaging."

The relevant **Discussion** paragraph now reads:

"The frozen 7B comparison provides a final practical lesson. Despite its scale, frozen DINOv3-7B with a lightweight classifier was consistently inferior to the best fine-tuned base models across all datasets and both universal resolutions. The gap remained substantial even at 512 × 512 pixels and did not close systematically with higher resolution. This result is not entirely surprising, because the frozen encoder was pretrained on natural images and never adapted to the clinical domain. Still, it is informative: in the present setting, model size alone did not compensate for the absence of task-specific adaptation. For chest radiograph classification, carefully

adapted 86 to 89 million-parameter models delivered more diagnostic value than the much larger frozen vision encoder evaluated here. An important next step will be to compare such frozen natural-image features directly against frozen representations from medical-domain self-supervised models, in order to disentangle the effect of domain mismatch from the broader question of whether frozen representations can approach the performance of task-adapted models in radiology."

The **Limitations** part of the **Discussion** section now also includes:
"This study has several limitations. First, although […] Fifth, the frozen-feature comparison was restricted to a natural-image-pretrained DINOv3-7B encoder and did not include frozen medical-domain self-supervised encoders, including emerging DINOv3-based medical adaptations such as MedDINOv3, so it does not by itself distinguish domain mismatch from a more general limitation of frozen representations. [...]"

Reply to Reviewer #3
COMMSMED-25-2461

Title:
Resolution-dependent self-supervised transfer in chest radiograph classification

General Comment & Comment 1: *"SUMMARY:*
In this paper, the authors present an extensive benchmark of DINOv3 for chest x-ray (CXR) classification across seven adult and pediatric datasets. Experiments primarily focus on evaluating DINOv3 compared to its previous iteration, DINOv2, and pre-trained ImageNet model across different input resolutions and vision backbones. Their results indicate the DINOv3 at 512x512 resolution, with diminishing returns after that despite increase in computational cost and that choice of vision backbone is a major factor impacting classifier performance. Additional analysis of label-wise effects (particularly labeling source) demonstrates variability in performance. In conclusion, this work shows the potential for SSL models serving as a strong backbone for CXR classification tasks.

OVERALL IMPRESSION:
My main concern with this work has limited novelty. Prior work (including the authors, as cited) has explored every experiment covered in the paper with much more detail. Extending prior findings to a single task and reporting concordant results is quite incremental, making the paper's conclusions trivial."
In prior work [1], the authors have explored a very similar experimental setup with DINOv2. Based on a quick skim through it, the only notable additions seem to be DINOv3 and ablations across different input resolutions and vision backbones. Their previous findings and conclusions are near identical, thus making it hard to justify this work's novelty."

Authors' Response and Action: We thank the Reviewer for articulating this concern clearly. We agree that, in its earlier form, the manuscript could be read mainly as an incremental extension of our prior DINOv2 benchmark to DINOv3. In the revised version, we therefore substantially restructured the paper (please refer to our general response to the Editor on top of this letter, where we have given an overview of all the major changes) so that its contribution is no longer framed primarily as a model-update benchmark, but rather as an analysis of when DINOv3 helps, where it does not, and how its gains should be interpreted in practice.

Specifically, the revised manuscript now includes several analyses that were not part of our earlier DINOv2 study and that materially broaden the scope of the work:
(i) a dedicated pediatric boundary-condition analysis rather than treating Pedi-CXR as a minor exception,

(ii) a formal 512 × 512 vs. 1024 × 1024 comparison with explicit computational cost analysis,
(iii) a parameter-efficient adaptation analysis showing that the ConvNeXt-B over ViT-B/16 ranking persists under parameter efficient fine-tuning,
(iv) a controlled synthetic label-noise experiment showing that DINOv3's gains should not be interpreted simply as greater robustness to noisy supervision,
(v) an external-validation experiment across institutions using a harmonized seven-label transfer setting, and
(vi) a frozen 7B-feature comparison framed as a practical question about scale versus adaptation. In addition, we now include a cross-dataset finding-group analysis showing that the strongest resolution-linked gains occur for small focal and boundary-dependent findings.

Accordingly, the central message of the revised manuscript is no longer simply that DINOv3 outperforms DINOv2 in the same benchmark setting. Rather, the revised paper shows that DINOv3's benefit is conditional: it emerges reproducibly in adult cohorts at 512 × 512 pixels, does not generalize automatically to the pediatric cohort, does not continue to improve meaningfully at 1024 × 1024 despite sharply higher cost, is preserved under external validation, is not explained by superior robustness to synthetic label corruption, and does not remove the need for downstream adaptation. We believe this revised framing and the added experiments make the present study substantively different from our prior DINOv2 work.

In response to this concern, we revised the Introduction, Results, and Discussion extensively so that the manuscript now emphasizes these boundary conditions, interpretation issues, and practical operating points rather than novelty based only on the introduction of a newer self-supervised model.

Comment 2: *"While the authors claim that this paper is the "first systematic evaluation of DINOv3 for chest radiograph classification", other groups have since published DINOv3 validation across numerous medical imaging tasks (including CXR classification) [2]."*

Authors' Response and Action: We thank the Reviewer for this important point. We agree that, in light of subsequent DINOv3 medical-imaging benchmarks, the claim that this work is the "first systematic evaluation" is no longer appropriate in this form, and we have removed that wording from the revised manuscript.

At the same time, we would like to clarify the distinct scope of the present study. The work cited by the Reviewer benchmarks DINOv3 broadly across multiple 2D and 3D medical imaging tasks, including classification, segmentation, and registration, and

across many modalities, with a particular emphasis on whether DINOv3 can serve as a strong off-the-shelf encoder without domain-specific fine-tuning and how its performance scales with model size and resolution. In contrast, our revised manuscript is a task-specific large-scale study of chest radiograph classification under downstream adaptation, designed to answer a different set of questions: when DINOv3 helps in chest radiography, whether this depends on input resolution, whether the pattern differs between adult and pediatric cohorts, whether backbone ranking persists under parameter-efficient adaptation, whether the gains can be explained by robustness to noisy labels, whether they hold under external institutional transfer, and whether frozen billion-parameter features can compete with task-adapted mid-sized models.

We have therefore revised the **Introduction** to avoid an unnecessary priority claim and instead position the paper more precisely as a focused chest-radiograph analysis of the practical operating conditions and interpretation of DINOv3 transfer. Specifically, in the third paragraph we now state:
"Recent studies have begun to benchmark DINOv3 across multiple medical imaging tasks and modalities, including chest radiograph classification, but important questions remain regarding its task-specific operating conditions and interpretation in chest radiography."

In addition, the opening sentence of the final **Introduction** paragraph has been revised from a generic large-scale evaluation claim to:
"Here, we present a large-scale, chest-radiograph-focused evaluation of DINOv3 for chest radiograph classification across seven datasets comprising 816,183 anteroposterior (AP) or posteroanterior (PA) radiographs from public and internal cohorts (see **Figure 1**)."

Comment 3: *"Previous work has also extensively evaluated impact of image resolution for CXR classification. Similar to comments raised previously, the findings from this paper are aligned with those reported previously [3]. Beyond the exploration of SOTA SSL technique (vs supervised learning), there are no new conclusions drawn from experiments."*

Authors' Response and Action: We agree that the general observation that image resolution can influence chest radiograph classification performance is not new in itself, and we do not claim otherwise in the revised manuscript. The contribution of the present study is more specific: within the setting of modern self-supervised transfer, increasing resolution did not merely improve performance overall, but changed which initialization was preferable, and this effect proved to be conditional rather than universal.

In particular, the revised manuscript now shows that the DINOv3 advantage is weak at 224 × 224 but becomes reproducible in adult cohorts at 512 × 512; that this pattern does not extend to the pediatric cohort; that the strongest resolution-linked gains occur for small focal lesions and boundary-dependent findings rather than large-structure abnormalities; and that scaling further to 1024 × 1024 rarely improves performance despite sharply higher computational cost. We believe these are more specific conclusions than the general statement that "higher resolution may help," and they are central to the revised framing of the paper.

To make this clearer, we revised the Results and Discussion accordingly. The revised **Discussion** now states:
"Resolution dependence is the central result. At 224 × 224 pixels, DINOv3 generally improved over ImageNet, yet it did not consistently exceed DINOv2. At 512 × 512 pixels, however, DINOv3 became the strongest initialization across most adult cohorts, with the clearest gains seen when paired with ConvNeXt-B. This pattern is consistent with the design of DINOv3, which emphasizes fine-grained feature preservation through Gram-anchored distillation and explicit high-resolution adaptation. It also aligns with the fact that chest radiographs contain clinically relevant structures at much finer spatial scales than are typically preserved in natural-image benchmarks. At the same time, the magnitude of the gain was not uniform across adult datasets, which likely reflects differences in finding composition, label provenance, and cohort characteristics rather than a single dataset-level factor alone. Our cross-dataset finding-group summary supports this interpretation: the largest average gains from 224 × 224 to 512 × 512 were seen for small focal lesions and boundary-dependent findings, whereas large-structure abnormalities such as cardiomegaly and pleural effusion changed little. In practical terms, DINOv3 appears to matter most when diagnostic information is spatially subtle."

Comment 4: *"Expanding upon the prior point, it is quite trivial that DINOv3 would yield superior performance than DINOv2 as higher resolution. The inclusion of a high-resolution post-training phase and passing high-resolution images to the Gram teacher (prior to downsampling) both can be attributed to this improvement and are also extensively discussed in the DINOv3 paper [4]."*

Authors' Response and Action: We thank the Reviewer for this point. We agree that, from the perspective of the DINOv3 pretraining design, it is reasonable to expect that DINOv3 should benefit more from higher-resolution transfer than DINOv2. We do not intend to present that architectural rationale itself as a novel conceptual discovery. In the revised manuscript, we therefore frame this result more carefully: the value of our study is not to claim surprise that DINOv3 can benefit at higher resolution, but to show

how this expectation actually plays out in chest radiograph classification, and under which conditions it does or does not hold.
Importantly, the empirical pattern in our benchmark was not simply “higher resolution helps DINOv3 everywhere.” Rather, the revised manuscript shows that the advantage is conditional: it becomes reproducible in adult cohorts at 512 × 512, does not transfer automatically to the pediatric cohort, does not continue meaningfully at 1024 × 1024 despite sharply higher cost, and is concentrated in small focal and boundary-dependent findings rather than large-structure abnormalities. We believe these task-specific boundary conditions are not trivial consequences of the DINOv3 paper itself and are part of the practical contribution of the present work.

To reflect this more clearly, we now state in the **Discussion**:
“Resolution dependence is the central result. At 224 × 224 pixels, DINOv3 generally improved over ImageNet, yet it did not consistently exceed DINOv2. At 512 × 512 pixels, however, DINOv3 became the strongest initialization across most adult cohorts, with the clearest gains seen when paired with ConvNeXt-B. This pattern is consistent with the design of DINOv3, which emphasizes fine-grained feature preservation through Gram-anchored distillation and explicit high-resolution adaptation. It also aligns with the fact that chest radiographs contain clinically relevant structures at much finer spatial scales than are typically preserved in natural-image benchmarks. At the same time, the magnitude of the gain was not uniform across adult datasets, which likely reflects differences in finding composition, label provenance, and cohort characteristics rather than a single dataset-level factor alone. Our cross-dataset finding-group summary supports this interpretation: the largest average gains from 224 × 224 to 512 × 512 were seen for small focal lesions and boundary-dependent findings, whereas large-structure abnormalities such as cardiomegaly and pleural effusion changed little. In practical terms, DINOv3 appears to matter most when diagnostic information is spatially subtle.”

Comment 5: *“Performance improvements in ConvNeXt could be attributed to the data-efficiency and inductive biases of CNNs compared to ViTs. Given the small dataset sizes, it is trivial that ViT would underperform ConvNeXt. For context, the original DINO paper showed superior performance with ViTs [5] and ConvNeXt paper showed comparable performance of Swin Transformers [6]. Without more in-depth analysis, claiming that CNNs are outright better than Transformers for CXR classification may be an overstatement.”*

Authors’ Response and Action: We thank the Reviewer for this important point. We agree that a blanket claim that CNNs are universally better than transformers for chest radiograph classification would be too strong. In the revised manuscript, we therefore frame this result more carefully. Our conclusion is not that convolutional backbones are

inherently superior in all radiology settings, but that in the present benchmark, at base model scale and under the evaluated transfer regimes, ConvNeXt-B provided the more reliable transfer behavior than ViT-B/16.

To address this concern directly, we added a dedicated parameter-efficient adaptation analysis in the revised manuscript, implemented with LoRA on ChestX-ray14, CheXpert, and MIMIC-CXR at 512 × 512 pixels. This analysis was specifically designed to test whether the ConvNeXt-B over ViT-B/16 ranking could be explained mainly by the use of full end-to-end fine-tuning. The new results showed that LoRA did not rescue ViT-B/16 or narrow the backbone gap. Instead, ConvNeXt-B remained superior in five of six backbone-initialization pairs, and in several cases the gap widened under LoRA. Thus, in our benchmark, the backbone ranking was not specific to full fine-tuning alone.

The revised **Results** subsection now reads:

"ConvNeXt-B remains superior to ViT-B/16 under both full and parameter-efficient adaptation

Under full fine-tuning, ConvNeXt-B consistently outperformed ViT-B/16 across adult datasets and resolutions (**Table 2**), with the gap most apparent at 512 × 512 pixels. For example, with DINOv3 initialization at 512 × 512, ConvNeXt-B reached 82.7 ± 0.2 vs. 82.5 ± 0.1 in MIMIC-CXR, 82.3 ± 0.2 vs. 81.3 ± 0.2 in ChestX-ray14, and 82.5 ± 0.1 vs. 81.9 ± 0.2 in CheXpert. Thus, the main benchmark already indicated a stable backbone advantage under standard end-to-end optimization. We then asked whether this result could be explained simply by the use of full fine-tuning by replacing it with a parameter-efficient adaptation regime, implemented here with LoRA (**Figure 4a-c, Supplementary Table 4**). Across all evaluated dataset-configuration pairs, LoRA reduced AUROC relative to full fine-tuning. The magnitude of this loss ranged from 1.3 ± 0.3 to 5.1 ± 0.3 percentage points for ViT-B/16 and from 2.1 ± 0.2 to 4.1 ± 0.3 percentage points for ConvNeXt-B. For DINOv3 specifically, the drop under LoRA was larger for ViT-B/16 than for ConvNeXt-B in all three datasets: 5.1 ± 0.3 vs. 2.8 ± 0.3 in ChestX-ray14, 4.3 ± 0.2 vs. 3.3 ± 0.2 in CheXpert, and 4.1 ± 0.2 vs. 2.1 ± 0.2 in MIMIC-CXR. Thus, reduced adaptation capacity did not preferentially benefit ViT-B/16.

Accordingly, the backbone gap did not narrow under parameter-efficient adaptation. Instead, ConvNeXt-B remained superior to ViT-B/16 in five of six backbone-initialization pairs, and the gap widened in several cases (**Figure 4c**). For DINOv3, the ConvNeXt-B minus ViT-B/16 difference increased from 1.0 to 3.2 percentage points in ChestX-ray14 and from 0.2 to 2.2 percentage points in MIMIC-CXR, while remaining positive in CheXpert (0.6 to 1.6 percentage points). The same qualitative pattern held for ImageNet initialization. These findings indicate that the ConvNeXt advantage cannot be attributed merely to having used full end-to-end fine-tuning in the main benchmark.

The initialization story also remained broadly stable. Under parameter-efficient adaptation, DINOv3 retained an advantage over ImageNet for ConvNeXt-B in all three datasets and for ViT-B/16 in MIMIC-CXR, but this advantage weakened or disappeared

for ViT-B/16 in ChestX-ray14 and CheXpert (**Figure 4d**). This suggests that ViT-B/16 was more sensitive to the restricted adaptation regime, whereas ConvNeXt-B preserved both its stronger absolute performance and its relative benefit from DINOv3 initialization.
At the label level, parameter-efficient adaptation produced heterogeneous but predominantly negative changes, with median per-label AUROC shifts of about -2.8 percentage points in MIMIC-CXR, -2.8 percentage points in ChestX-ray14, and -2.6 percentage points in CheXpert (**Figure 4e**). Importantly, these shifts did not alter the overall ranking of configurations. Class-imbalance-aware evaluation supported the same conclusion: under LoRA, AUROC and mAP remained strongly aligned across configurations (Spearman $\rho = 1.00$ for MIMIC-CXR, $\rho = 1.00$ for ChestX-ray14, and $\rho = 0.90$ for CheXpert; all $p \leq 0.037$), indicating that the backbone comparison was not an artifact of AUROC alone (**Figure 4f**). The superiority of ConvNeXt-B over ViT-B/16 cannot be explained simply by the use of full fine-tuning. Instead, it persisted under both full and parameter-efficient adaptation, supporting a genuine backbone-level difference in transfer behavior for chest radiograph classification."

We also revised the **Discussion** to avoid overstatement. The relevant paragraph now reads:
"The backbone findings were also robust to changes in adaptation regime. Under full fine-tuning, ConvNeXt-B consistently outperformed ViT-B/16, particularly at 512 × 512 pixels. Parameter-efficient adaptation, implemented here with LoRA, did not narrow this gap. Instead, it reduced performance in all evaluated settings and generally affected ViT-B/16 more strongly than ConvNeXt-B, especially for DINOv3 initialization. This suggests that the ConvNeXt advantage cannot be explained simply by the use of full end-to-end fine-tuning. A plausible interpretation is that convolutional inductive biases and hierarchical local feature aggregation remain advantageous in chest radiograph analysis, particularly when diagnostically relevant information is spatially local and when high-resolution inputs emphasize subtle boundaries and fine structures. This interpretation is also consistent with our scaling results, in which ConvNeXt-B incurred a smaller computational penalty than ViT-B/16 at higher resolution and was the only backbone to show significant gains at 1024 × 1024 in MIMIC-CXR. While this does not prove that convolutional backbones are universally superior to transformers in radiology, it does show that, at base scale and under both full and parameter-efficient adaptation, ConvNeXt-B provided the more reliable transfer behavior in our benchmark."

Finally, the **Limitations** part of **Discussion** section now states:
"Third, the backbone comparison under parameter-efficient adaptation was restricted to LoRA and to three adult datasets, so broader conclusions about all parameter-efficient fine-tuning strategies or all model scales would be premature."

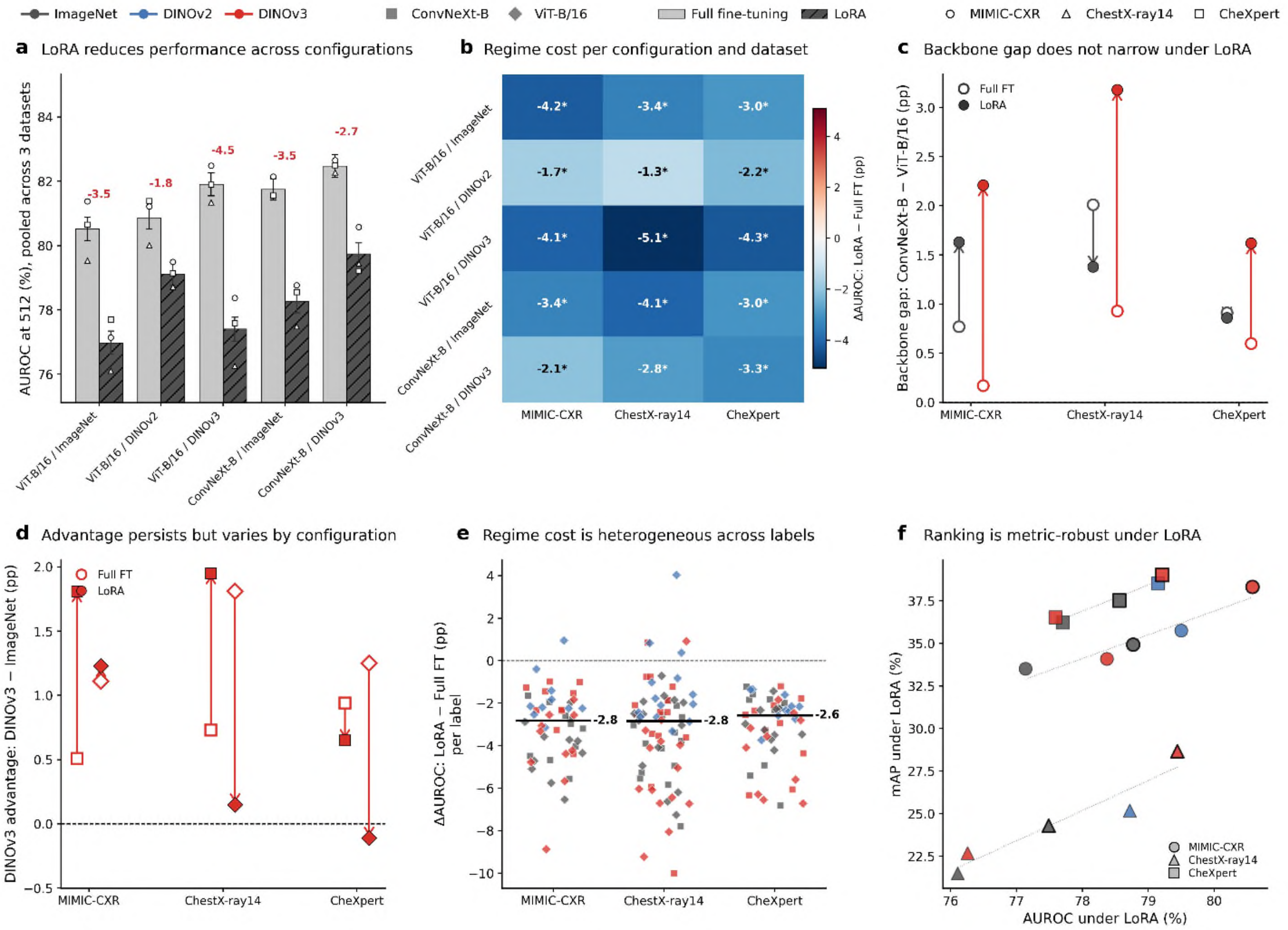

Figure 4: Parameter-efficient fine-tuning (LoRA) does not narrow the ConvNeXt-B over ViT-B/16 gap; the conclusion is robust to class-imbalance-aware metrics.

Comment 6: *"The finding that frozen ImageNet pre-trained DINOv3 with linear probe significantly underperforms fully fine-tuned equivalents is similarly trivial and covered extensively in prior literature [7]."*

Authors' Response and Action: We thank the Reviewer for this point. We agree that, at a general conceptual level, the observation that fully fine-tuned models can outperform linear probing is well established in the transfer-learning literature. We do not intend to present that broad principle itself as a novel finding. In the revised manuscript, we therefore frame this analysis more narrowly and more practically.

Specifically, the purpose of this experiment in the revised paper is not to argue that fine-tuning is generally better than linear probing in the abstract, but to test a concrete question relevant to current foundation-model use in radiology: whether a very large frozen DINOv3-7B encoder can offset the need for downstream adaptation when compared against much smaller task-adapted backbones. In our benchmark, it did not. Across all seven datasets and both universal resolutions, the frozen 7B features

consistently underperformed the best fully adapted ViT-B/16 and ConvNeXt-B models, despite the much larger encoder scale. We believe this task-specific result remains practically informative, especially because it addresses a common assumption that sufficiently large frozen encoders may be adequate for downstream medical imaging tasks.

To reflect this more clearly, we revised the **Results** and **Discussion** so that this experiment is presented as a practical comparison of scale versus adaptation, rather than as a broad conceptual claim. The relevant **Discussion** paragraph now reads:
"The frozen 7B comparison provides a final practical lesson. Despite its scale, frozen DINOv3-7B with a lightweight classifier was consistently inferior to the best fine-tuned base models across all datasets and both universal resolutions. The gap remained substantial even at 512 × 512 pixels and did not close systematically with higher resolution. This result is not entirely surprising, because the frozen encoder was pretrained on natural images and never adapted to the clinical domain. Still, it is informative: in the present setting, model size alone did not compensate for the absence of task-specific adaptation. For chest radiograph classification, carefully adapted 86 to 89 million-parameter models delivered more diagnostic value than the much larger frozen vision encoder evaluated here. An important next step will be to compare such frozen natural-image features directly against frozen representations from medical-domain self-supervised models, in order to disentangle the effect of domain mismatch from the broader question of whether frozen representations can approach the performance of task-adapted models in radiology."

We also now state the corresponding **limitation** more explicitly in the manuscript:
"This study has several limitations. First, […] Fifth, the frozen-feature comparison was restricted to a natural-image-pretrained DINOv3-7B encoder and did not include frozen medical-domain self-supervised encoders, including emerging DINOv3-based medical adaptations such as MedDINOv3, so it does not by itself distinguish domain mismatch from a more general limitation of frozen representations. […]"

Comment 7: *"Could the authors comment on why performance is much lower on pediatric datasets? Is it simply lack of large, high-quality data to yield comparable performance?"*

Authors' Response and Action: We thank the Reviewer for this important point. We agree that the pediatric result required substantially deeper analysis and discussion. In the revised manuscript, we therefore elevated Pedi-CXR from a brief exception within the benchmark to a dedicated **Results** subsection and expanded the **Discussion** accordingly. The revised text now makes clear that Pedi-CXR was not simply the

lowest-performing cohort, but the only cohort that failed to follow the adult resolution-scaling pattern.

Our interpretation is that reduced performance in the pediatric cohort is unlikely to be explained by a single factor alone. Smaller cohort size is certainly a plausible contributor, but the revised manuscript now discusses additional reasons as well: the much narrower label ontology, the distinct pediatric disease spectrum, and the fact that the available pediatric labels do not emphasize the same small focal and boundary-dependent abnormalities that benefited most from higher resolution in the adult cohorts. Thus, the revised paper treats the pediatric result as a meaningful boundary condition rather than simply as a data-limited outlier.

The new **Results** subsection reads as follows:

"The pediatric cohort shows a distinct transfer pattern that does not mirror adult resolution scaling

Pedi-CXR was not simply the weakest-performing dataset, but the only cohort that failed to follow the adult resolution-scaling pattern (**Table 2**). Whereas adult configurations showed a consistent positive lift when moving from 224 × 224 to 512 × 512 pixels, pediatric configurations clustered around zero, with a median gain of only +0.74 percentage points compared with +1.05 percentage points across adult cohorts (**Figure 3a**). This difference was also evident when plotting performance at 224 × 224 vs. 512 × 512 pixels directly: adult configurations lay predominantly above the identity line, whereas the pediatric points remained close to it (**Figure 3b**).

At the dataset level, performance differences between initialization and backbone choices were minimal in Pedi-CXR. At 512 × 512 pixels, all five evaluated configurations fell within a narrow AUROC range of 0.49 percentage points, far smaller than in any adult cohort (**Figure 3c**). Their uncertainty intervals also overlapped broadly (**Figure 3d**), and none of the pairwise comparisons between DINOv3 and ImageNet or DINOv2 reached significance at either 224 × 224 or 512 × 512 pixels (all $p \geq 0.31$; **Table 2, Supplementary Table 1**). Even the nominally highest pediatric configuration, ViT-B/16 with DINOv2 at 512 × 512, reached only 74.1 ± 1.2, while ConvNeXt-B with DINOv3 reached 73.6 ± 1.2.

This absence of a pediatric resolution effect also held at the label level. None of the three Pedi-CXR labels showed a consistent upward shift from 224 to 512 pixels across configurations (**Figure 3e**). Moreover, when comparing DINOv3 with ImageNet at 512 × 512 using ConvNeXt-B, Pedi-CXR was the only cohort with a negative median label-level effect, whereas all adult datasets showed positive medians (**Figure 3f**). Thus, the adult result that DINOv3 benefits from higher resolution did not transfer to the pediatric setting. These findings indicate that the adult DINOv3-at-512 pattern should not be generalized automatically to pediatric chest radiography. In this cohort, increasing resolution and changing initialization or backbone had little measurable impact,

suggesting that the determinants of transfer performance differ materially between pediatric and adult chest X-ray analysis."

Moreover, the **Discussion** now states:

"**Discussion**

[…]

The pediatric cohort provides an important boundary condition. Pedi-CXR was not merely lower performing overall, but qualitatively different. It showed minimal separation between initializations and almost no consistent improvement from 224 × 224 to 512 × 512 pixels. Several factors may contribute. The cohort is substantially smaller than the adult datasets, contains only three labels, and focuses on a pediatric disease spectrum that differs from the adult multi-label setting. Moreover, the labels in Pedi-CXR do not emphasize the same small focal and boundary-dependent abnormalities that benefited most from higher resolution in adults. These findings suggest that the adult DINOv3-at-512 pattern should not be generalized automatically to pediatric chest radiography. More broadly, they indicate that transfer behavior in pediatric imaging may be shaped less by high-resolution representation quality alone and more by cohort size, label ontology, and disease phenotype.

[…]"

Reply to Reviewer #4
COMMSMED-25-2461

Title:
Resolution-dependent self-supervised transfer in chest radiograph classification

Major Comment 1: *"While the paper evaluates DINOv3 in the context of medical imaging, it does not involve any retraining or adaptation of DINOv3 on medical images. The study simply applies pretrained weights from natural image training to downstream classification tasks, which is essentially a transfer learning evaluation. To strengthen the contribution, a broader and more systematic exploration is warranted."*

> **Authors' Response and Action**: We thank the Reviewer for this important point. We agree that the present study is fundamentally a transfer-learning evaluation rather than a study of medical-domain pretraining or retraining of DINOv3 itself. This is indeed the intended scope of the work. Our goal was to assess how far publicly available natural-image-pretrained DINOv3 checkpoints transfer to chest radiograph classification under clinically relevant downstream conditions, rather than to develop a new medically pretrained variant of DINOv3.
>
> At the same time, in response to this concern, we substantially broadened and restructured the revised manuscript so that it is no longer limited to a narrow downstream benchmark. Specifically, the revised version now includes a more systematic exploration of the practical operating conditions and interpretation of DINOv3 transfer, including:
> (i) a dedicated pediatric boundary-condition analysis,
> (ii) formal 512 × 512 vs. 1024 × 1024 comparisons with explicit computational cost analysis,
> (iii) a parameter-efficient adaptation analysis using LoRA,
> (iv) a controlled synthetic label-noise experiment,
> (v) external validation across institutions using a harmonized seven-label setting, and
> (vi) a frozen 7B-feature comparison framed as a practical question of scale versus downstream adaptation. In addition, we now include a cross-dataset finding-group analysis showing that the largest resolution-linked gains occur for small focal and boundary-dependent findings.
> We therefore agree with the Reviewer that the manuscript should be understood as a transfer-learning study, and we have revised the framing of the **Introduction, Results,** and **Discussion** accordingly. The contribution of the revised paper is not retraining DINOv3 on medical images, but rather establishing when natural-image-pretrained DINOv3 transfer is useful in chest radiography, where it fails, and how those gains should be interpreted in practice.

Major Comment 2: *“Only one variant of each of the two backbone architectures is evaluated under full finetuning. Given that ViT has six variants and ConvNeXt has four, evaluating only the Base variant is insufficient to support generalizable conclusions about backbone behavior. The same applies to DINOv2. While a frozen ViT-7B is included as an additional comparison, this represents a fundamentally different experimental condition (frozen features with a lightweight head) rather than a systematic scaling analysis, and therefore does not substitute for evaluating multiple finetuned variants across model sizes.”*

Authors’ Response and Action: We thank the Reviewer for this important point. We agree that the present study does not provide a systematic scaling analysis across all ViT, ConvNeXt, or DINOv2 model sizes, and that conclusions about backbone behavior should therefore not be interpreted as applying to all variants of these families. This was not the intended scope of the work. Instead, we chose one representative base-scale model per backbone family, ViT-B/16 and ConvNeXt-B, in order to isolate the effects of initialization, input resolution, and downstream adaptation regime across a large multi-dataset benchmark while keeping the overall design tractable.
We also agree that the frozen ViT-7B comparison does not substitute for a true multi-scale finetuning study. In the revised manuscript, we therefore frame that experiment more narrowly as a practical scale-versus-adaptation comparison, rather than as evidence about architectural scaling within the ViT family.

To address this concern, we revised the manuscript so that the backbone conclusions are stated more carefully. The **Discussion** now explicitly limits the claim to the base scale and the evaluated transfer regimes, rather than making a broader statement about CNNs versus transformers in general.
The relevant **Discussion** text now reads:
“While this does not prove that convolutional backbones are universally superior to transformers in radiology, it does show that, at base scale and under both full and parameter-efficient adaptation, ConvNeXt-B provided the more reliable transfer behavior in our benchmark.”

In addition, the **Limitations** part of **Discussion** section now states:
“Third, the backbone comparison under parameter-efficient adaptation was restricted to LoRA and to three adult datasets, so broader conclusions about all parameter-efficient fine-tuning strategies or all model scales would be premature.”

Major Comment 3: *"It is unclear whether any hyperparameter tuning was performed. In 2D image classification, performance can vary substantially depending on the choice of learning rate, number of training epochs, and other training settings. While batch size may reasonably be constrained by model size, a minimum search over learning rate and epoch count should have been conducted. Given that methodological novelty is limited, comprehensive tuning is necessary for the findings to be considered reliable."*

Authors' Response and Action: We thank the Reviewer for this important point. We agree that the previous version did not describe the optimization setup with sufficient clarity. Our study was designed as a controlled transfer benchmark under a shared training protocol, rather than as an exhaustive hyperparameter-optimization study for each dataset-model pair. We therefore did not perform a formal large-scale hyperparameter search across all configurations.

However, the learning rate used for full fine-tuning was not chosen arbitrarily. As now clarified in the revised **Methods**, the value of 10^{-5} was selected based on empirical testing of multiple candidate values in the present study, where it provided the most stable convergence across datasets and model configurations. Although we did not perform a formal hyperparameter search, this value was also consistent with the range used successfully in our prior work on chest radiograph classification across several related settings and applications [1–6]. In addition, we now report the exact hardware and batch sizes used for the main experiments and for the LoRA experiments, which improves reproducibility substantially. Thus, we agree with the Reviewer that more extensive hyperparameter optimization could in principle further strengthen the study, but our goal here was to compare initialization strategies, backbones, and resolutions under a fixed and transparent optimization protocol, rather than to maximize the absolute performance of each individual configuration through separate tuning.

The updated **Methods** segment now reads as follows:

"Main full fine-tuning benchmark

Universal experiments were performed at 224 × 224 and 512 × 512 pixels across all seven datasets. To probe whether the 512 × 512 advantage extended further, we additionally evaluated 1024 × 1024 pixels on three representative cohorts spanning pediatric and adult settings, namely Pedi-CXR, ChestX-ray14, and MIMIC-CXR.

All full fine-tuning experiments were optimized with AdamW using a learning rate of 10^{-5} and no weight decay. This learning rate was chosen based on empirical testing of multiple candidate values in the present study, where it provided the most stable convergence across datasets and model configurations, and it is also consistent with the range used in our prior work on chest radiograph classification. Across all experiments, data augmentation consisted of random horizontal flips and random rotations up to 7°. The loss function was binary weighted cross-entropy, with class weights set inversely proportional to the frequency of each label in the training set. All training was performed in full 32-bit floating point precision on a single NVIDIA L40S

GPU with 48 GB VRAM and Intel Xeon Silver 4310 CPUs. For full fine-tuning, batch size was 128 at 224 × 224, 64 at 512 × 512, and 16 at 1024 × 1024. To preserve comparability across initialization strategies, backbones, and resolutions, all main benchmark models were trained under this shared optimization protocol rather than under separate per-configuration hyperparameter searches. […]"

Major Comment 4: *"Although multiple datasets are used, each dataset appears to be split only into training and test sets without a dedicated validation set. An explicit validation split should be used for hyperparameter selection and model development, separate from the held-out test set. Without this, it is difficult to rule out overfitting to the test set and the evaluation may reflect internal fit rather than true generalization."*

Authors' Response and Action: We thank the Reviewer for this important comment. We would like to clarify that, in the actual experiments, we did use dedicated validation data for model development in all datasets where patient-wise subdivision was possible. Specifically, for ChestX-ray14, PadChest, CheXpert, MIMIC-CXR, and UKA-CXR, we first created patient-wise train-test splits and then reserved 10% of the training portion as a validation set, which was used for model development and monitoring. The held-out test sets were used only for the final reported evaluation. Thus, for these datasets, the reported results are based on a genuine train-validation-test setup rather than a training-test-only design.

The Reviewer is correct that this was not described clearly enough in the previous version. In our earlier reporting style, consistent with several of our prior works, we mainly emphasized the held-out test sets in the manuscript and referred to the remainder broadly as training data, assuming that the internal subdivision of the training portion for validation was standard practice and could in principle be adapted by other researchers depending on their preferred development protocol. In the revised manuscript, we have now clarified the split strategy throughout the paper and updated **Table 1** to report training, validation, and test counts explicitly.

Two datasets are exceptions: Pedi-CXR and VinDr-CXR. For these datasets, we used the predefined training and test partitions released by the dataset authors, but no additional validation split was created because patient identifiers are not available. Under those circumstances, randomly sampling a validation subset from the provided training split could have introduced patient-level information leakage across splits. We now state this explicitly in both the dataset descriptions and the **Table 1** caption.

The revised **Methods** now state:

"This study included a total of n = 816,183 AP or PA chest radiographs from seven international cohorts encompassing both adult and pediatric populations. Patients ranged in age from infancy to over 111 years. The datasets span diverse geographic

regions (Asia, Europe, and North America), label generation strategies (manual annotation, rule-based natural language processing (NLP), and hybrid approaches), and clinical contexts (inpatient, outpatient, intensive care, and pediatrics). A detailed overview of dataset characteristics is provided in **Table 1**. Below, we describe each dataset. For datasets with predefined train and test partitions but without patient identifiers (Pedi-CXR and VinDr-CXR), no additional validation split was created in order to avoid potential patient-level information leakage."

and, for example, for the other datasets:

"Following prior work, we first generated a patient-wise 80%/20% train-test split and then reserved 10% of the training portion for validation, resulting in n = 77,870 training, n = 8,654 validation, and n = 25,596 test images."

The revised **Experimental design** section now also states this explicitly:

"To ensure consistency across experiments, we applied a unified preprocessing workflow to all datasets and kept the train, validation, and test partitions fixed throughout the study. For ChestX-ray14, PadChest, CheXpert, MIMIC-CXR, and UKA-CXR, patient-wise train-test splits were created and 10% of the training portion was reserved as a validation set, resulting in the splits listed in Table 1. For Pedi-CXR and VinDr-CXR, we used the original training and test partitions provided by the dataset authors. No additional validation split was created for these two datasets because patient identifiers were unavailable, precluding leakage-safe subdivision of the training set."

In addition, the revised **Table 1** caption now states:

"For Pedi-CXR and VinDr-CXR, no validation split was created because patient identifiers were unavailable, precluding leakage-safe subdivision of the provided training sets."

In addition, the revised manuscript now includes a dedicated external-validation experiment, in which models trained on MIMIC-CXR were evaluated on the fully held-out external datasets ChestX-ray14 and CheXpert using a harmonized seven-label setting. This further reduces the concern that the reported findings merely reflect internal fit to dataset-specific splits and shows that the main adult benchmark pattern remained visible under cross-institutional transfer.

Major Comment 5: *"The primary metric is ROC-AUC; however, for datasets with substantial label imbalance such as ChestX-ray14 and CheXpert, this metric may not be the most appropriate. Metrics that better account for class imbalance, such as mean average precision (mAP) or PR-AUC, should be considered as primary or complementary evaluation criteria."*

Authors' Response and Action: We thank the Reviewer for this important point. We agree that, in multilabel chest radiograph classification with substantial class

imbalance, AUROC alone does not fully capture all aspects of model behavior. In the revised manuscript, we therefore strengthened the evaluation accordingly.

Specifically, while we retained mean AUROC across labels as the primary benchmark metric for the main seven-dataset comparison, in order to preserve comparability with prior chest-radiograph literature and with our earlier work, we now report PR-AUC and mAP as complementary class-imbalance-aware metrics in the targeted follow-up analyses where this issue is especially relevant, namely the parameter-efficient adaptation experiments, the synthetic label-noise experiment, and the external-validation experiments. Across these analyses, PR-AUC and mAP closely tracked AUROC and supported the same overall ranking of configurations, which reduces the likelihood that the main conclusions are an artifact of AUROC alone.

Major Comment 6: *"The Methods section contains detailed mathematical descriptions of the DINOv3 pretraining objectives, including Gram anchoring and the associated loss formulations. As these are entirely derived from the original DINOv3 paper and do not constitute a contribution of the present work, we recommend condensing this section considerably. A brief summary with appropriate citation would suffice."*

Authors' Response and Action: We thank the Reviewer for this helpful suggestion. We agree. In the revised manuscript, we substantially condensed this section and removed the detailed mathematical formulations of the DINOv3 pretraining losses. The **Methods** now provide only a brief summary of the DINOv3 pretraining framework, with appropriate citation to the original DINOv3 paper, and make clear that these objectives were not retrained on medical images in the present study, but only provide the basis for the publicly released pretrained checkpoints used as initialization.

The revised section now reads:

"Pretrained initialization strategies and backbone architectures
This study was designed as a controlled transfer-learning benchmark rather than a medical-domain pretraining study. Accordingly, DINOv2 and DINOv3 were not retrained on chest radiographs; instead, we used the publicly released pretrained checkpoints directly as initialization for downstream chest radiograph classification. Relative to DINOv2, DINOv3 extends the same general self-supervised framework by introducing Gram-anchored refinement to stabilize dense patch representations during extended training and explicit high-resolution adaptation to support transfer to larger input sizes. These properties motivated our evaluation across resolutions, architectures, and adaptation regimes."

Minor Comment 1: *"The ViT-B/16 ImageNet-21K weights are referenced via timm, while all other pretrained weights are linked to Hugging Face. For consistency and reproducibility, a corresponding Hugging Face link should also be provided for this model where available, while the timm identifier may be retained as additional reference."*

> **Authors' Response and Action**: We agree that using a consistent link format improves reproducibility and readability. In the revised manuscript, we have therefore updated the Code availability and reproducibility section accordingly by adding the corresponding Hugging Face reference for the ViT-B/16 ImageNet-21K weights, while retaining the original timm model identifier as an additional implementation reference.

References:

[1] Tayebi Arasteh, S., Ziller, A., Kuhl, C. et al. Preserving fairness and diagnostic accuracy in private large-scale AI models for medical imaging. *Communications Medicine* 4, 46 (2024). https://doi.org/10.1038/s43856-024-00462-6

[2] Tayebi Arasteh, S., Isfort, P., Saehn, M. et al. Collaborative training of medical artificial intelligence models with non-uniform labels. *Sci Rep* 13, 6046 (2023). https://doi.org/10.1038/s41598-023-33303-y

[3] Tayebi Arasteh, Soroosh, Mahshad Lotfinia, Teresa Nolte, Marwin-Jonathan Sähn, Peter Isfort, Christiane Kuhl, Sven Nebelung, Georgios Kaissis, and Daniel Truhn. "Securing collaborative medical AI by using differential privacy: domain transfer for classification of chest radiographs." *Radiology: Artificial Intelligence* 6, no. 1 (2023): e230212.

[4] Tayebi Arasteh, S., Misera, L., Kather, J.N. et al. Enhancing diagnostic deep learning via self-supervised pretraining on large-scale, unlabeled non-medical images. *Eur Radiol Exp* 8, 10 (2024). https://doi.org/10.1186/s41747-023-00411-3

[5] Tayebi Arasteh, S., Kuhl, C., Saehn, MJ. et al. Enhancing domain generalization in the AI-based analysis of chest radiographs with federated learning. *Sci Rep* 13, 22576 (2023). https://doi.org/10.1038/s41598-023-49956-8

[6] Lotfinia, Mahshad, Arash Tayebiarasteh, Samaneh Samiei, Mehdi Joodaki, and Soroosh Tayebi Arasteh. "Boosting multi-demographic federated learning for chest radiograph analysis using general-purpose self-supervised representations." *European Journal of Radiology Artificial Intelligence* 3 (2025): 100028.

Reply to Reviewer #3

General Comment: *"The authors have significantly broadened the scope of their work, compared to the previous submission. The inclusion of new analyses, such as: impact of PEFT, pediatric boundary-condition, computational cost trade-off, and synthetic label-noise provide a valuable insight into the scalability of DINOv3 to the chest x-ray domain. I feel that these changes help distinguish the paper from their previous DINOv2-based analysis, justifying sufficient novelty to not be incremental research."*

Authors' Response: We thank the Reviewer for the constructive assessment of our manuscript.

Reply to Reviewer #4

General Comment: *"I appreciate the authors' substantial revision and the addition of external-validation experiments. However, I still have a concern regarding the primary data-splitting strategy. The manuscript describes patient-wise splits within individual datasets and includes a targeted external validation experiment in which models trained on MIMIC-CXR are evaluated on ChestX-ray14 and CheXpert. While this partly addresses cross-institutional generalization, it does not fully explain why the primary benchmark was designed around within-dataset train/validation/test splits rather than dataset-level holdout splits.*

Even when patient-level leakage is avoided, within-dataset splitting can allow scanner characteristics, acquisition protocols, patient demographics, and label-generation procedures to be shared between training and test subsets. Therefore, the primary benchmark should be interpreted mainly as patient-wise within-dataset generalization, complemented by targeted external validation, rather than as a strict dataset-level generalization assessment. I recommend that the authors explicitly justify this design choice and clarify in the Methods or Discussion how it affects the interpretation of the reported transfer-learning performance."

Authors' Response and Action: We agree with the Reviewer, both with the diagnosis and with the recommended interpretation, and we accept that the previous version did not state the reasoning behind the design. The choice was deliberate, and it follows from what the benchmark was built to measure.

The comparison under study is between initializations, backbones and input resolutions, holding everything else fixed. A dataset-level holdout design does not permit that comparison, for three concrete reasons.

(1) It confounds the factor of interest with the shift between cohorts. Our seven cohorts differ in label provenance (radiologist annotation in Pedi-CXR, VinDr-CXR and UKA-CXR, rule-based natural language processing in ChestX-ray14, CheXpert and MIMIC-CXR, and a hybrid of the two in PadChest), in projection mix (anteroposterior fractions from 0% to 85%), in clinical context and in disease spectrum. Under a dataset-level holdout, a difference between DINOv3 and DINOv2 and a difference between the training and test cohorts enter the same number, and neither can be recovered from it.

(2) The seven cohorts do not share a label space. They carry 3, 11, 14, 17, 10, 10 and 6 labels respectively, only two labels are common to all seven, and even the three adult cohorts used in the external experiment share only seven. A dataset-level holdout benchmark would therefore have to run on a harmonized subset of that size throughout, which discards 10 of the 17 PadChest labels and 7 of the 14 ChestX-ray14 labels. It would also remove the analysis behind one of our main conclusions, namely the finding-group comparison showing that the resolution-linked gains concentrate in small focal and boundary-dependent findings (**Supplementary Table 2**), because the labels carrying that signal are exactly the ones a harmonized subset drops.

(3) The within-dataset design is the one the existing chest radiograph benchmark literature reports under, which keeps our numbers comparable with the values other groups report on these same public cohorts.

We also agree that this design leaves scanner characteristics, acquisition protocols, demographics and labeling procedure shared between the training and test partitions, and that patient-wise splitting removes patient-level leakage but not cohort-level similarity. This is why we added the external experiment in the previous revision, and it is what makes that result informative here: training on MIMIC-CXR and testing on the fully held-out ChestX-ray14 and CheXpert cohorts cost only 0.9 to 2.7 AUROC percentage points at 512×512 pixels, and left the ordering of the five configurations essentially unchanged, with DINOv3 still the strongest initialization for both backbones on both target cohorts (**Table 4**, **Figure 6a** to **d**). Cross-institutional drops for chest radiograph models are well documented and can be far larger than this (1, 2), so a drop of this size says something about how far the within-dataset ranking carries.

We have adopted the Reviewer's wording for how the benchmark should be read, and stated it in both places the Reviewer names. A new paragraph closes the **Experimental design** subsection of the **Methods**:

> "To ensure consistency across experiments, we applied a unified preprocessing workflow to all datasets and kept the train, validation, and test partitions fixed throughout the study. The primary benchmark was deliberately designed around patient-wise within-dataset splits and not around dataset-level holdout splits, because its purpose was to compare initialization strategies, backbone families, and input resolutions under otherwise identical conditions. Under a dataset-level holdout design, the difference between initializations and the shift between cohorts would enter the same estimate, and the seven cohorts differ in label provenance, projection mix, clinical context, and disease spectrum. Such a design would also require a label space common to all cohorts, whereas the label sets range from 3 to 17 labels per dataset and only two labels are common to all seven, so most of the per-label detail the benchmark relies on would be lost. Patient-wise splitting removes patient-level leakage, but scanner characteristics, acquisition protocols, patient demographics, and label-generation procedures remain shared between the training and test partitions of a given cohort. The main benchmark should therefore be read as a measure of patient-wise within-dataset generalization, complemented by the targeted external validation experiment described below, and not as a strict assessment of dataset-level generalization. "

And the paragraph of the **Discussion** that interprets the external-validation experiment now closes:

> "[...] so broader external generalization should still be tested in future work. The two experiments answer different questions and should be read together. The main benchmark establishes how initialization, backbone, and resolution rank under patient-wise within-dataset generalization, where scanner characteristics, acquisition protocols, demographics, and label-generation procedures are shared between the training and test partitions, and the external experiment shows that this ranking carries across institutions at a cost of 0.9 to 2.7 AUROC percentage points. Neither result on its own establishes dataset-level generalization to an arbitrary new cohort. "

References

(1) Zech, J. R., Badgeley, M. A., Liu, M., Costa, A. B., Titano, J. J. & Oermann, E. K. Variable generalization performance of a deep learning model to detect pneumonia in chest radiographs: A cross-sectional study. *PLOS Medicine* **15**, e1002683 (2018).

(2) Tayebi Arasteh, S. *et al.* Enhancing domain generalization in the AI-based analysis of chest radiographs with federated learning. *Scientific Reports* **13**, 22576 (2023).
