# Supplementary material for: Resolution-dependent self-supervised transfer in chest radiograph classification
Source: Commun Med (Lond). 2026 Sep 9;6:479. doi: 10.1038/s43856-026-01897-9 (PMC13558615; doi:10.1038/s43856-026-01897-9)
Supplement: Supplementary file 2 [file 43856_2026_1897_MOESM2_ESM.pdf]

Resolution-dependent self-supervised transfer in chest radiograph classification – Supplementary information

Soroosh Tayebi Arasteh (1,2,3,4), Mina Shaigan (5), Christiane Kuhl (2), Jakob Nikolas Kather (6,7,8), Sven Nebelung* (1,2), Daniel Truhn* (1,2)

(1) Lab for AI in Medicine, RWTH Aachen University, Aachen, Germany.
(2) Department of Diagnostic and Interventional Radiology, University Hospital RWTH Aachen, Aachen, Germany.
(3) Department of Urology, Stanford University, Stanford, CA, USA.
(4) Department of Radiology, Stanford University, Stanford, CA, USA.
(5) Institute for Computational Genomics, Joint Research Center for Computational Biomedicine, University Hospital RWTH Aachen, Aachen, Germany.
(6) Else Kroener Fresenius Center for Digital Health, Technical University Dresden, Dresden, Germany.
(7) Department of Medicine I, University Hospital Dresden, Dresden, Germany.
(8) National Center for Tumor Diseases (NCT), University Hospital Heidelberg, Heidelberg, Germany.

* Sven Nebelung and Daniel Truhn are shared senior authors.

Correspondence
Soroosh Tayebi Arasteh, Dr.-Ing., Dr. rer. medic.
Lab for AI in Medicine, Department of Diagnostic and Interventional Radiology
University Hospital RWTH Aachen
Pauwelsstr. 30
52074 Aachen, Germany
Email: soroosh.arasteh@rwth-aachen.de

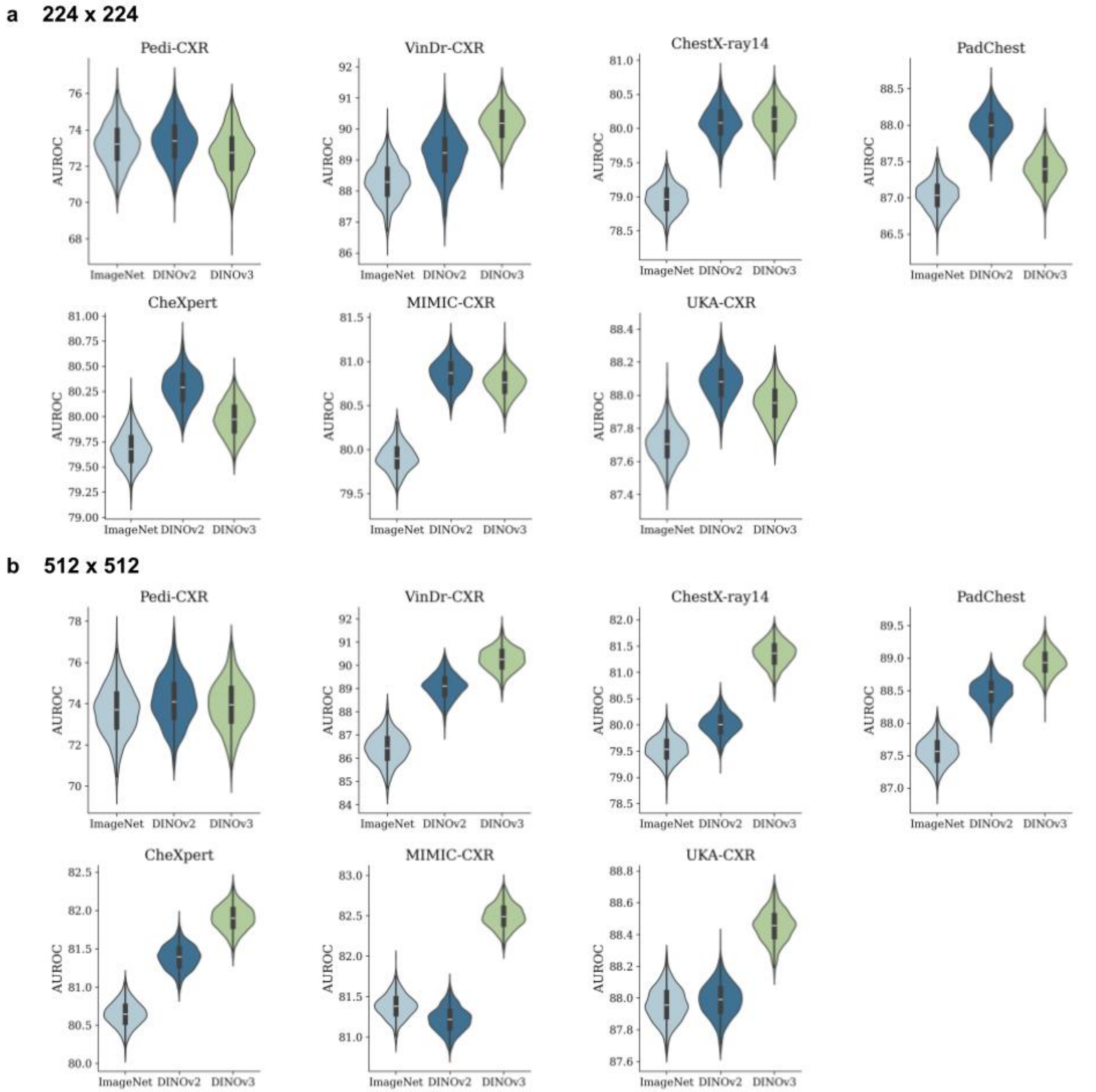

Supplementary Figure 1: Overall performance distributions across datasets. **a** Violin plots of bootstrap distributions (n = 1,000 resamples) for average AUROC values across all labels, comparing ImageNet, DINOv2, and DINOv3 initializations at 224 × 224 resolution with the ViT-B/16 backbone. At this resolution, DINOv2 often retained a slight edge, with DINOv3 performing comparably. **b** Corresponding bootstrap distributions at 512 × 512 resolution. Results are shown for all seven datasets: Pedi-CXR (training n=6,955; test n=1,397), VinDr-CXR (training n=15,000; test n=3,000), ChestX-ray14 (training n=77,870; validation n=8,654; test n=25,596), PadChest (training n = 79,697; validation n=8,783; test n=22,045), CheXpert (training n=115,449; validation n=13,098; test n=29,318), MIMIC-CXR (training n=153,255; validation n=18,139; test n=43,793), and UKA-CXR (training n=137,902; validation n=15,353; test n=40,106). Each violin shows the full distribution of the mean AUROC over the 1,000 bootstrap resamples of the test set of that dataset: Pedi-CXR (n = 1,397), VinDr-CXR (n = 3,000), ChestX-ray14 (n = 25,596), PadChest (n = 22,045), CheXpert (n = 29,318), MIMIC-CXR (n = 43,793) and UKA-CXR (n = 40,106).

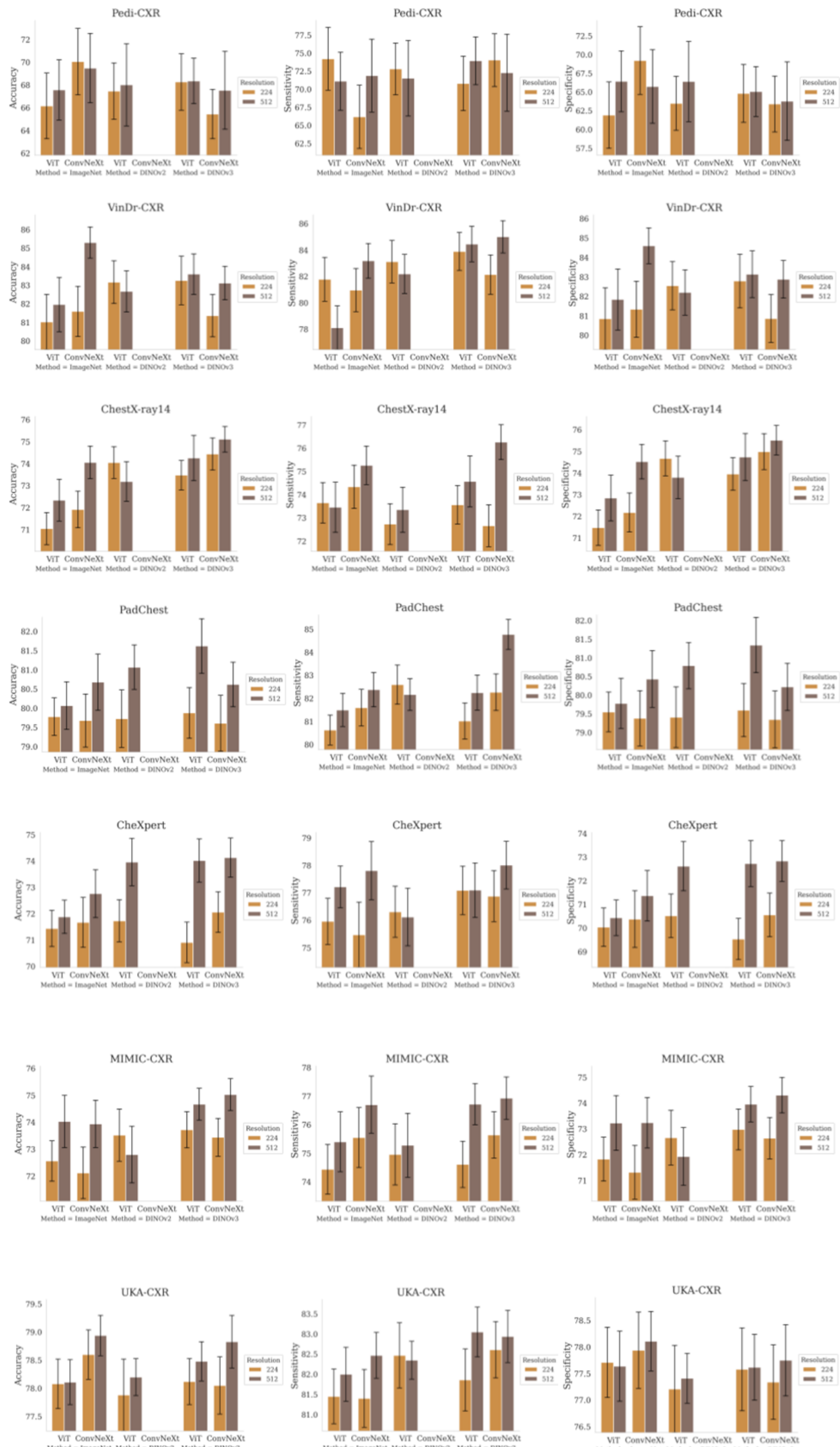

Supplementary Figure 2: Accuracy, sensitivity, and specificity across initialization strategies. Each panel corresponds to one dataset, showing bar plots for ImageNet, DINOv2, and DINOv3 at 224 × 224 and 512 × 512 resolutions. Bars display mean values with standard deviations from 1,000 bootstrap resamples. Each bar is the mean over 1,000 bootstrap resamples of the test set of that dataset (Pedi-CXR (n = 1,397), VinDr-CXR (n = 3,000), ChestX-ray14 (n = 25,596), PadChest (n = 22,045), CheXpert (n = 29,318), MIMIC-CXR (n = 43,793) and UKA-CXR (n = 40,106)), and the error bars show one standard deviation of that bootstrap distribution.

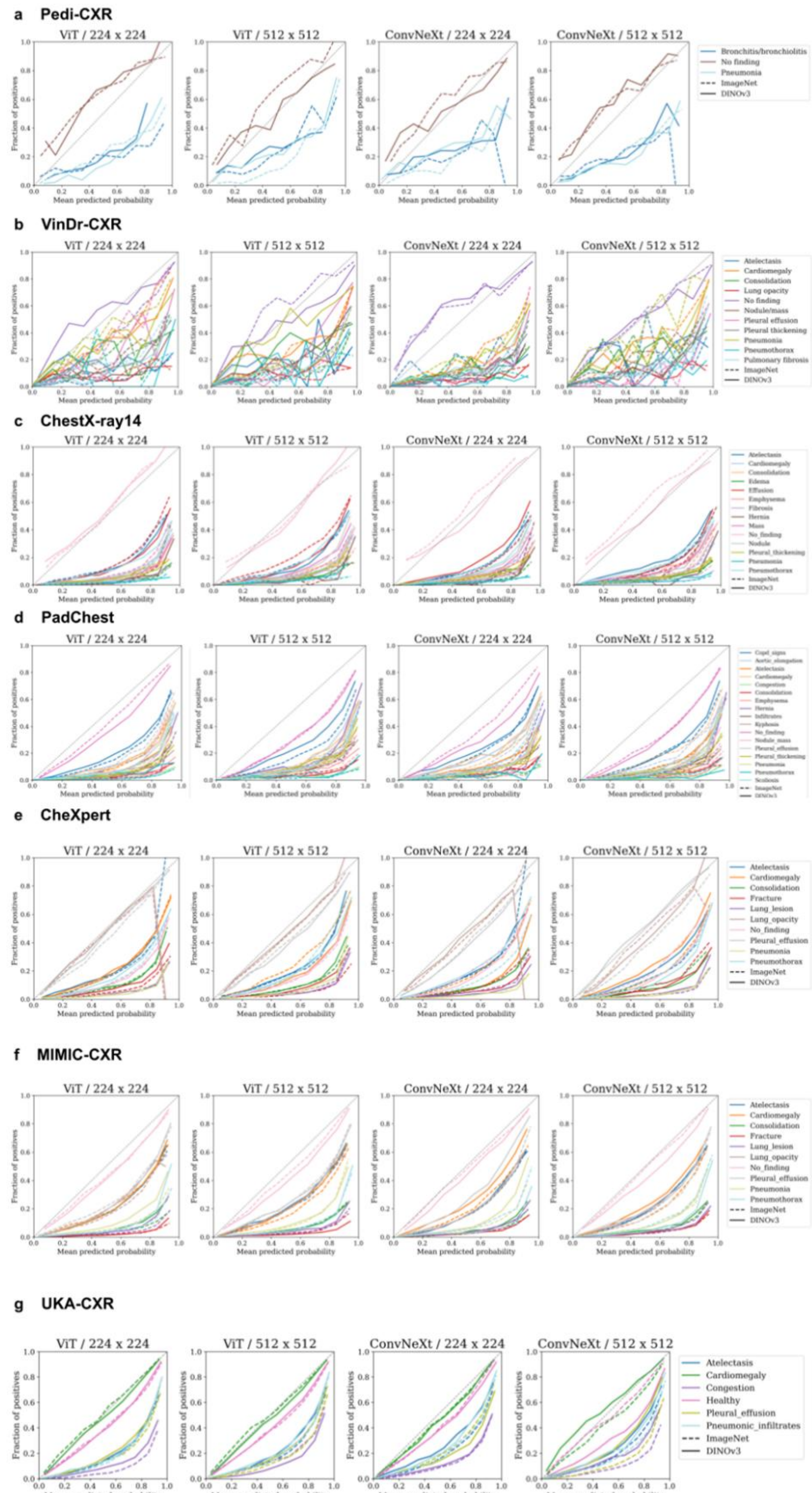

Supplementary Figure 3: Calibration analysis. Reliability diagrams comparing expected vs. observed probabilities for ImageNet and DINOv3 initializations under full finetuning. Plots are stratified by dataset (rows) and resolution (224 × 224, 512 × 512; columns), with separate panels for ViT-B/16 **(a)** and ConvNeXt-B **(b)**. The diagonal line indicates perfect calibration. Results are averaged across all labels within each dataset, with bootstrapped confidence shading from 1,000 resamples. The center of each reliability curve is the mean over 1,000 bootstrap resamples of the test set

of that dataset, and the shading shows the 95% confidence interval, taken as the 2.5th and 97.5th percentiles of the same bootstrap distribution.

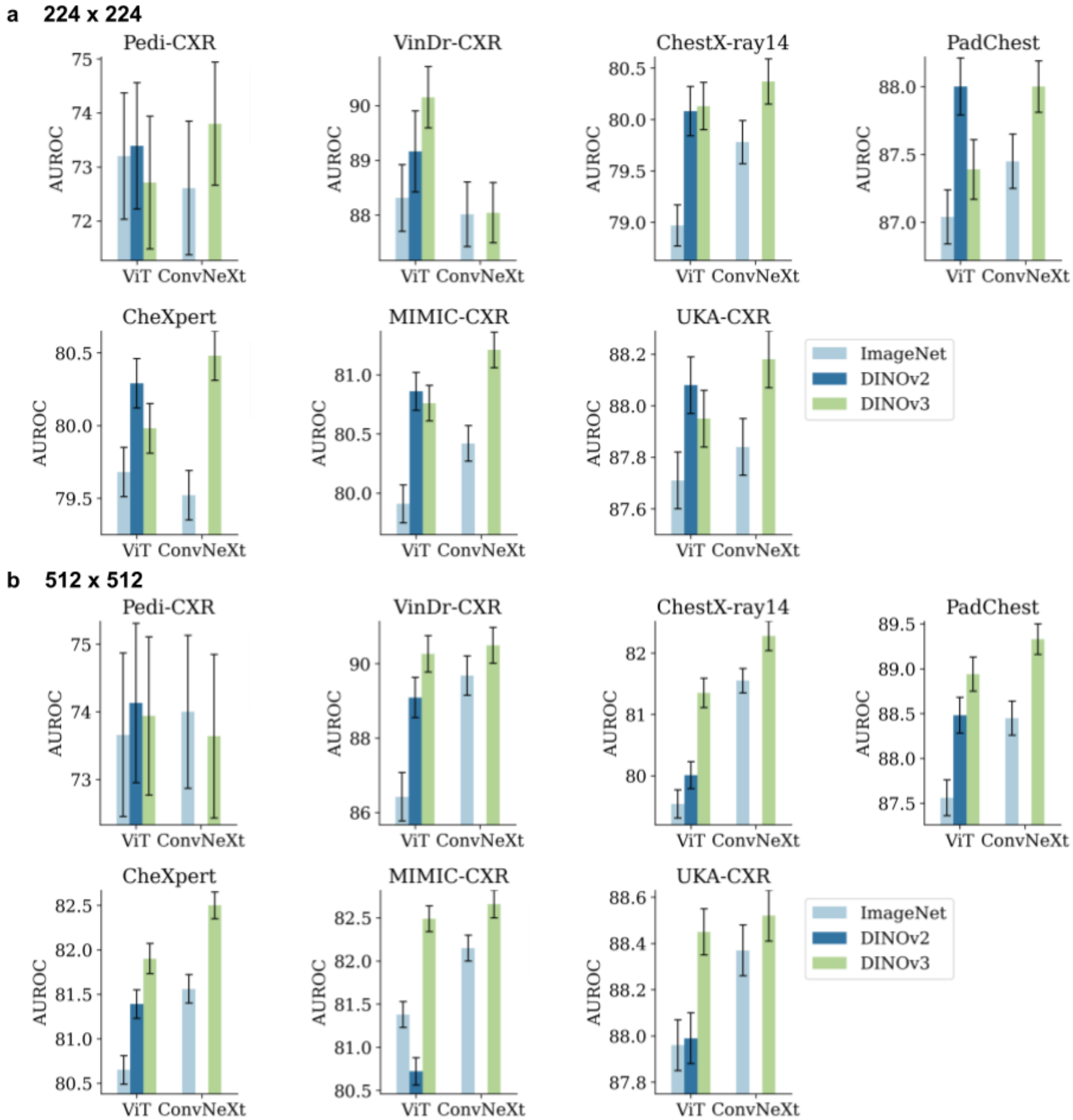

Supplementary Figure 4: Backbone comparison across datasets. **(a)** Mean AUROC values across all labels with standard deviations from 1,000 bootstrap resamples for ViT-B/16 and ConvNeXt-B backbones at 224 × 224 resolution. Results for ImageNet, DINOv2, and DINOv3 initializations are shown side by side within each backbone. **(b)** Corresponding results at 512 × 512 resolution. Results are shown for all seven datasets: Pedi-CXR (training n=6,955; test n=1,397), VinDr-CXR (training n=15,000; test n=3,000), ChestX-ray14 (training n=77,870; validation n=8,654; test n=25,596), PadChest (training n = 79,697; validation n=8,783; test n=22,045), CheXpert (training n=115,449; validation n=13,098; test n=29,318), MIMIC-CXR (training n=153,255; validation n=18,139; test n=43,793), and UKA-CXR (training n=137,902; validation n=15,353; test n=40,106). Each bar is the mean over 1,000 bootstrap resamples of the test set of that dataset (Pedi-CXR (n = 1,397), VinDr-CXR (n = 3,000), ChestX-ray14 (n = 25,596), PadChest (n = 22,045), CheXpert (n = 29,318), MIMIC-CXR (n = 43,793) and UKA-CXR (n = 40,106)), and the error bars show one standard deviation of that bootstrap distribution.

a 224 x 224

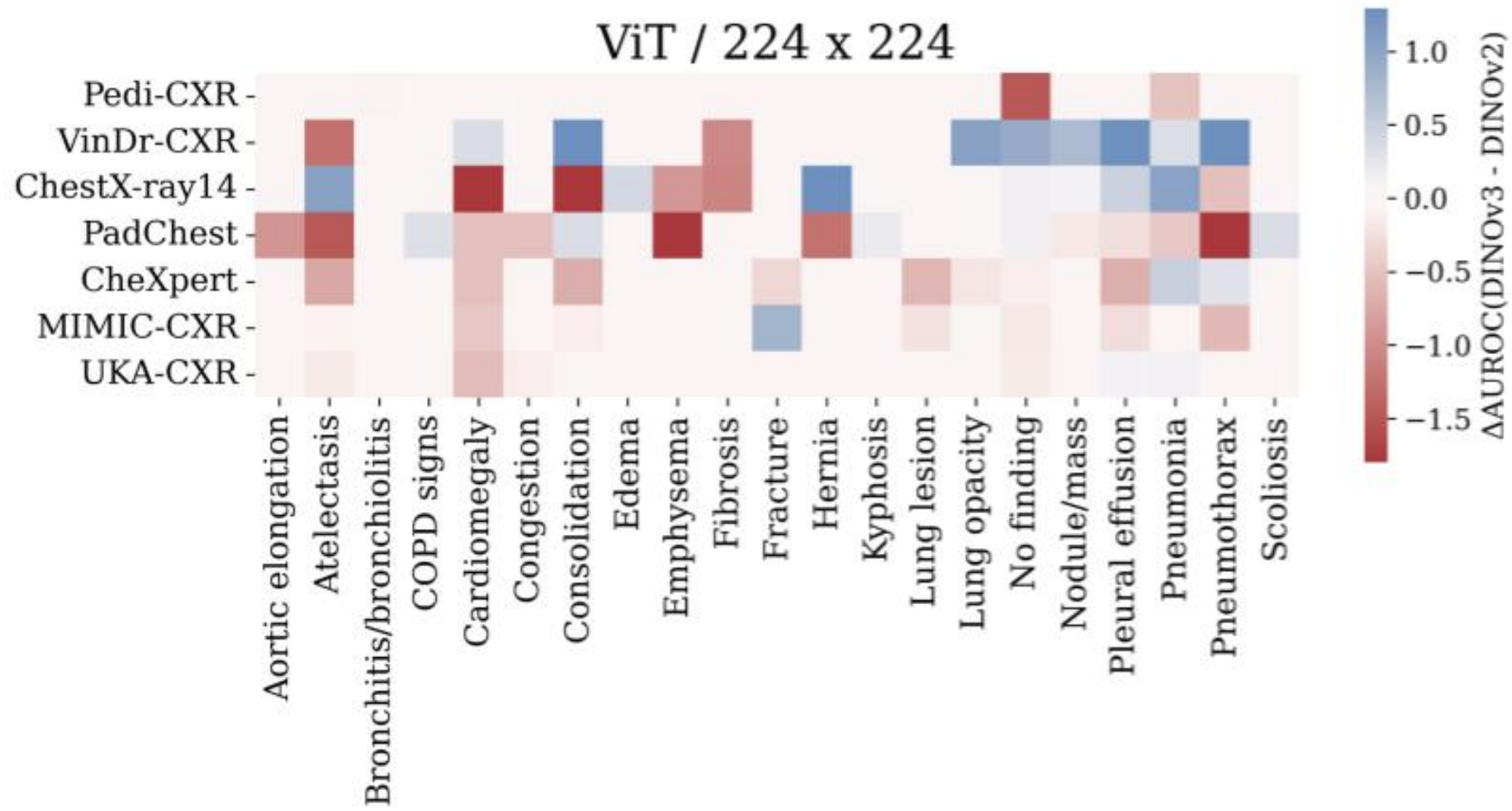

b 512 x 512

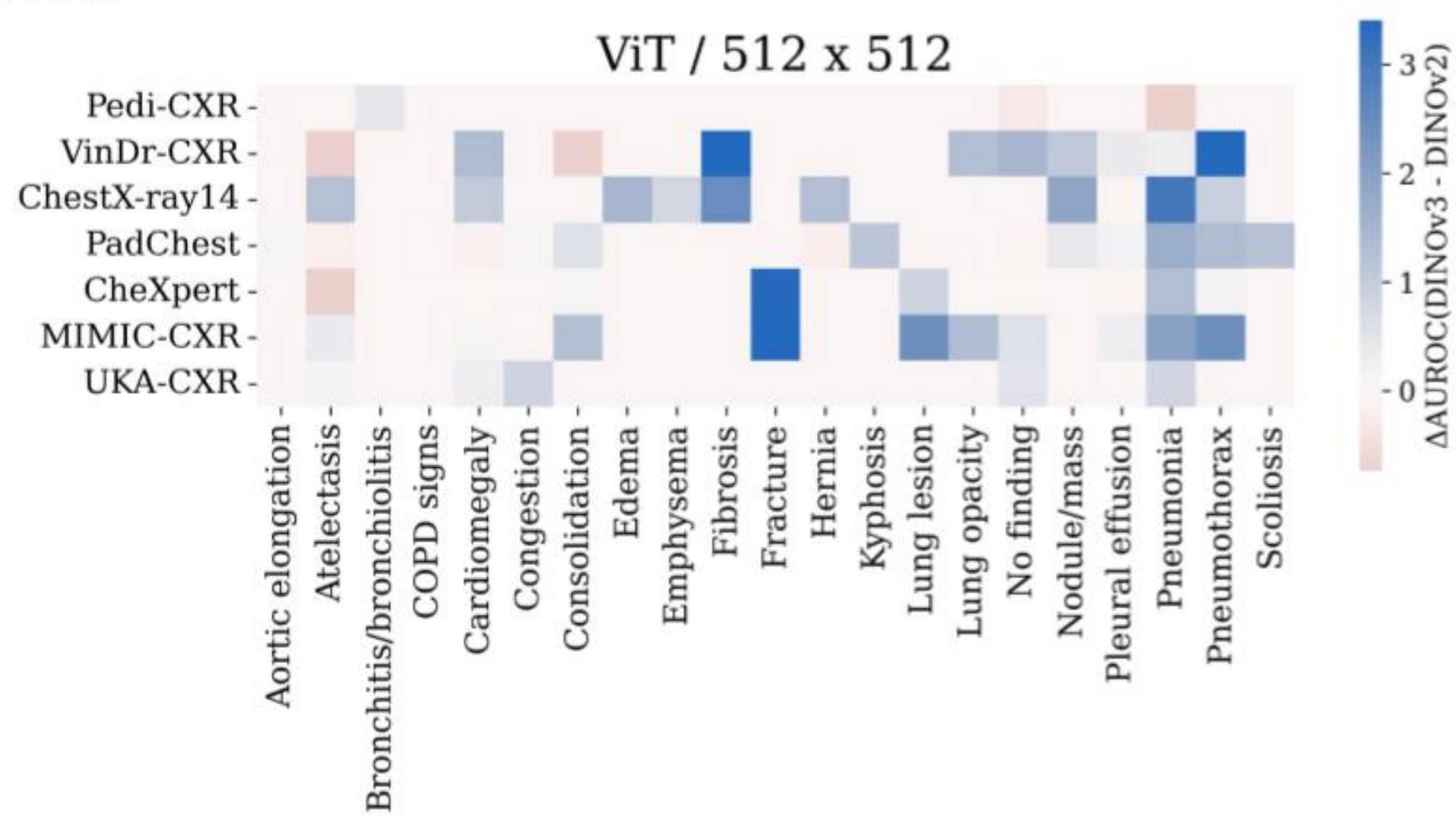

Supplementary Figure 5: Label-wise performance analysis. **a** Heatmap of differences in AUROC (ΔAUROC) between DINOv3 and DINOv2 across all labels and datasets at 224 × 224 resolution. Results are shown for all seven datasets: Pedi-CXR (training n=6,955; test n=1,397), VinDr-CXR (training n=15,000; test n=3,000), ChestX-ray14 (training n=77,870; validation n=8,654; test n=25,596), PadChest (training n = 79,697; validation n=8,783; test n=22,045), CheXpert (training n=115,449; validation n=13,098; test n=29,318), MIMIC-CXR (training n=153,255; validation n=18,139; test n=43,793), and UKA-CXR (training n=137,902; validation n=15,353; test n=40,106). Each cell is the difference of two bootstrap means over the 1,000 resamples of the test set of that dataset, and no error bars are shown.

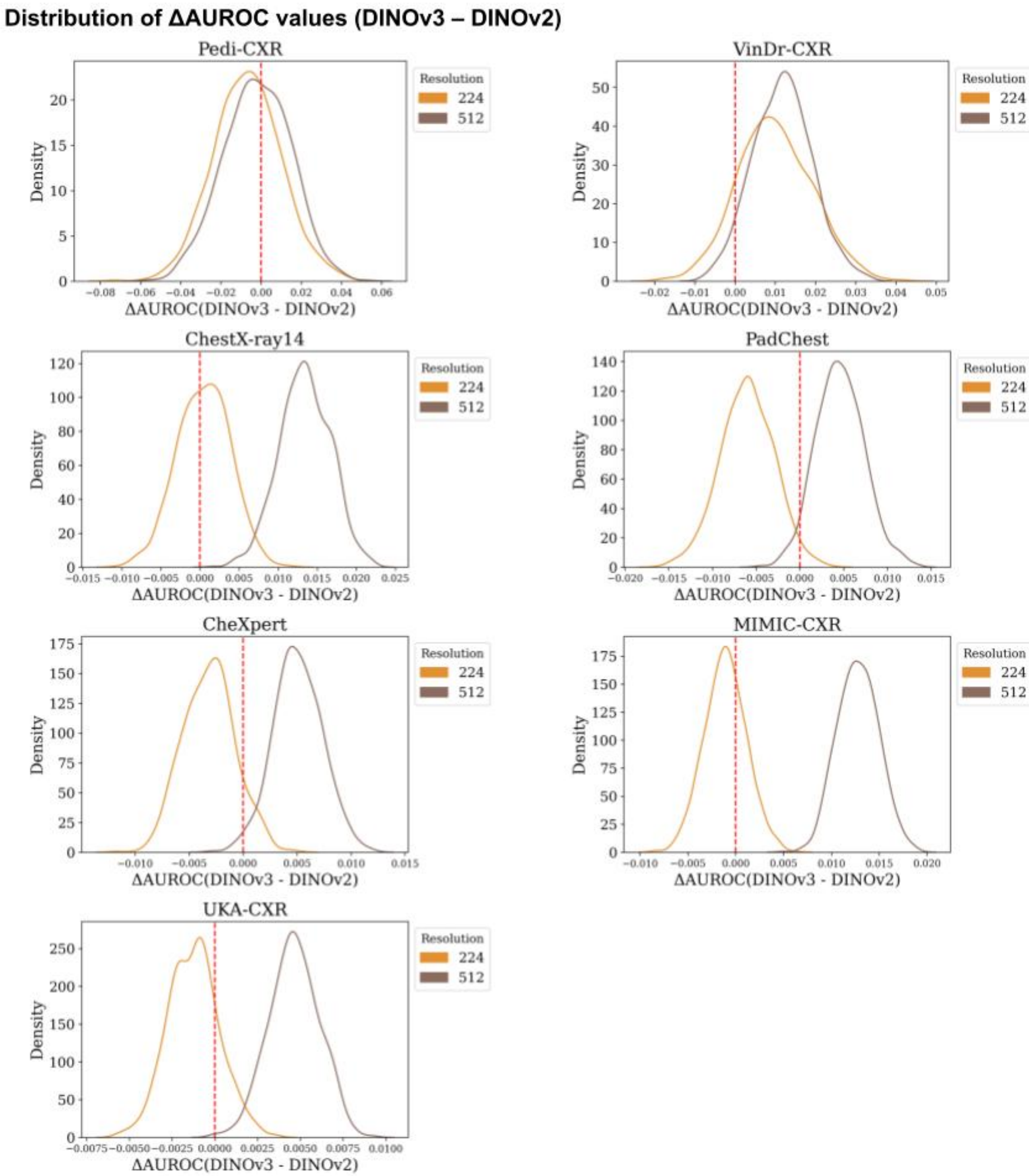

Supplementary Figure 6: Distribution of ΔAUROC values (DINOv3 – DINOv2) for the ViT-B backbone. Kernel density plots show bootstrap-sampled differences in average AUROC (n = 1,000 resamples) across all labels for each dataset, comparing 224 × 224 and 512 × 512 resolutions. A vertical dashed line at zero indicates no difference between methods. Results are shown for all seven datasets: Pedi-CXR (training n=6,955; test n=1,397), VinDr-CXR (training n=15,000; test n=3,000), ChestX-ray14 (training n=77,870; validation n=8,654; test n=25,596), PadChest (training n = 79,697; validation n=8,783; test n=22,045), CheXpert (training n=115,449; validation n=13,098; test n=29,318), MIMIC-CXR (training n=153,255; validation n=18,139; test n=43,793), and UKA-CXR (training n=137,902; validation n=15,353; test n=40,106). Each density is estimated from the 1,000 bootstrap resamples of the test set of that dataset.

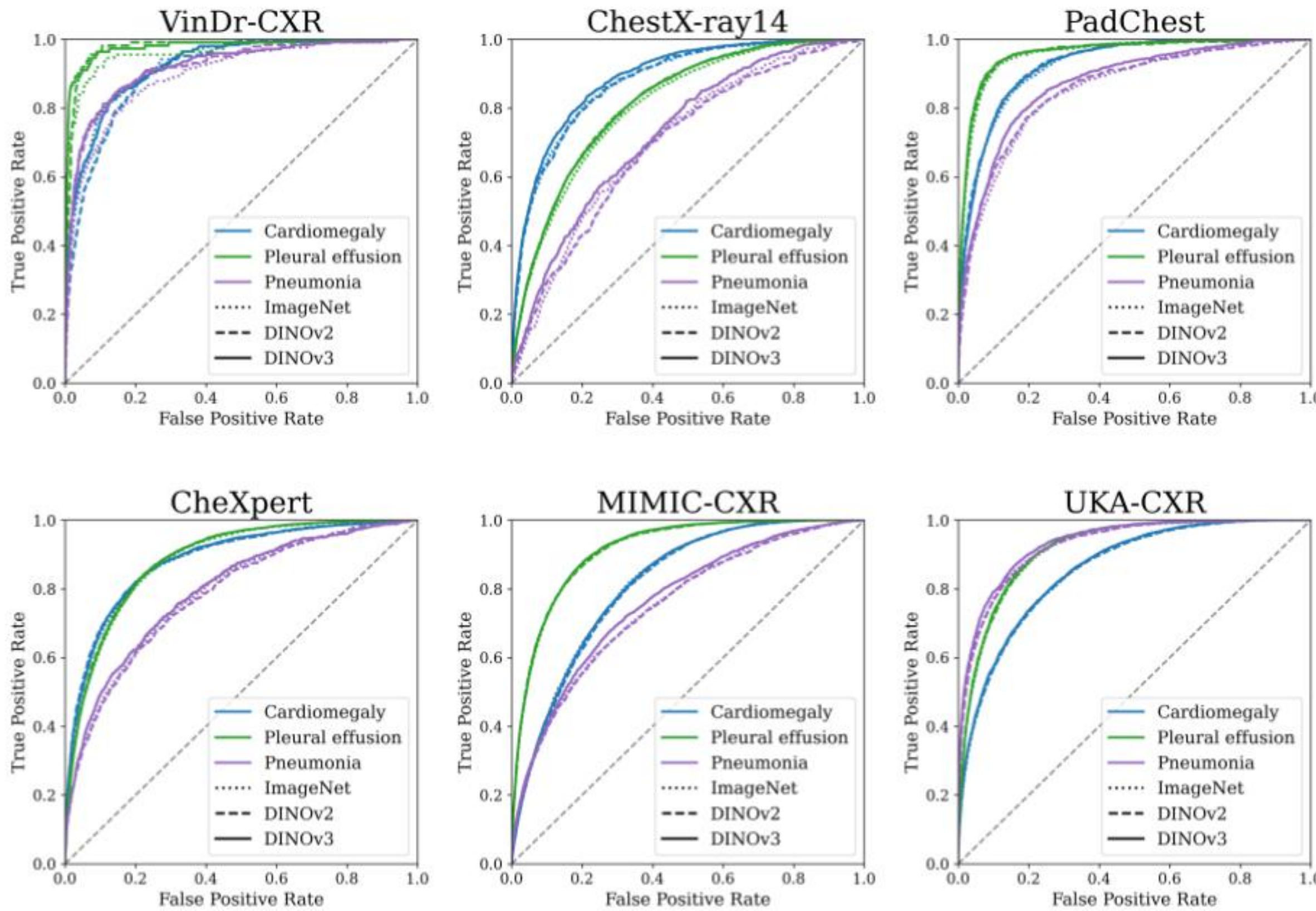

Supplementary Figure 7: Representative receiver operating characteristic (ROC) curves for selected labels (cardiomegaly, pleural effusion, pneumonia) at 512 × 512 resolution. Results are shown for all adult datasets: VinDr-CXR (training n=15,000; test n=3,000), ChestX-ray14 (training n=77,870; validation n=8,654; test n=25,596), PadChest (training n = 79,697; validation n=8,783; test n=22,045), CheXpert (training n=115,449; validation n=13,098; test n=29,318), MIMIC-CXR (training n=153,255; validation n=18,139; test n=43,793), and UKA-CXR (training n=137,902; validation n=15,353; test n=40,106). Each curve is computed on the full test set of that dataset, and no error bars are shown.

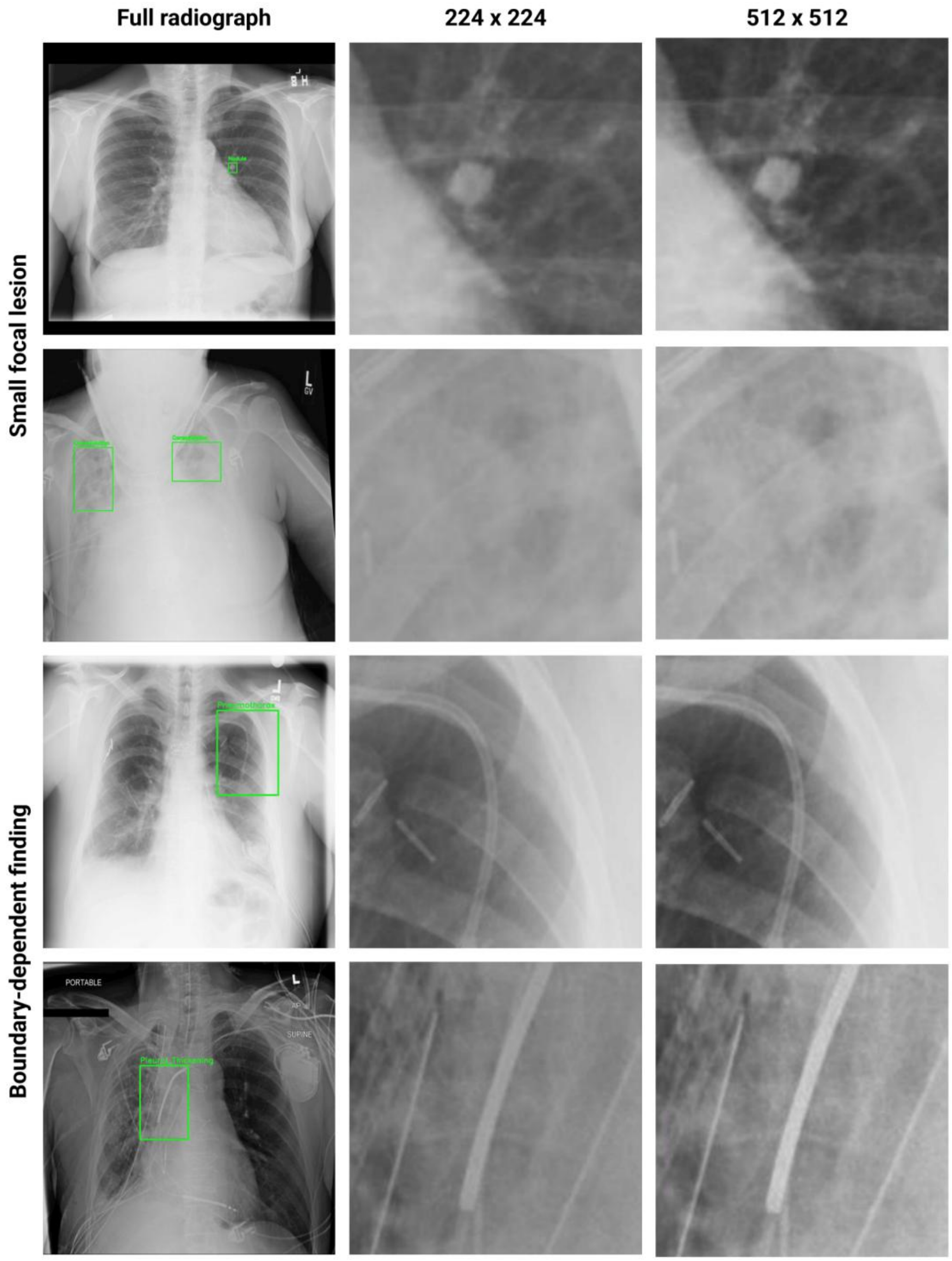

Supplementary Figure 8: Illustrative adult chest radiographs highlighting regions corresponding to findings with the greatest apparent benefit from higher input resolution. Shown are four representative examples from the adult ChestX-ray14 cohort, selected to visualize regions corresponding to small focal and boundary-dependent findings, the two groups that showed the largest average resolution-related gains in the quantitative cross-dataset summary (**Supplementary Table 2**). The left column shows the full radiograph with a manually chosen region of interest and the associated dataset label. The middle and right columns show the corresponding region after standard preprocessing and resizing to 224 × 224 and 512 × 512 pixels, respectively. The cropped regions from the resized images were enlarged to a common display size for visual comparison. Across these illustrative examples, the 512 × 512 versions preserve finer local structure and boundary detail more clearly than the 224 × 224 versions. This figure is intended as a qualitative complement to the quantitative analysis, not as a model-explanation figure and not as a separate performance experiment.

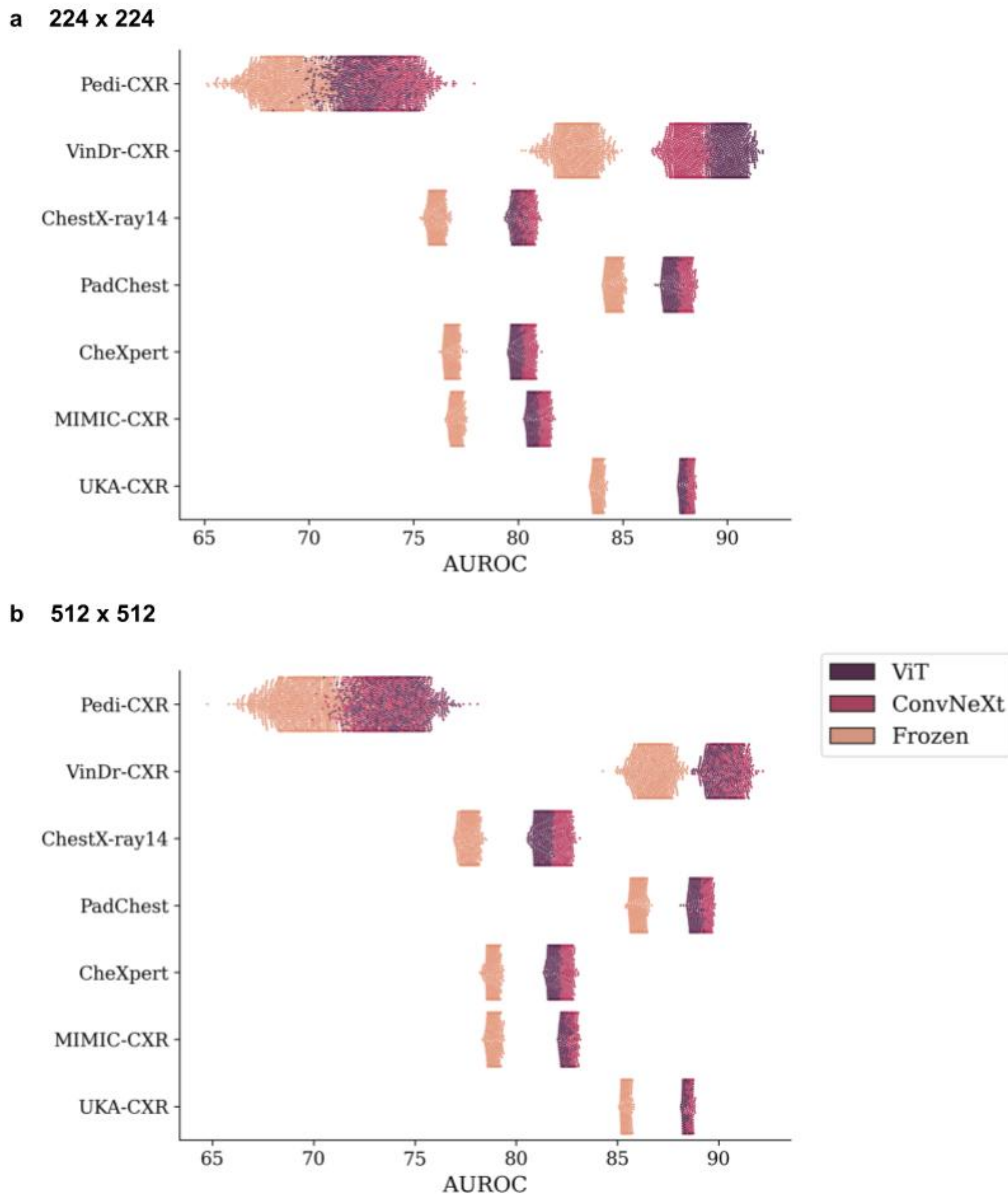

Supplementary Figure 9: Classification using frozen DINOv3-7B features vs. full finetuning of smaller models. Bootstrap distributions of AUROC values (n = 1,000 resamples) comparing classifiers trained on frozen DINOv3-7B features with lightweight heads (~2M parameters) against full finetuning of ViT-B/16 and ConvNeXt-B backbones (~86–87M parameters). Across datasets, full finetuning consistently outperforms frozen representations, despite the much smaller backbone size. Results are shown for all seven datasets: Pedi-CXR (training n=6,955; test n=1,397), VinDr-CXR (training n=15,000; test n=3,000), ChestX-ray14 (training n=77,870; validation n=8,654; test n=25,596), PadChest (training n = 79,697; validation n=8,783; test n=22,045), CheXpert (training n=115,449; validation n=13,098; test n=29,318), MIMIC-CXR (training n=153,255; validation n=18,139; test n=43,793), and UKA-CXR (training n=137,902; validation n=15,353; test n=40,106). Each distribution is the mean AUROC over the 1,000 bootstrap resamples of the test set of that dataset.

Supplementary Table 1: Pairwise bootstrap p-values across datasets, initialization strategies, and input resolutions. Two-sided p-values were obtained from paired bootstrap tests using 1,000 resampled AUROC pairs per model, ensuring identical resampling across initialization strategies for fair comparison. Results are reported separately for ViT-B/16 and ConvNeXt-B under full finetuning, as well as for frozen DINOv3-7B encoders with linear classifiers. Each comparison was performed at 224 × 224, 512 × 512, and, where available, 1024 × 1024 input resolutions. p-values were adjusted for multiple comparisons within coherent families of related tests (e.g., per-resolution comparisons across the six adult datasets) using the Benjamini-Hochberg false discovery rate (FDR) procedure, with FDR-adjusted $p < 0.05$ considered statistically significant. "N/A" indicates configurations that were not evaluated. Results are shown for all seven datasets: Pedi-CXR (training n=6,955; test n=1,397), VinDr-CXR (training n=15,000; test n=3,000), ChestX-ray14 (training n=77,870; validation n=8,654; test n=25,596), PadChest (training n=79,697; validation n=8,783; test n=22,045), CheXpert (training n=115,449; validation n=13,098; test n=29,318), MIMIC-CXR (training n=153,255; validation n=18,139; test n=43,793), and UKA-CXR (training n=137,902; validation n=15,353; test n=40,106).

Backbone/fine tuning	Resolution	Comparison	Pedi-CXR	VinDr-CXR	ChestX-ray14	PadChest	CheXpert	MIMIC-CXR	UKA-CXR
ViT full fine tuning	224 x 224	ImageNet vs. DINOv3	0.31	0.006	0.006	0.012	0.006	0.006	0.006
		ImageNet vs. DINOv2	0.61	0.11	0.006	0.006	0.006	0.006	0.006
		DINOv3 vs. DINOv2	0.78	0.050	0.40	0.003	0.003	0.20	0.008
	512 x 512	ImageNet vs. DINOv3	0.37	0.006	0.006	0.006	0.006	0.006	0.006
		ImageNet vs. DINOv2	0.32	0.006	0.006	0.006	0.006	0.044	0.26
		DINOv3 vs. DINOv2	0.54	0.019	0.006	0.006	0.006	0.006	0.006
	1024 x 1024	ImageNet vs. DINOv3	0.001	N/A	0.002	N/A	N/A	0.002	N/A
		ImageNet vs. DINOv2	0.001	N/A	0.002	N/A	N/A	0.40	N/A
		DINOv3 vs. DINOv2	0.001	N/A	0.002	N/A	N/A	0.002	N/A
ConvNeXt full fine tuning	224 x 224	ImageNet vs. DINOv3	0.080	0.45	0.001	0.001	0.001	0.001	0.001
	512 x 512	ImageNet vs. DINOv3	0.66	0.013	0.001	0.001	0.001	0.001	0.001
	1024 x 1024	ImageNet vs. DINOv3	0.76	N/A	0.002	N/A	N/A	0.038	N/A
ViT frozen features	224 x 224	Frozen vs. DINOv3	0.001	0.006	0.006	0.006	0.006	0.006	0.006
		Frozen vs. DINOv2	0.001	0.006	0.006	0.006	0.006	0.006	0.006
		Frozen vs. ImageNet	0.001	0.006	0.006	0.006	0.006	0.006	0.006
	512 x 512	Frozen vs. DINOv3	0.001	0.006	0.006	0.006	0.006	0.006	0.006
		Frozen vs. DINOv2	0.001	0.006	0.006	0.006	0.006	0.006	0.006
		Frozen vs. ImageNet	0.001	0.23	0.006	0.006	0.006	0.006	0.006
ConvNeXt frozen features	224 x 224	Frozen vs. DINOv3	0.001	0.006	0.006	0.006	0.006	0.006	0.006
		Frozen vs. ImageNet	0.001	0.006	0.006	0.006	0.006	0.006	0.006
	512 x 512	Frozen vs. DINOv3	0.001	0.006	0.006	0.006	0.006	0.006	0.006
		Frozen vs. ImageNet	0.001	0.006	0.006	0.006	0.006	0.006	0.006

Supplementary Table 2: Compact cross-dataset per-label and per-finding-group summary. Summary results are shown for selected clinically important labels and grouped abnormality categories across the evaluated datasets. For each row, the table reports the datasets contributing to the summary, the best observed AUROC at 224 × 224 and 512 × 512 resolution, the corresponding change in AUROC between resolutions, and the difference between DINOv3 and DINOv2 at 224 × 224 and 512 × 512. AUROC values are reported as mean ± SD [95% CI] across contributing datasets. For each dataset and resolution, the best AUROC denotes the highest observed value among the evaluated model configurations. Differences between DINOv3 and DINOv2 were computed using architecture-matched ViT-B models, as DINOv2 results were available only for ViT-B. For grouped rows, clinically harmonized labels were first averaged within each dataset and then summarized across datasets.

Label or finding group	Label group category	Datasets included	Best AUROC at 224 × 224	Best AUROC at 512 × 512	ΔAUROC (best 512 − best 224)	ΔAUROC (DINOv3 − DINOv2 at 224 × 224)	ΔAUROC (DINOv3 − DINOv2 at 512 × 512)
Cardiomegaly	Large-structure	UKA-CXR, CheXpert, ChestX-ray14, MIMIC-CXR, PadChest, VinDr-CXR	88.7 ± 4.7 [83.8, 93.6]	88.7 ± 4.4 [84.1, 93.4]	0.0 ± 0.5 [-0.5, 0.5]	-0.7 ± 0.9 [-1.6, 0.3]	0.5 ± 0.6 [-0.2, 1.1]
Pleural effusion	Large-structure	UKA-CXR, CheXpert, ChestX-ray14, MIMIC-CXR, PadChest, VinDr-CXR	91.2 ± 5.6 [85.3, 97.1]	91.6 ± 5.3 [86.1, 97.2]	0.4 ± 0.4 [0.0, 0.9]	0.1 ± 0.7 [-0.6, 0.8]	0.1 ± 0.2 [-0.1, 0.3]
Pneumonia	Diffuse / textural	UKA-CXR, CheXpert, ChestX-ray14, MIMIC-CXR, PadChest, Pediatric CXR, VinDr-CXR	82.5 ± 8.4 [74.7, 90.3]	83.7 ± 7.9 [76.4, 91.0]	1.2 ± 0.6 [0.7, 1.7]	0.1 ± 0.5 [-0.4, 0.6]	1.1 ± 1.2 [0.1, 2.2]
Atelectasis	Diffuse / textural	UKA-CXR, CheXpert, ChestX-ray14, MIMIC-CXR, PadChest, VinDr-CXR	81.1 ± 6.7 [74.1, 88.1]	82.7 ± 6.9 [75.5, 89.9]	1.6 ± 1.0 [0.5, 2.7]	-0.4 ± 0.9 [-1.4, 0.5]	-0.3 ± 1.2 [-1.5, 1.0]
Consolidation	Diffuse / textural	CheXpert, ChestX-ray14, MIMIC-CXR, PadChest, VinDr-CXR	82.8 ± 7.9 [72.9, 92.7]	83.7 ± 8.2 [73.5, 93.9]	0.9 ± 0.4 [0.4, 1.4]	-0.1 ± 1.3 [-1.7, 1.5]	-0.2 ± 1.4 [-2.0, 1.6]
Pneumothorax	Boundary-dependent	CheXpert, ChestX-ray14, MIMIC-CXR, PadChest, VinDr-CXR	89.2 ± 2.8 [85.7, 92.6]	91.5 ± 3.1 [87.6, 95.3]	2.3 ± 0.9 [1.2, 3.4]	0.2 ± 2.5 [-2.9, 3.3]	1.8 ± 1.7 [-0.3, 4.0]
No finding	Normal / reference	UKA-CXR, CheXpert, ChestX-ray14, MIMIC-CXR, PadChest	84.2 ± 6.3 [76.4, 92.0]	84.8 ± 5.9 [77.5, 92.1]	0.6 ± 0.4 [0.1, 1.2]	-0.0 ± 0.2 [-0.2, 0.2]	0.1 ± 0.3 [-0.2, 0.5]
Small focal lesions	Small focal	CheXpert, MIMIC-CXR, ChestX-ray14, PadChest, VinDr-CXR	78.6 ± 3.9 [73.7, 83.4]	82.1 ± 3.1 [78.3, 85.9]	3.5 ± 1.1 [2.2, 4.9]	0.0 ± 0.6 [-0.6, 0.7]	1.2 ± 0.6 [0.4, 2.0]
Boundary-dependent findings	Boundary-dependent	CheXpert, MIMIC-CXR, ChestX-ray14, PadChest, VinDr-CXR	87.2 ± 3.7 [82.7, 91.8]	89.6 ± 3.8 [84.9, 94.3]	2.3 ± 0.7 [1.4, 3.3]	0.3 ± 1.9 [-2.1, 2.7]	1.7 ± 1.5 [-0.2, 3.5]
Diffuse / textural findings	Diffuse / textural	UKA-CXR, CheXpert, ChestX-ray14, MIMIC-CXR, PadChest, Pediatric CXR, VinDr-CXR	81.7 ± 6.3 [75.8, 87.6]	82.6 ± 6.3 [76.8, 88.4]	0.9 ± 0.4 [0.5, 1.3]	-0.2 ± 0.2 [-0.4, 0.0]	0.5 ± 0.6 [0.0, 1.1]
Large-structure findings	Large-structure	UKA-CXR, CheXpert, ChestX-ray14, MIMIC-CXR, PadChest, VinDr-CXR	89.9 ± 4.4 [85.4, 94.5]	90.2 ± 4.1 [85.9, 94.5]	0.2 ± 0.3 [-0.1, 0.6]	-0.3 ± 0.6 [-0.9, 0.3]	0.3 ± 0.3 [-0.1, 0.7]

Supplementary Table 3: Relative computational scaling across resolution transitions, backbones, and initialization strategies. Summary of computational scaling for all full fine-tuning experiments in this study, expressed as resolution-transition ratios. For each transition, metric, backbone group, and initialization group, the table reports the mean ratio ± standard deviation (SD), median ratio, minimum ratio, maximum ratio, and the number of evaluated configurations (n). Ratios are shown for both per-epoch training time and total wall-clock training time to convergence. DINOv2 ratios are reported only for ViT-B/16, as DINOv2 was not evaluated with ConvNeXt-B. Results are shown for all seven datasets: Pedi-CXR (training n=6,955; test n=1,397), VinDr-CXR (training n=15,000; test n=3,000), ChestX-ray14 (training n=77,870; validation n=8,654; test n=25,596), PadChest (training n=79,697; validation n=8,783; test n=22,045), CheXpert (training n=115,449; validation n=13,098; test n=29,318), MIMIC-CXR (training n=153,255; validation n=18,139; test n=43,793), and UKA-CXR (training n=137,902; validation n=15,353; test n=40,106).

Transition	Metric	Backbone	Initialization	Ratio (mean ± SD)	Ratio (Median	Ratio (Minimum)	Ratio (Maximum)	n
224 → 512	Epoch time	All	All	3.3 ± 1.3	2.8	1.6	7.0	35
224 → 512	Epoch time	ViT-B/16	All	3.5 ± 1.4	3.0	1.6	7.0	21
224 → 512	Epoch time	ConvNeXt-B	All	3.0 ± 1.0	2.5	2.1	4.8	14
224 → 512	Epoch time	ViT-B/16	ImageNet	3.1 ± 1.2	3.0	1.6	5.2	7
224 → 512	Epoch time	ViT-B/16	DINOv2	3.7 ± 1.8	2.8	2.4	7.0	7
224 → 512	Epoch time	ViT-B/16	DINOv3	3.7 ± 1.3	3.1	2.7	6.2	7
224 → 512	Epoch time	ConvNeXt-B	ImageNet	2.9 ± 0.9	2.5	2.2	4.8	7
224 → 512	Epoch time	ConvNeXt-B	DINOv3	3.0 ± 1.1	2.6	2.1	4.8	7
224 → 512	Total time to converge	All	All	3.6 ± 1.5	3.5	0.6	7.9	35
224 → 512	Total time to converge	ViT-B/16	All	4.0 ± 1.6	4.1	0.6	7.9	21
224 → 512	Total time to converge	ConvNeXt-B	All	3.0 ± 1.3	2.8	1.4	4.8	14
224 → 512	Total time to converge	ViT-B/16	ImageNet	4.4 ± 1.4	4.9	1.8	5.7	7
224 → 512	Total time to converge	ViT-B/16	DINOv2	4.1 ± 2.3	4.1	0.6	7.9	7
224 → 512	Total time to converge	ViT-B/16	DINOv3	3.6 ± 0.7	3.5	2.5	4.5	7
224 → 512	Total time to converge	ConvNeXt-B	ImageNet	2.7 ± 1.2	2.7	1.5	4.8	7
224 → 512	Total time to converge	ConvNeXt-B	DINOv3	3.4 ± 1.4	4.0	1.4	4.8	7
512 → 1024	Epoch time	All	All	6.2 ± 4.4	5.1	2.4	18.8	15
512 → 1024	Epoch time	ViT-B/16	All	7.9 ± 5.1	6.0	3.2	18.8	9
512 → 1024	Epoch time	ConvNeXt-B	All	3.6 ± 0.9	3.6	2.4	5.1	6
512 → 1024	Epoch time	ViT-B/16	ImageNet	9.3 ± 8.3	6.0	3.2	18.8	3
512 → 1024	Epoch time	ViT-B/16	DINOv2	6.2 ± 2.4	6.5	3.7	8.3	3
512 → 1024	Epoch time	ViT-B/16	DINOv3	8.1 ± 4.5	5.7	5.4	13.3	3
512 → 1024	Epoch time	ConvNeXt-B	ImageNet	4.2 ± 0.8	3.7	3.7	5.1	3
512 → 1024	Epoch time	ConvNeXt-B	DINOv3	3.1 ± 0.6	3.3	2.4	3.5	3
512 → 1024	Total time to converge	All	All	7.7 ± 5.8	4.8	2.2	18.9	15
512 → 1024	Total time to converge	ViT-B/16	All	10.6 ± 5.8	8.9	4.7	18.9	9
512 → 1024	Total time to converge	ConvNeXt-B	All	3.3 ± 1.1	2.9	2.2	4.8	6
512 → 1024	Total time to converge	ViT-B/16	ImageNet	11.1 ± 5.5	14.0	4.8	14.6	3
512 → 1024	Total time to converge	ViT-B/16	DINOv2	13.9 ± 7.9	18.0	4.7	18.9	3
512 → 1024	Total time to converge	ViT-B/16	DINOv3	6.7 ± 1.9	5.7	5.4	8.9	3
512 → 1024	Total time to converge	ConvNeXt-B	ImageNet	3.1 ± 1.1	2.8	2.2	4.4	3
512 → 1024	Total time to converge	ConvNeXt-B	DINOv3	3.5 ± 1.2	3.0	2.5	4.8	3
224 → 1024	Epoch time	All	All	17.4 ± 10.3	16.4	7.2	40.4	15
224 → 1024	Epoch time	ViT-B/16	All	22.4 ± 10.6	18.2	9.6	40.4	9
224 → 1024	Epoch time	ConvNeXt-B	All	10.0 ± 2.0	10.1	7.2	12.1	6
224 → 1024	Epoch time	ViT-B/16	ImageNet	22.1 ± 16.2	16.4	9.6	40.4	3
224 → 1024	Epoch time	ViT-B/16	DINOv2	19.7 ± 1.5	20.1	18	20.8	3
224 → 1024	Epoch time	ViT-B/16	DINOv3	25.3 ± 12.7	18.2	17.8	40.0	3
224 → 1024	Epoch time	ConvNeXt-B	ImageNet	11.2 ± 1.6	12.0	9.4	12.1	3
224 → 1024	Epoch time	ConvNeXt-B	DINOv3	8.8 ± 1.9	8.3	7.2	10.9	3
224 → 1024	Total time to converge	All	All	29.7 ± 26.3	20.9	4.6	94.0	15
224 → 1024	Total time to converge	ViT-B/16	All	42.9 ± 26.4	37.5	17.8	94.0	9
224 → 1024	Total time to converge	ConvNeXt-B	All	9.9 ± 6.2	7.3	4.6	20.9	6
224 → 1024	Total time to converge	ViT-B/16	ImageNet	40.2 ± 26.4	25.2	24.6	70.7	3
224 → 1024	Total time to converge	ViT-B/16	DINOv2	63.3 ± 28.6	58.5	37.5	94.0	3
224 → 1024	Total time to converge	ViT-B/16	DINOv3	25.3 ± 12.7	18.2	17.8	40.0	3
224 → 1024	Total time to converge	ConvNeXt-B	ImageNet	6.9 ± 1.0	6.5	6.1	8.0	3
224 → 1024	Total time to converge	ConvNeXt-B	DINOv3	13.0 ± 8.2	13.6	4.6	20.9	3

Supplementary Table 4: Results for the optimization-regime comparison at 512 × 512 resolution. Results are reported for ChestX-ray14, CheXpert, and MIMIC-CXR, comparing full end-to-end fine-tuning (full FT) and low-rank adaptation (LoRA) for ViT-B/16 and ConvNeXt-B across ImageNet, DINOv2, and DINOv3 initialization strategies. For each dataset and configuration, the table reports mean area under the receiver operating characteristic curve (AUROC) from 1,000 bootstrap resamples as mean ± standard deviation with 95% confidence intervals (CIs). Gap rows summarize the absolute AUROC difference between optimization regimes, defined as full FT − LoRA. Results are shown for all seven datasets: Pedi-CXR (training n=6,955; test n=1,397), VinDr-CXR (training n=15,000; test n=3,000), ChestX-ray14 (training n=77,870; validation n=8,654; test n=25,596), PadChest (training n=79,697; validation n=8,783; test n=22,045), CheXpert (training n=115,449; validation n=13,098; test n=29,318), MIMIC-CXR (training n=153,255; validation n=18,139; test n=43,793), and UKA-CXR (training n=137,902; validation n=15,353; test n=40,106).

Dataset	Initialization	ChestX-ray14	CheXpert	MIMIC-CXR
ViT-B/16 full FT	ImageNet	79.5 ± 0.2 [79.1, 80.0]	80.7 ± 0.2 [80.3, 81.0]	81.4 ± 0.1 [81.1, 81.7]
ViT-B/16 LoRa	ImageNet	76.1 ± 0.2 [75.7, 76.5]	77.7 ± 0.2 [77.4, 78.0]	77.1 ± 0.2 [76.8, 77.5]
Gap: ViT-B/16 full FT − ViT-B/16 LoRa	ImageNet	3.4 ± 0.3 [2.8, 4.0]	3.0 ± 0.2 [2.5, 3.4]	4.2 ± 0.2 [3.8, 4.7]
ViT-B/16 full FT	DINOv3	81.3 ± 0.2 [80.9, 81.8]	81.9 ± 0.2 [81.6, 82.2]	82.5 ± 0.1 [82.2, 82.8]
ViT-B/16 LoRa	DINOv3	76.3 ± 0.2 [75.8, 76.7]	77.6 ± 0.2 [77.2, 77.9]	78.4 ± 0.2 [78.1, 78.7]
Gap: ViT-B/16 full FT − ViT-B/16 LoRa	DINOv3	5.1 ± 0.3 [4.4, 5.7]	4.3 ± 0.2 [3.8, 4.8]	4.1 ± 0.2 [3.7, 4.5]
ViT-B/16 full FT	DINOv2	80.0 ± 0.2 [79.6, 80.4]	81.4 ± 0.2 [81.1, 81.7]	81.2 ± 0.1 [80.9, 81.5]
ViT-B/16 LoRa	DINOv2	78.7 ± 0.2 [78.3, 79.1]	79.2 ± 0.2 [78.8, 79.5]	79.5 ± 0.1 [79.2, 79.8]
Gap: ViT-B/16 full FT − ViT-B/16 LoRa	DINOv2	1.3 ± 0.3 [0.7, 1.9]	2.2 ± 0.2 [1.8, 2.7]	1.7 ± 0.2 [1.3, 2.1]
ConvNeXt-B full FT	ImageNet	81.5 ± 0.2 [81.1, 81.9]	81.6 ± 0.2 [81.2, 81.9]	82.2 ± 0.1 [81.9, 82.4]
ConvNeXt-B LoRa	ImageNet	77.5 ± 0.2 [77.0, 78.0]	78.6 ± 0.2 [78.2, 78.9]	78.8 ± 0.2 [78.5, 79.1]
Gap: ConvNeXt-B full FT − ConvNeXt-B LoRa	ImageNet	4.1 ± 0.3 [3.4, 4.7]	3.0 ± 0.2 [2.6, 3.4]	3.4 ± 0.2 [3.0, 3.8]
ConvNeXt-B full FT	DINOv3	82.3 ± 0.2 [81.8, 82.7]	82.5 ± 0.1 [82.2, 82.8]	82.7 ± 0.2 [82.3, 83.0]
ConvNeXt-B LoRa	DINOv3	79.4 ± 0.2 [79.0, 79.9]	79.2 ± 0.2 [78.9, 79.5]	80.6 ± 0.2 [80.3, 80.9]
Gap: ConvNeXt-B full FT − ConvNeXt-B LoRa	DINOv3	2.8 ± 0.3 [2.2, 3.5]	3.3 ± 0.2 [2.9, 3.7]	2.1 ± 0.2 [1.6, 2.5]

Supplementary Table 5: Exact results for the controlled synthetic label-noise experiment on the VinDr-CXR (training n=15,000; test n=3,000) dataset at 512 × 512 resolution. Results are shown for ViT-B/16 models initialized from ImageNet, DINOv2, and DINOv3 across increasing levels of synthetic training-label corruption from 0% to 40%. For each noise level and initialization strategy, the table reports area under the receiver operating characteristic curve (AUROC), precision-recall area under the curve (PR-AUC), mean average precision (mAP), and retained AUROC relative to the corresponding clean-label (0% noise) setting. All values are reported as mean ± standard deviation with 95% confidence intervals (CIs), based on 1,000 bootstrap resamples.

Metric / Configuration	0% noise	10% noise	20% noise	30% noise	40% noise
ImageNet AUROC	86.4 ± 0.6 [85.2, 87.5]	82.8 ± 0.9 [81.0, 84.6]	77.2 ± 1.0 [75.3, 79.3]	78.8 ± 0.9 [77.1, 80.6]	80.7 ± 0.9 [78.8, 82.4]
DINOv2 AUROC	89.0 ± 0.6 [88.0, 90.2]	86.1 ± 0.7 [84.8, 87.3]	88.6 ± 0.6 [87.3, 89.9]	81.6 ± 1.0 [79.6, 83.7]	80.3 ± 1.0 [78.2, 82.2]
DINOv3 AUROC	90.3 ± 0.5 [89.3, 91.2]	84.7 ± 0.8 [83.1, 86.1]	84.6 ± 0.8 [83.1, 86.0]	78.9 ± 1.0 [77.0, 80.8]	77.9 ± 0.9 [75.9, 79.6]
ImageNet PR-AUC	41.9 ± 1.2 [39.5, 44.5]	37.7 ± 1.1 [35.6, 39.9]	31.6 ± 1.0 [29.6, 33.6]	31.3 ± 1.0 [29.4, 33.1]	32.8 ± 0.9 [31.0, 34.7]
DINOv2 PR-AUC	41.3 ± 1.1 [39.0, 43.4]	35.9 ± 1.0 [33.8, 37.8]	42.2 ± 1.1 [40.1, 44.4]	38.0 ± 1.0 [35.9, 39.9]	36.4 ± 1.1 [34.3, 38.5]
DINOv3 PR-AUC	47.7 ± 1.4 [45.0, 50.3]	40.8 ± 1.1 [38.6, 42.9]	37.3 ± 1.0 [35.4, 39.1]	34.7 ± 1.1 [32.7, 36.8]	28.5 ± 0.9 [26.7, 30.3]
ImageNet mAP	42.5 ± 1.2 [40.1, 45.1]	38.2 ± 1.1 [36.1, 40.4]	32.0 ± 1.0 [30.1, 34.1]	31.8 ± 1.0 [29.9, 33.7]	33.4 ± 1.0 [31.5, 35.2]
DINOv2 mAP	41.9 ± 1.1 [39.6, 44.0]	36.5 ± 1.0 [34.4, 38.5]	42.8 ± 1.1 [40.6, 45.0]	38.5 ± 1.1 [36.4, 40.6]	37.0 ± 1.1 [34.8, 39.2]
DINOv3 mAP	48.3 ± 1.4 [45.7, 50.8]	41.4 ± 1.2 [39.1, 43.9]	37.8 ± 1.0 [35.9, 39.7]	35.2 ± 1.1 [33.1, 37.3]	29.0 ± 0.9 [27.2, 30.9]
ImageNet retained performance vs 0%	100.0 ± 0.0 [100.0, 100.0]	95.9 ± 1.1 [93.7, 98.0]	89.4 ± 1.2 [87.2, 91.8]	91.2 ± 1.0 [89.2, 93.3]	93.5 ± 1.1 [91.2, 95.4]
DINOv2 retained performance vs 0%	100.0 ± 0.0 [100.0, 100.0]	96.7 ± 0.7 [95.2, 98.0]	99.5 ± 0.7 [98.0, 100.9]	91.7 ± 1.1 [89.4, 93.9]	90.2 ± 1.1 [87.9, 92.3]
DINOv3 retained performance vs 0%	100.0 ± 0.0 [100.0, 100.0]	93.8 ± 0.8 [92.0, 95.4]	93.7 ± 0.8 [92.1, 95.3]	87.4 ± 1.1 [85.3, 89.5]	86.3 ± 1.0 [84.1, 88.2]

Supplementary Table 6: Comparison of frozen DINOv3-7B features vs. task-adapted mid-sized models across datasets and resolutions. Average area under the receiver operating characteristic curve (AUROC) derived from 1,000 bootstrap resamples for frozen DINOv3-7B features with lightweight classification heads, compared against the best-performing fully adapted ViT-B/16 and ConvNeXt-B models obtained under full fine-tuning. Results are shown for all seven datasets: Pedi-CXR (training n=6,955; test n=1,397), VinDr-CXR (training n=15,000; test n=3,000), ChestX-ray14 (training n=77,870; validation n=8,654; test n=25,596), PadChest (training n=79,697; validation n=8,783; test n=22,045), CheXpert (training n=115,449; validation n=13,098; test n=29,318), MIMIC-CXR (training n=153,255; validation n=18,139; test n=43,793), and UKA-CXR (training n=137,902; validation n=15,353; test n=40,106), at the universal input resolutions of 224 × 224 and 512 × 512 pixels. For each dataset and resolution, the table reports the frozen DINOv3-7B AUROC as mean ± standard deviation with 95% confidence intervals (CIs), together with the best fine-tuned ViT-B/16 AUROC, the best fine-tuned ConvNeXt-B AUROC, and the identity of the best overall task-adapted model.

Dataset	Resolution	Frozen DINOv3-7B AUROC (mean ± std [95% CI]	Best fine-tuned ViT-B/16 AUROC	Best fine-tuned ConvNeXt-B AUROC	Best overall task-adapted model
Pedi-CXR	224 × 224	68.8 ± 1.3 [66.4, 71.3]	73.4 ± 1.2 [71.1, 75.6]	73.8 ± 1.1 [71.6, 76.0]	ConvNeXt-B (DINOv3)
Pedi-CXR	512 × 512	69.4 ± 1.3 [66.9, 71.8]	74.1 ± 1.2 [72.0, 76.4]	74.0 ± 1.1 [71.8, 76.2]	ViT-B/16 (DINOv2)
VinDr-CXR	224 × 224	82.8 ± 0.8 [81.2, 84.3]	90.2 ± 0.6 [89.0, 91.2]	88.0 ± 0.6 [87.0, 89.1]	ViT-B/16 (DINOv3)
VinDr-CXR	512 × 512	86.8 ± 0.6 [85.5, 88.0]	90.3 ± 0.5 [89.3, 91.2]	90.5 ± 0.5 [89.5, 91.5]	ConvNeXt-B (DINOv3)
ChestX-ray14	224 × 224	76.1 ± 0.2 [75.7, 76.5]	80.1 ± 0.2 [79.7, 80.6]	80.4 ± 0.2 [80.0, 80.8]	ConvNeXt-B (DINOv3)
ChestX-ray14	512 × 512	77.7 ± 0.3 [77.1, 78.2]	81.3 ± 0.2 [80.9, 81.8]	82.3 ± 0.2 [81.8, 82.7]	ConvNeXt-B (DINOv3)
PadChest	224 × 224	84.6 ± 0.2 [84.2, 85.0]	88.0 ± 0.2 [87.6, 88.4]	88.0 ± 0.2 [87.6, 88.3]	ViT-B/16 (DINOv2)
PadChest	512 × 512	86.0 ± 0.2 [85.7, 86.4]	88.9 ± 0.2 [88.6, 89.3]	89.3 ± 0.2 [89.0, 89.7]	ConvNeXt-B (DINOv3)
CheXpert	224 × 224	76.8 ± 0.2 [76.5, 77.2]	80.3 ± 0.2 [80.0, 80.6]	80.5 ± 0.2 [80.2, 80.8]	ConvNeXt-B (DINOv3)
CheXpert	512 × 512	78.9 ± 0.2 [78.5, 79.2]	81.9 ± 0.2 [81.6, 82.2]	82.5 ± 0.1 [82.2, 82.8]	ConvNeXt-B (DINOv3)
MIMIC-CXR	224 × 224	77.1 ± 0.2 [76.8, 77.4]	80.9 ± 0.2 [80.6, 81.2]	81.2 ± 0.1 [80.9, 81.5]	ConvNeXt-B (DINOv3)
MIMIC-CXR	512 × 512	78.9 ± 0.2 [78.6, 79.2]	82.5 ± 0.1 [82.2, 82.8]	82.7 ± 0.2 [82.3, 83.0]	ConvNeXt-B (DINOv3)
UKA-CXR	224 × 224	83.8 ± 0.1 [83.6, 84.1]	88.1 ± 0.1 [87.9, 88.3]	88.2 ± 0.1 [88.0, 88.4]	ConvNeXt-B (DINOv3)
UKA-CXR	512 × 512	85.4 ± 0.1 [85.2, 85.7]	88.5 ± 0.1 [88.2, 88.7]	88.5 ± 0.1 [88.3, 88.7]	ConvNeXt-B (DINOv3)
