# Supplementary material for: Resolution-dependent self-supervised transfer in chest radiograph classification
Source: Commun Med (Lond). 2026 Sep 9;6:479. doi: 10.1038/s43856-026-01897-9 (PMC13558615; doi:10.1038/s43856-026-01897-9)
Supplement: Supplementary file 3 [file 43856_2026_1897_MOESM3_ESM.docx]

**Description of Supplementary Data Files**

**File Names:** Supplementary Data 1, Supplementary Data 2, Supplementary Data 3, Supplementary Data 4, Supplementary Data 5, Supplementary Data 6, Supplementary Data 7, Supplementary Data 8, Supplementary Data 9

**Description:** Source data for figures
